# Chemoenzymatic Generation of Thio-analogues of δ‑Cadinene, δ‑Cadinol, and a Thio-diquinane Using 8‑Thio-farnesylpyrophosphate

**DOI:** 10.1021/acs.jnatprod.5c00409

**Published:** 2025-07-17

**Authors:** Birk Jäger, Jan Luca Budde, Norman Birke, Maximilian Hauke, Andreas Kirschning

**Affiliations:** † Institute of Organic Chemistry, 26555 Leibniz University Hannover, Schneiderberg 1B, 30167 Hannover, Germany; ‡ Uppsala Biomedical Center (BMC), Uppsala University, Husargatan 3, 752 37 Uppsala, Sweden

## Abstract

The synthesis of 8-thio-farnesylpyrophosphate is reported, which was subjected to biotransformations with different sesquiterpene synthases. The 9-thio-analogues of δ-cadinene and δ-cadinol were formed by the sesquiterpene synthases Cop4 and Omp7. In contrast, the fungal sesquiterpene synthase BcBOT2 yielded a diquinane terpenoid that contains a keto and thiol group. The formation of the two former “sulfo”-terpenoids can be rationalized by following the route proposed for δ-cadinene and δ-cadinol. The latter can be regarded by the result of a late-stage hydrolysis product during the analogous pathway to presilphiperfolan-8β-ol, the natural product of BcBOT2. The olfactoric analysis of the three “sulfo”-sesuiterpenoids revealed a stale odor for the cadinene derivatives, whereas the ketothiol diquinane exhibits a spicy aroma with a subtle chili note.

## Graphical abstract

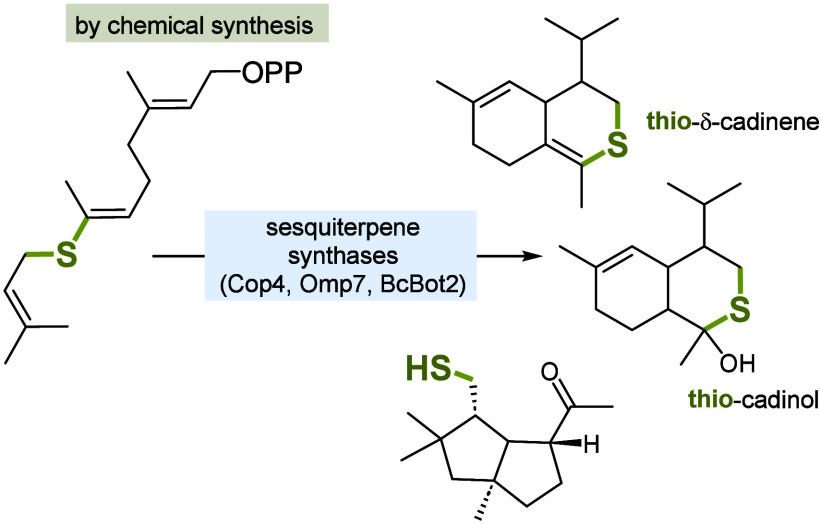

Sulfur-containing compounds can exhibit strong olfactory properties,^1–3^ with *p*-menthene-8-thiol (**1**), 8-mercapto-*p*-menthan-3-one (**2**), and Oxane (**3**) being of particular importance in terms of imparting natural fruitiness and freshness.^4^ In contrast, (3*S*)-3-mercapto-2-methylpentan-1-ol (**4**) is an olfactoric component of raw onion.^5^ Monoterpenoids **1** and **2** belong to the largest and most diverse class of natural products, the terpenes^6^ and are classified according to the number of carbon atoms: Monoterpenes carry 10 carbon atoms, sesquiterpenes 15 and diterpenes 20 carbon atoms, to name just three terpene classes.^7^

In recent years, we^8–16^ and other groups^17–21^ have investigated the substrate promiscuity of terpene synthases responsible for the initiation of carbocationic cyclization cascades by using “unnatural” linear isoprenoid diphosphate precursors. In the case of sesquiterpene synthases (STSs) the linear precursor is farnesylpyrophosphate (FPP, **11**). Remarkable substrate tolerance was observed for some STSs such as the fungal presilphiperfolan-8β-ol synthase (BcBOT2),^22^ the three bacterial Δ^6^-protoilludene synthase (Omp7),^23–25^ pentalenene synthase (PenA),^26–30^ and caryolan-1-ol synthase (GcoA)^31^ as well as the plant derived germacrene A and D synthases (GAS and GDS).^32–34^

Notably, the scope of possible FPP derivatives was also extended to thio-modified FPP derivatives **5** and **6** that were converted by several STSs into “sulfo”-terpenes **7**–**10** (Scheme 1B).^8^

**Scheme 1:**
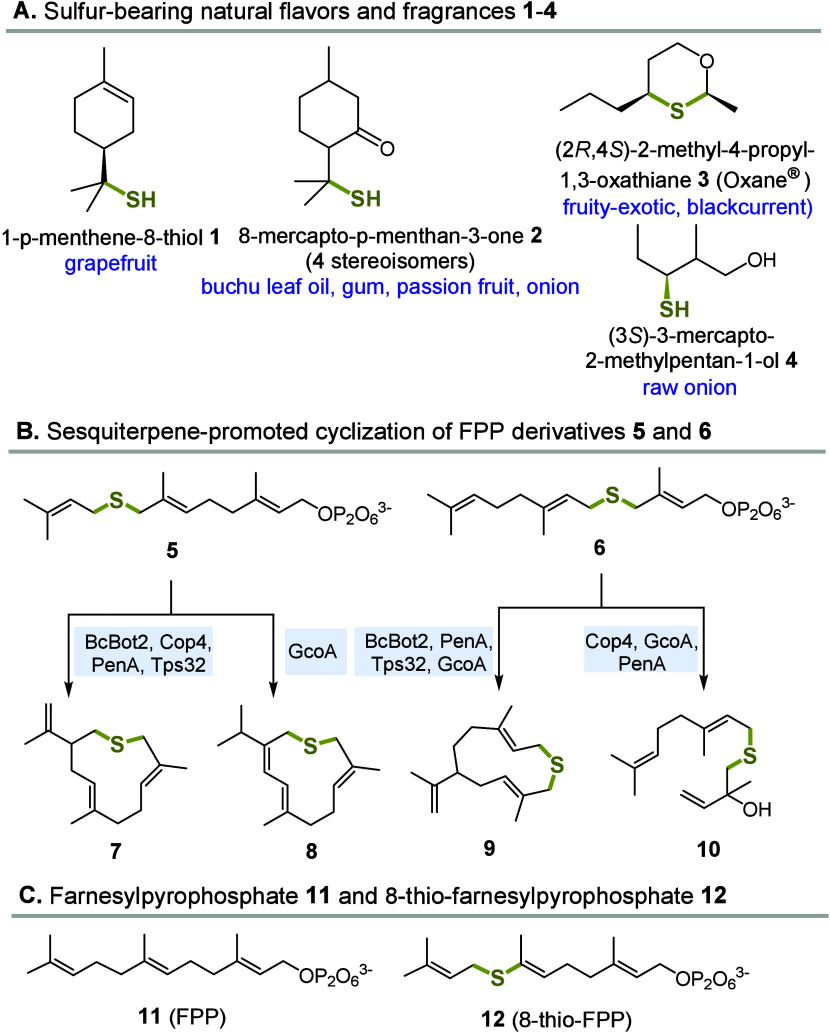
(A) Sulfur-Based Natural Flavors and Volatiles **1**–**4**, (B) “Sulfo”-terpenes **7**–**10** Obtained from STS-Mediated Cyclizations of Thio Ether-FPP Derivatives **5** and **6**, and (C) Structures of FPP **11** and 8-Thio-FPP **12**

Both derivatives **5** and **6** are characterized by the insertion of an additional sulfur atom into the carbon backbone of FPP. Consequently, they are elongated by one atom compared to farnesyl pyrophosphate (**11**). One rationale for this design was based on the fact that sulfur would be attached to two allylic units and the resulting thioether would be chemically stable and easier to synthesize particularly when considering the late stage introduction of the diphosphate moiety. Exchanging one carbon atom by sulfur instead would lead to a vinylthio group, which would stem to be a greater synthetic challenge.^35^ However, we anticipated that cationic cascades will occur that are similar or identical to those observed with FPP **11**, leading to the respective natural sequiterpene skeletons in which one carbon is simply replaced by sulfur. In this context, it should be noted that the bond lengths of the C–C and C–S bonds differ by about 30 pm (C–C = 154 pm vs C–S = 182 pm) and thus altered cationic mechanisms are still conceivable. In this work, we disclose the first synthesis of a vinylthio bearing FPP derivative **12** and its behavior in biotransformations using different sesquiterpene synthases.

## Results and Discussion

### Synthesis of 8-Thio-FPP **12**

We planned to generate the vinylthio-ether group by cross-coupling chemistry of a thiol with a vinyl iodide. There are several examples for such conversions found in the literature, and copper catalysts are known to be good candidates for such couplings.^36^ The synthesis of 8-thio-FPP **12** commenced from geranyl acetate (**13**) whose trisubstituted double bond was oxidatively cleaved using an established two step protocol, starting with an epoxidation followed by a periodate-mediated fragmentation (Scheme 2).^37^

**Scheme 2:**
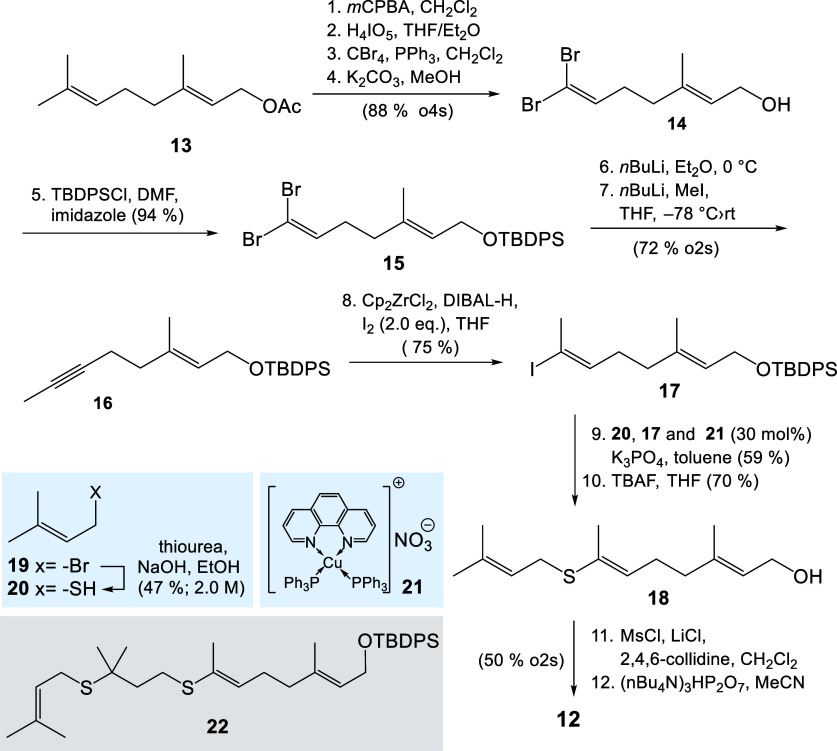
Synthesis of 8-Thio-FPP Derivative **12** (*m*CPBA, *meta*-Chlorobenzoylperoxide; TBDPS, *tert*-Butyldiphenylsilyl; Cp, Cyclopentadienyl Anion, DIBAL-H, Diisobutylaluminum Hydride; TBAF, Tetra-*n*-butylammonium Fluoride Hydrate; Ms, Methylsulfonyl; o2s, over 2 Steps; and o4s, over 4 Steps)

The intermediate aldehyde was subjected to Corey–Fuchs olefination conditions,^38^ which provided 1,1-dibromoalkene **14**. Next, the protecting group had to be exchanged (from acetate to TBDPS) so that the butyl lithium-mediated elimination and Li–Br exchange could be carried out.

This was followed by methylation of the intermediate alkynyl lithium species, which provided alkyne **16**. Next, hydrozirconation using in situ formation of the Schwartz reagent and iodination afforded the vinyl iodide **17**.^39,40^ Copper­(I)-mediated coupling of vinyl iodide **17** with prenylthiol **20** obtained from the corresponding bromide using thiourea as sulfur source, yielded the TBDPS-protected vinylthioether **18**. As isolation of the volatile thiol **20** posed a challenge it was purified by azeotropic distillation (85–100 °C) and the resulting solution was determined to be ∼2.0 M (EtOH/Et_2_O = 7.7:1). The proposed mechanism toward **18** follows the mechanism established for other transition-metal-mediated cross-coupling reactions, especially those with palladium. It is assumed that Cu­(I) temporarily transforms into a Cu­(III) complex after oxidative addition before the product **18** is released by reductive elimination whereby the Cu­(I) catalyst is regenerated. Other proposals envisage a radical mechanism.

In our hands, the introduction of the diphosphate group proved to be less straightforward compared to other FPP derivatives that we had previously prepared.^8–16^ Usually the allyl alcohol is directly transformed into the corresponding allyl chloride, e.g., using *N*-chlorosuccinimide (NCS) and the chloride is then substituted with tris­(tetra-*n*-butylammonium) hydrogen diphosphate as nucleophile. However, formation of the allyl chloride was not observed. This could be due to the fact that the electrophilic chlorine in NCS could also react with the sulfur atom. However, we found that activation of the allyl alcohol is best achieved in two steps by first converting the alcohol into the mesyl ester and in the presence of lithium chloride and 2,4,6-collidine the mesylate is then exchanged by chloride. Interestingly, this strategy was unsuccessful when the tosylate was first generated instead. From the chloride, 8-thio-FPP **12** could be accessed using tris­(tetra-*n*-butylammonium)hydrogen diphosphate and subsequent ion exchange to the ammonium countercation.

At this point, it is worth briefly discussing some of the problems that have arisen up to this point in the synthesis. To achieve high-yielding hydrozirconization with best diastereomeric ratio, the equivalents of zirconocene dichloride had to be increased compared to standard protocols.^39^ The formation of vinyl thioether was accompanied the byproduct **22**, which likely resulted from a thiol–ene reaction between prenyl thiol **20** and the coupling product.^41^

### Biotransformations

Next, we heterologously expressed several STSs as reported before.^8–16^ These included BcBOT2,^22^ Omp7,^23–25^ PenA,^26–30^ GcoA,^31^ and Cop4 (cubebol synthase)^42^ all of which were found to accept FPP derivate **12**. GC–MS analysis revealed the formation of several new products with expected masses of *m*/*z*= 222 (elimination product) and *m*/*z* 240 (water addition product). However, PenA and GcoA showed only a very low turnover and were therefore not considered further in this study (see the Supporting Information). Thus, the experiment was repeated on a larger scale with the STSs Cop4 and BcBOT2 and the three “sulfo”-terpenoids **23**, **24** and **27** were isolated (Scheme 3A). Constitutionally, the former two products resemble the 9-thio analogues of the two known sesquiterpenes δ-cadinene **25** and δ-cadinol **26**. δ-Cadinol is a fungicide and like δ-cadinene it belongs to the family of cadinane sesquiterpenoids.^43^ The structure elucidation was mainly based on different NMR methods (Scheme 3B) and by using authentic samples comparison with data reported for sesquiterpenes **25** and **26** (Scheme 3C).^45^. Diquinane terpenoid **27** ^44^ is structurally related to presilphiperfolan-8β-ol **30**, the main product formed by the fungal STS BcBOT2 except that the cyclohexane ring is absent and a ketone and a thiol group represent that part of the skeleton.^22^ Particularly the relative stereochemistry of ketothiol **27** could be determined by analyzing selected NOESY correlations, and the inclusion of the two geminal methyl groups into the analysis played an important role here. In contrast to the open form present in ketothiol **27**, thio-δ-cadinol **24** consists of a stable hemithioacetal, which means that in principle two anomers could be present in equilibrium. But the NMR spectra of **24** only showed the presence of one diastereomer and no ^13^C-signal could be detected that would indicate the presence of a carbonyl group. Little is known about how pronounced the anomeric effect is with hemithioacetals.^46^ Assuming a preference for the α-anomer, one would suppose a relative stereochemistry at the acetal carbon atom of **24** (see Scheme 4A, bottom left) that is opposite to that at the carbinol center in δ-cadinol **26**. However, NOESY correlations clearly indicate that the methyl group is oriented *syn* toward the two hydrogen atoms at the neighboring annulation site, as is also the case in δ-cadinol **26**. A remarkable aspect with regard to hemithioacetal formation becomes clear when comparing the two biotransformation products **24** and **27**. The latter may in principle also be present as a hemithioacetal, but no analytical evidence for the cyclized product could be found. Steric and stereochemical influences probably play a crucial role here.

**Scheme 3:**
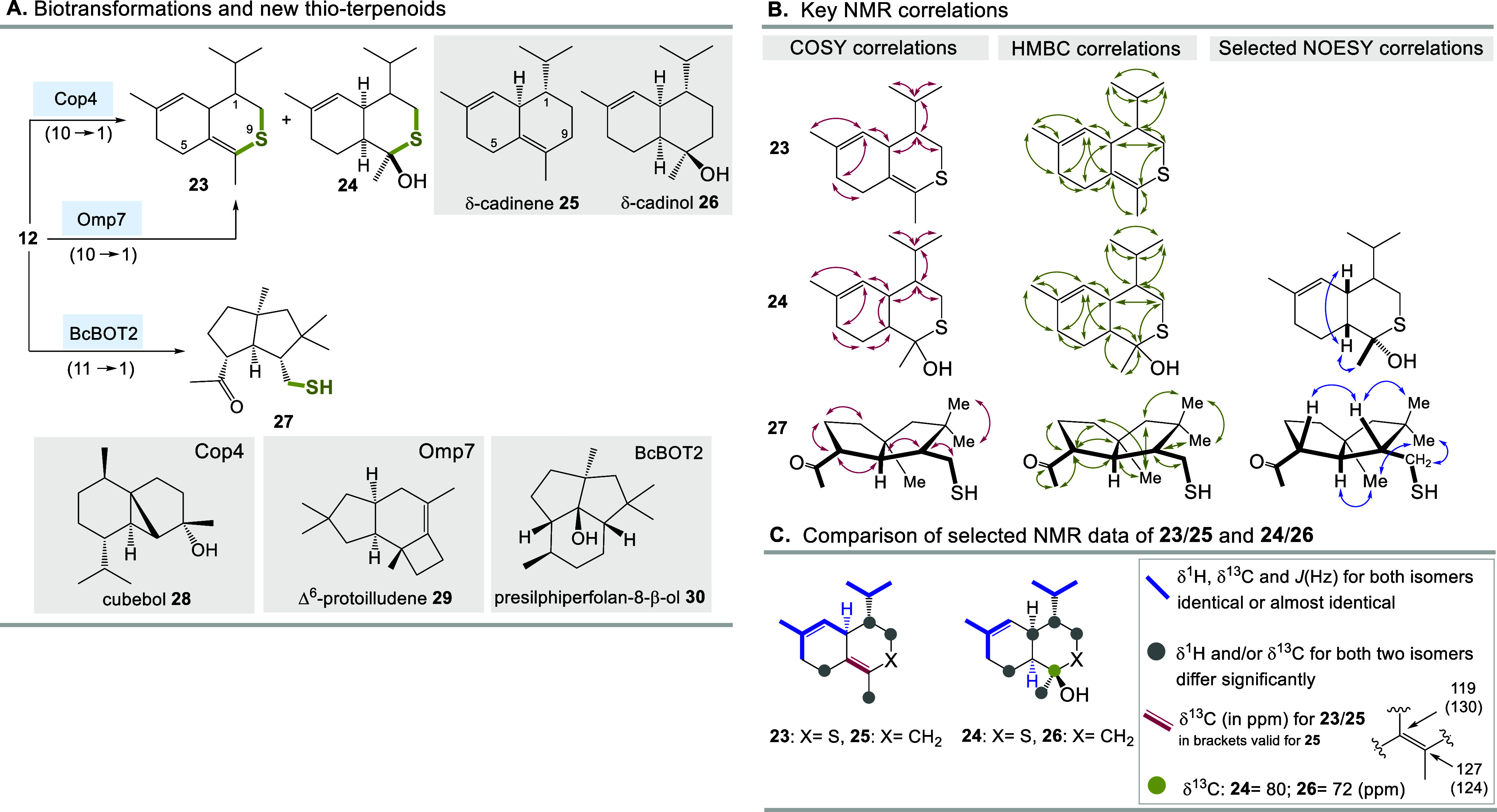
(A) Biotransformation of 8-Thio-FPP **12**, Formation of **23** and **24**, and Structures of δ-Cadinene **25**, δ-Cadinol **26**, and Sesquiterpenes Cubebol **28**, Δ^6^-Protoilludene **29**, and Presilphiperfolan-8-β-ol **30** Naturally Formed by the Three STSs Cop4, Omp7, and BcBOT2, (B) Key NMR Correlations, and (C) Similarities and Difference of Selected NMR Data between **23**/**25** and **24**/**26**

**Scheme 4:**
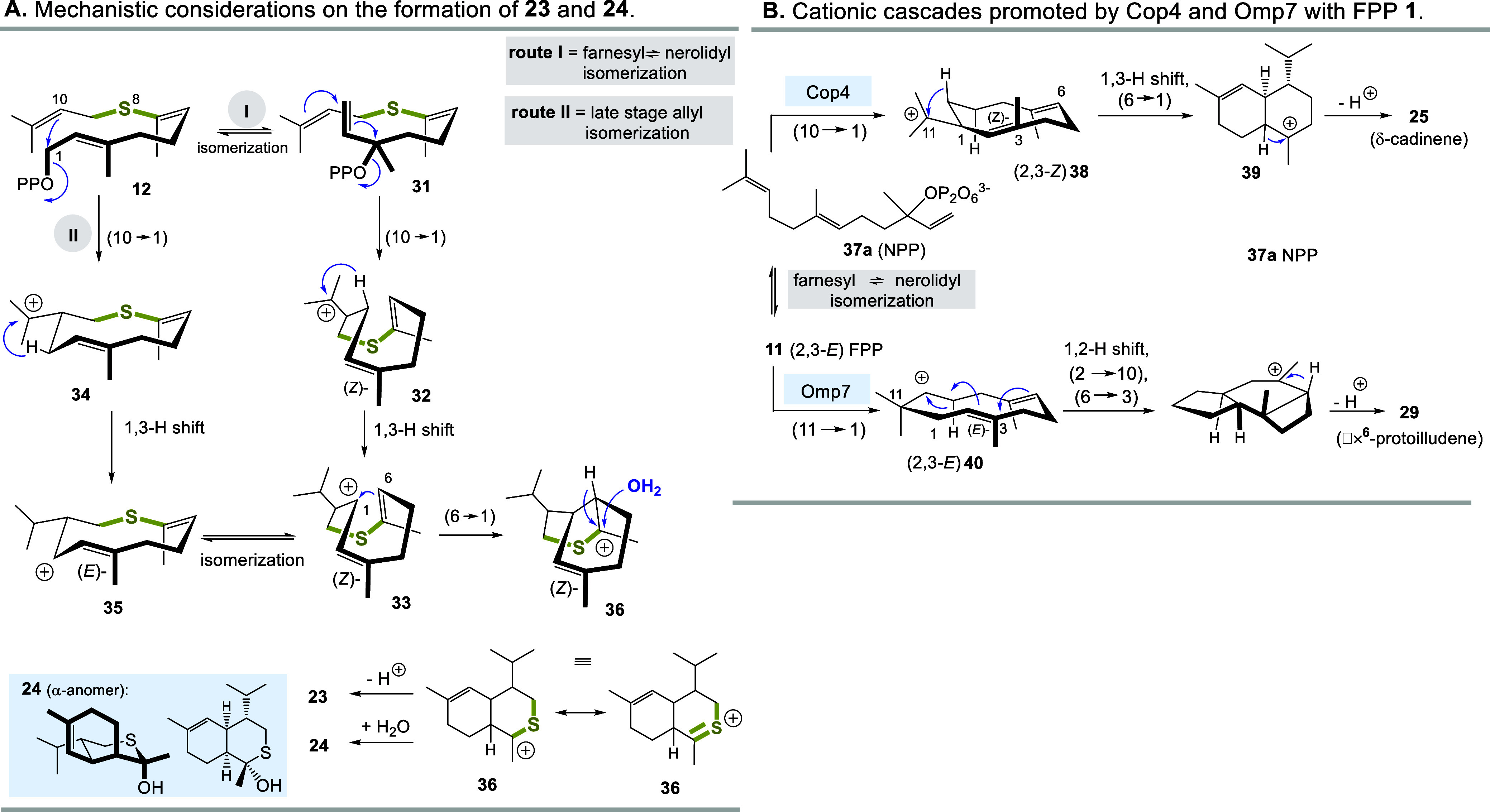
(A) Proposed Mechanisms for the Formation of **23** and **24** and (B) Proposed Mechanism Controlled by STSs Cop4 and Omp7

### Mechanistic Considerations

Mechanistic considerations that can explain the formation of “sulfo”-terpenoids **23** and **24** are closely aligned with the Cop4-mediated transformation of FPP **11** to δ-cadinene **25** (Scheme 4A/B). It is proposed that the known isomerization of (2,3-*E*)-FPP **11** to the NPP isomer **37a** occurs first. After a (10 → 1) cyclization, the carbocation **38** is formed, which then undergoes a 1,3-hydride shift followed by a (6 → 1) cyclization. Proton elimination from carbocation **39** finally yields δ-cadinene **25**. The mechanism for 9-thio-δ-cadinanes **23** and **24** can be described analogously via the intermediates **31**–**35** and **36**. In the final step, the thiocarbenium ion **36** can also be captured by water to form the *O*,*S*-hemiacetal **24**.

The proposed mechanism of Omp7-promoted formation of Δ6 protoilludene **29** does not necessarily have to proceed via the well-known farnesyl nerolidyl isomerization (**11** ⇄ NPP **37a** ⇄ **37b**). An alternative route would start with the (10 → 1) cyclization of **12** and initially yield the cation **34**, which would lead to the allyl cation **35** after a 1,3-hydride shift. This could in principle isomerize and form the *Z* isomer **33**. At this point, the two proposed pathways merge and the final steps are identical. Such “late” isomerizations are chemically plausible, but have not yet been demonstrated in the present context. However, no evidence of early isomerization has yet been described for STS Omp7 either. But there is evidence that the related STS BcBOT2 exhibits this ability.^47^ It was recently found that it converts (2,3-*Z*) **37b** into, albeit in small amounts. This observation can only be explained if the above-mentioned isomerization has occurred first (Scheme 5). Likewise, a similar behavior has been reported by Schmidt-Dannert and co-workers for the STS Cop4.^48^

**Scheme 5:**
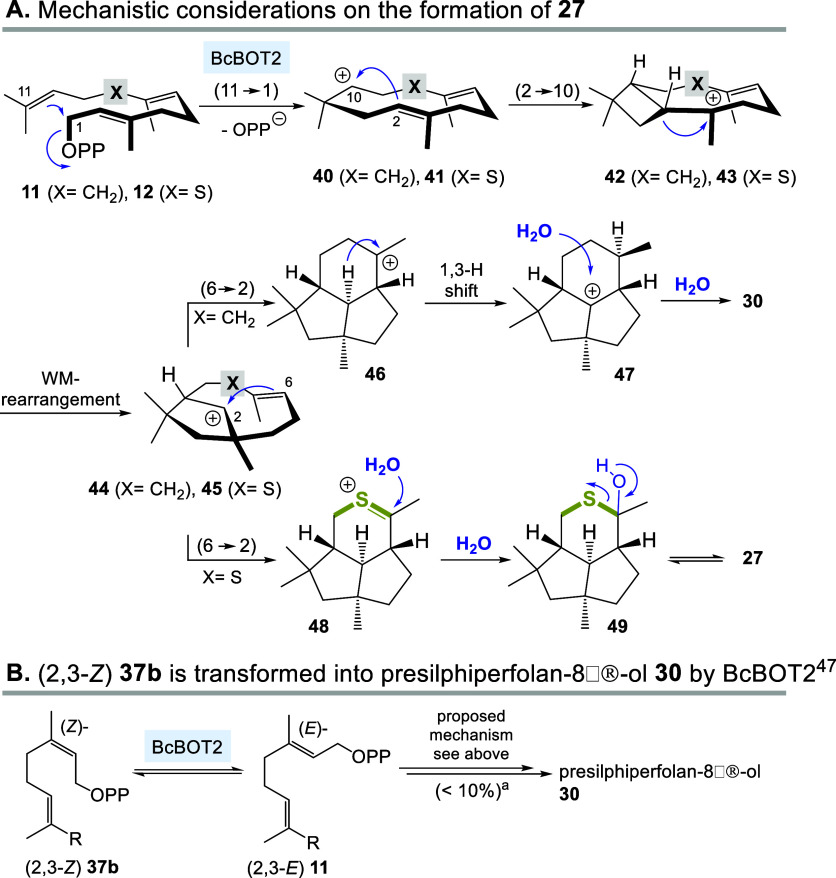
(A) Proposed Mechanisms for the Formation of **27** and Comparison to Proposed Mechanism toward **30** by BcBOT2 and (B) BcBOT2 Converts (2,3-*Z*) Isomer **37b** into **30** via (2,3-*E*) **11** (See Ref 47)

The mechanistic route leading to ketothiol terpenoid **27** most likely follows the proposed mechanism to presilphiperfolan-8β-ol **30** and diverges from it only at the end of the cation cascade (Scheme 5). The process is initiated by two cyclizations (11 → 1 and 2 → 10: from **11** via **40** and **42** and from **12** via **41** and **43**) and a Wagner–Meerwein (MW) rearrangement to yield intermediate carbocations **44** and **45**, respectively. In both cases, the final cyclization (6 → 2) introduces the third six-membered ring and either a new tertiary carbocation **46** or a thiocarbenium ion **48** is formed. The former undergoes a 1,3-hydride shift, and the new carbocation **47** is eventually scavenged by water to form the sesquiterpene **30**. In the other case the thio-stabilized cation **48** is trapped by water to yield hemithioacetal **49**, which is mainly present in the open form **27**, as judged from NMR spectroscopic data (^13^C: δ = 208.7 ppm).

Finally, we carried out preliminary olfactoric determinations for the new thiosesquiterpenes. Terpenoids **23** and **24** exhibit a stale odor with olfactory characteristics of cannabis or rotten eggs. Thiol **27** shows a slightly unpleasant but spicy character tending toward a chili note.

## Conclusion

In summary, we have developed a synthetic approach for 8-thio-farnesyl pyrophosphate **12** containing a highly reactive vinyl thioether moiety. It was found to be a suitable substrate for sesquiterpene synthases and in case of the STSs Cop4 and Omp7 new thio-analogues of δ-cadinene and δ-cadinol were collected.

Here, we provide additional evidence that terpene synthases are surprisingly flexible in dealing with chemically modified linear isoprenyl precursors converting them to oligocyclic terpenoids. This is an astounding finding, considering that evolution has commonly developed one precursor for each of the different terpenes with regard to the number of carbons,^49^ and these are commonly provided until today.^50^

## Experimental Section

### General Experimental Procedures

#### Chemistry

All experiments were performed using an inert argon atmosphere and dry solvents unless otherwise specified. Glassware was dried with a heat gun before use. All commercially available solvents and reagents were used as received. THF was freshly distilled from Na/benzophenone. Flash column chromatography was based silica gel of type 60 M, with a grain size of 40–63 μm. Automated flash column chromatography was conducted with the flash purification system Sepacore by Büchi using prepacked cartridges (PuriFlash by Interchim or Chromabond by Machery–Nagel). The eluents are given in parentheses. For thin-layer chromatography (TLC) aluminum plates coated with silica gel, type 60 F_254_ by Merck were used and the spots were visualized with UV light (*l* = 248 nm) or alternatively by staining with anisaldehyde, vanillin or potassium permanganate solutions. Ion exchange chromatography was performed using the Amberchrom 50WX8 (H^+^ form, 100–200 mesh). A total of 100 g of the material were taken up in water and washed with excess of 6% aqueous NH_3_, until a pH of 14 was reached. It was then washed with distilled water until pH 7 was reached. Then, it was equilibrated with IEB (aqueous 25 mM NH_4_HCO_3_ with 2% iPrOH, ∼100 mL).

^1^H, ^13^C, and ^31^P NMR spectra were recorded with the DPX-400 (400 MHz), AVS-400 (400 MHz), and Ultrashield 500 (500 MHz) by Bruker at 298 K. ^1^H and ^13^C positive chemical shifts (δ) are referenced to CDCl_3_ (^1^H, 7.26 ppm; ^13^C, 77.2 ppm), D_2_O (^1^H, 4.79 ppm) or C_6_D_6_ (^1^H, 7.16 ppm; ^13^C, 128.06 ppm) and are given in parts per million (ppm). Coupling constants (*J*) are given in Hertz (Hz) and reported as observed. The ^1^H NMR data are reported as follows: chemical shift (multiplicity, coupling constants, integral, and assignment). The ^13^C NMR data are reported as follows: chemical shift (assignment). The ^31^P NMR data are reported as follows: chemical shift (multiplicity, coupling constants). NMR assignments are made according to spin systems, using two-dimensional (COSY, HSQC, and HMBC) NMR spectroscopy to assist the assignment. The multiplicities are reported as follows: s, singlet; d, doublet; t, triplet; q, quartet; p, quintet; sxt, sextet; spt, septet; m, multiplet; and br, broad signal.

High-resolution mass spectra (HRMS) were recorded with a Micromass LCT with a lockspray dual ion source in combination with a Waters Alliance 2695 system. Injection was conducted in loop mode. Alternatively, a QTOF premier spectrometer (Waters) in combination with a Waters Acquity UPLC system was used. Ionization was carried out via electrospray ionization (ESI). The calculated and the detected masses are reported. For analytical enzyme tests, an injection volume of 1 μL splitless was used. Lyophilization of the diphosphates was carried out on the Christ ALPHA 2-4 LDC-1 M freeze-drying system at a temperature from −18 to −25 °C and at a pressure of 0.250 mbar.

#### Biology

Heterologous expression in : Heterogeneous expression of STSs was conducted in BL21 (DE3). For the main culture, 50–100 mL of 2TY medium were inoculated with 20% of a preculture. The latter was previously incubated for 16 h at 37 °C and 200 rpm. The main culture was also cultivated at 37 °C and 200 rpm until exponential growth of the cell culture prevailed, reaching an OD_600_ of 0.5–0.8. Induction was then performed with 0.5 mM isopropyl β-d-1-thiogalactopyranoside (IPTG). This is a galactose derivative that can induce the lacUV5 promoter similar to lactose, but unlike lactose, it is not degraded by the cells. The induction leads to the expression of the T7 polymerase, which can subsequently transcribe genes regulated by a T7 promoter. The primary culture was then incubated at 16 °C and 180 rpm for approximately 22 h and followed by centrifugation for 10 min at 5000*g*. The primary culture was then incubated at 16 °C and 180–200 rpm for approximately 22 h and followed by centrifugation for 10 min at 5000*g*, and cell disruption was performed thereafter. Cell pellets not required initially were stored at −20 °C until further use.

To check the expression, samples of 1 mL each were taken from the main culture before induction with IPTG and after 22 h of cultivation. The samples were centrifuged for 10 min at 5000*g*. The pellet was prepared directly for use in a SDS–PAGE and stored at −20 °C.

### Chemical Synthesis of **12**

#### 3-Methylbut-2-ene-1-thiol (**20**)

To a solution of prenyl bromide (**19**, 4.47 g, 30.0 mmol, 1.00 equiv) in EtOH (24.0 mL) was added thiourea (2.28 g, 30.0 mmol, 1.00 equiv). The mixture was warmed to 80 °C and stirred for 3 h. NaOH (aqueous, 10.0 wt %, 24.0 mL) was added, and the mixture was stirred for an additional 3 h at 70 °C. The reaction mixture was cooled to rt and diluted with Et_2_O (25.0 mL). The phases were separated and HCl (aqueous, 1.00 M) was added to acidify the aqueous phase to pH 2. It was then extracted with Et_2_O (3 × 20.0 mL) and the combined organic phases were dried over MgSO_4_ and filtered. The crude material was purified via distillation (80–100 °C at 1 bar) to afford **20** (1.43 g, 14.0 mmol, 46.8%) as a colorless liquid (2.03 M solution in EtOH/Et_2_O).

*R*_f_ (EtOAc in hexanes, 12.5%): 0.6; ^1^H NMR (400 MHz, CDCl_3_) δ = 5.28 (tsxt, *J* = 7.8, 1.2 Hz, 1H, C3–H), 3.10 (t, *J* = 7.5 Hz, 2H, C5–H), 1.67 (s, 3H, C4–H), 1.61 (s, 3H, C1–H) ppm; ^13^C NMR (101 MHz, CDCl_3_) δ = 134.1 (C2), 123.4 (C3), 25.6 (C4), 22.2 (C5), 17.4 (C1) ppm.

#### (*E*)-5-(3,3-Dimethyloxiran-2-yl)-3-methylpent-2-en-1-yl Acetate (**S1**)

Geranyl acetate **13** (1.96 g, 10.0 mmol, 1.00 equiv) was dissolved in CH_2_Cl_2_ (13.0 mL) at rt. The mixture was then cooled to 0 °C and *m*CPBA (70 wt %, 2.71 g, 11.0 mmol, 1.10 equiv) was added slowly. The solution was stirred for 2.5 h at 0 °C, and the reaction was terminated by addition of H_2_O (15.0 mL). The phases were separated, and the aqueous phase was extracted with EtOAc (3 × 15.0 mL). Subsequently, the combined organic phases were washed with brine (60.0 mL), dried over MgSO_4_, filtered, and concentrated under reduced pressure. The residue was purified by flash column chromatography on silica gel (EtOAc in hexanes, 17%) affording **S1** (1.72 g, 8.09 mmol, 80.9%) as a colorless oil.

*R*_f_ (EtOAc in hexanes, 17%): 0.6; ^1^H NMR (400 MHz, CDCl_3_) δ = 5.38 (tsxt, *J* = 7.1, 1.3 Hz, 1H, C9–H), 4.99 (d, *J* = 7.1 Hz, 2H, C10–H), 2.70 (t, *J* = 6.2 Hz, 1H, C4–H), 2.11–2.26 (m, 2H, C6–H), 2.05 (s, 3H, C12–H), 1.72 (s, 3H, C8–H), 1.63–1.69 (m, 2H, C5–H), 1.30 (s, 3H, C1–H/C3-H), 1.26 (s, 3H, C1–H/C3-H) ppm; ^13^C NMR (101 MHz, CDCl_3_) δ = 171.2 (C11), 141.4 (C7), 119.0 (C9), 64.0 (C4), 61.4 (C10), 58.5 (C2), 36.3 (C6), 27.2 (C5), 25.0 (C1/C3), 21.2 (C12), 18.9 (C1/C3), 16.6 (C8) ppm.

Analytical data are in agreement with those reported in literature.^51^

#### (*E*)-3-Methyl-6-oxohex-2-en-1-yl Acetate (**S2**)

Periodic acid (2.21 g, 9.71 mmol, 1.20 equiv) was dissolved in THF (72.8 mL) and cooled to 0 °C. Epoxide **S1** (1.72 g, 8.09 mmol, 1.00 equiv) in Et_2_O (12.1 mL) was then added and the mixture was stirred for 1.5 h. The reaction mixture was diluted with Et_2_O (50.0 mL) and the phases were separated. The organic phase was washed with saturated aqueous NaHCO_3_ (100 mL) and brine (100 mL), dried over MgSO_4_, filtered, and concentrated under reduced pressure to afford **S2** (isolated yield not determined) as a light yellow oil. It was used for the next step without further purification.

*R*_f_ (EtOAc in hexanes, 10%): 0.8; ^1^H NMR (400 MHz, CDCl_3_) δ = 9.77 (s, 1H, C1–H), 5.36 (tsxt, *J* = 7.1, 1.3 Hz, 1H, C6–H), 4.57 (d, *J* = 7.1 Hz, 2H, C7–H), 2.55–2.60 (m, 2H, C3–H), 2.35–2.40 (m, 2H, C2–H), 2.05 (s, 3H, C9–H), 1.72 (s, 3H, C5–H) ppm; ^13^C NMR (101 MHz, CDCl_3_) δ = 201.8 (C1), 171.2 (C8), 140.1 (C4), 119.5 (C6), 61.2 (C7), 41.9 (C2), 32.0 (C3), 21.2 (C9), 16.7 (C5) ppm.

NMR spectroscopic data are in agreement with those reported in literature.^51^

#### (*E*)-7,7-Dibromo-3-methylhepta-2,6-dien-1-yl Acetate (**S3**)

A solution of PPh_3_ (36.7 g, 140 mmol, 2.80 equiv) in CH_2_Cl_2_ (250 mL) was cooled to 0 °C, before CBr_4_ (23.2 g, 70.0 mmol, 1.40 equiv) was added. The mixture was stirred for 30 min at 0 °C and for additional 30 min at rt. Then, the mixture was cooled to 0 °C, before a solution of **S2** (8.51 g, 50.0 mmol, 1.00 equiv) in CH_2_Cl_2_ (25.0 mL) was added. The reaction mixture was stirred for 1.5 h, before being diluted using PE (500 mL). The mixture was filtered through a pad of Celite. The residue was dissolved in CH_2_Cl_2_ (30.0 mL), before PE (300 mL) was added. It was again filtered through a pad of Celite. This was repeated two additional times, before the filtrate was concentrated under reduced pressure and filtered through silica, affording **S3** (isolated yield not determined) as a yellow oil.

*R*_f_ (EtOAc in hexanes, 10%): 0.8; ^1^H NMR (400 MHz, CDCl_3_) δ = 6.35 (t, *J* = 6.9 Hz, 1H, C2–H), 5.36 (tsxt, *J* = 7.1, 1.3 Hz, 1H, C7–H), 4.59 (d, *J* = 7.1 Hz, 2H, C8–H), 2.21–2.27 (m, 2H, C4–H), 2.12–2.16 (m, 2H, C3–H), 2.06 (s, 3H, C10–H), 1.71 (s, 3H, C6–H) ppm; ^13^C NMR (101 MHz, CDCl_3_) δ = 171.2 (C9), 140.5 (C5), 137.8 (C1), 119.8 (C7), 89.4 (C2), 61.3 (C8), 37.3 (C3), 31.2 (C4), 21.2 (C10), 16.5 (C6) ppm.

NMR spectroscopic data are in agreement with those reported in literature.^51^

#### (*E*)-7,7-Dibromo-3-methylhepta-2,6-dien-1-ol (**14**)

Acetate **S3** (13.2 g, 40.5 mmol, 1.00 equiv) was dissolved in MeOH (243 mL) at rt. No precautions were taken to ensure dry or inert conditions. K_2_CO_3_ (6.16 g, 44.6 mmol, 1.10 equiv) was added, and the mixture was stirred for 30 min at rt. CH_2_Cl_2_ (250 mL) was added, and the phases were separated. The aqueous phase was extracted with CH_2_Cl_2_ (3 × 100 mL), and the combined organic phases were washed with brine (400 mL), dried over MgSO_4_, filtered, and concentrated under reduced pressure to afford **14** (10.1 g, 35.6 mmol, 88.0% o4s) as a colorless oil. It could be employed in the next step without further purification.

*R*_f_ (EtOAc in hexanes, 10%): 0.02; ^1^H NMR (400 MHz, CDCl_3_) δ = 6.37 (t, *J* = 6.8 Hz, 1H, C2–H), 5.43 (tsxt, *J* = 6.9, 1.3 Hz, 1H, C7–H), 4.17 (d, *J* = 6.7 Hz, 2H, C8–H), 2.21–2.27 (m, *J* = 2H, C4–H), 2.11–2.15 (m, 2H, C3–H), 1.69 (m, 3H, C6–H), 1.58 (brs, 1H, C8-OH) ppm; ^13^C NMR (101 MHz, CDCl_3_) δ 138.0 (C1), 124.7 (C5), 89.2 (C2), 59.5 (C8), 37.4 (C3), 31.3 (C4), 16.3 (C6) ppm.

NMR spectroscopic data are in agreement with those reported in literature.^51^

#### (*E*)-*tert*-Butyl­((7,7-dibromo-3-methylhepta-2,6-dien-1-yl)­oxy)­diphenylsilane (**15**)

Alcohol **14** (10.1 g, 35.6 mmol, 1.00 equiv) was dissolved in DMF (35.6 mL). Imidazole (5.34 g, 78.4 mmol, 2.20 equiv) was added, and the mixture was stirred for 10 min at rt, before being cooled down to 0 °C. TBDPSCl (8.05 g, 53.4 mmol, 1.50 equiv) was added; the mixture was stirred for 16 h; and the reaction was terminated by addition of H2O (35.0 mL). The phases were separated, and the aqueous phase was extracted with CH_2_Cl_2_ (3 × 35.0 mL). The combined organic phases were washed with brine (125 mL), dried over MgSO_4_, filtered, and concentrated under reduced pressure. The residue was purified by flash column chromatography on silica gel (EtOAc in hexanes, 5%) affording **25** (17.5 g, 33.5 mmol, 94.2%) as a colorless oil (containing 13.2 mol % TBDPSOH as an impurity).

NMR spectroscopic data are in agreement with those reported in literature.^51^

#### (*E*)-*tert*-Butyl­((3-methylhept-2-en-6-yn-1-yl)­oxy)­diphenylsilane (**S4**)

Dibromoalkene **15** (2.29 g, 4.39 mmol, 1.00 equiv) was dissolved in Et_2_O (50.0 mL), and the solution was cooled to 0 °C. *n*BuLi (2.0 M in hexane, 4.83 mL, 9.66 mmol, 2.20 equiv) was slowly added, and the mixture was stirred for 1 h at 0 °C. The reaction was terminated by the addition of H_2_O (50.0 mL). The phases were separated, and the aqueous phase was extracted with Et_2_O (3 × 50.0 mL). The combined organic phases were washed with brine (200 mL), dried over MgSO_4_, filtered, and concentrated under reduced pressure. The residue was purified by flash column chromatography on silica gel (CH_2_Cl_2_ in hexanes, 20%) affording **S4** as a light yellow oil (1.32 g, 3.51 mmol, 80.0%).

*R*_f_ (CH_2_Cl_2_ in hexanes, 20%): 0.51; ^1^H NMR (400 MHz, CDCl_3_) δ = 7.68–7.71 (m, 4H, C10–H), 7.36–7.42 (m, 6H, C11–H, C12–H), 5.43 (tsxt, *J* = 6.3 Hz, 1.2 Hz, 1H, C7–H), 4.23 (d, *J* = 6.3 Hz, 2H, C8–H), 2.24–2.29 (m, 2H, C4–H), 2.17–2.22 (m, 2H, C3–H), 1.94 (t, *J* = 2.6 Hz, 1H, C1–H), 1.44 (s, 3H, C6–H), 1.04 (s, 9H, C13–(CH_3_)_3_) ppm; ^13^C NMR (101 MHz, CDCl_3_) δ = 135.8 (C5), 135.2 (C9), 134.1 (C12) 129.7 (C10/C11), 127.7 (C10/C11), 125.5 (C7), 84.2 (C2), 68.6 (C1), 61.1 (C8), 38.3 (C4), 27.0 (C13–(CH_3_)_3_), 19.3 (C13), 17.4 (C3), 16.6 (C6) ppm; HRMS [ESI] *m*/*z* 363.2127 [M + Na]^+^ (calculated for C_24_H_30_OSiNa^+^ 363.2144).

#### (*E*)-*tert*-Butyl­((3-methyloct-2-en-6-yn-1-yl)­oxy)­diphenylsilane (**16**)

##### Procedure 1

**S4** (169 mg, 0.320 mmol, 1.00 equiv) was dissolved in THF (0.809 mL), and the solution was cooled to 0 °C. *n*BuLi (2.15 M in hexane, 0.380 mL, 0.810 mmol, 2.50 equiv) was slowly added, and the mixture was stirred for 1.5 h at rt. Then, the mixture was cooled back down to 0 °C before slow addition of a second portion of *n*BuLi (2.15 M in hexane, 0.170 mL, 0.360 mmol, 1.10 equiv). It was then stirred for 45 min at 0 °C, before addition of MeI (459 mg, 0.200 mL, 3.24 mmol, 10.0 equiv) and stirring was continued for an additional 45 min at 0 °C. Then, it was warmed to rt while stirring for 3 h. The reaction was then terminated by addition of aqueous saturated NH_4_Cl (1.00 mL). The phases were separated, and the aqueous phase was extracted with Et_2_O (3 × 1.00 mL). The combined organic phases were washed with brine (5.00 mL), dried over MgSO_4_, filtered, and concentrated under reduced pressure. The residue was purified by flash column chromatography on silica gel (CH_2_Cl_2_ in hexanes, 20%) affording **16** (43.6 mg, 0.116 mmol, 35.8%) as a light yellow oil.

##### Procedure 2

**S4** (1.11 g, 3.05 mmol, 1.00 equiv) was dissolved in THF (9 mL), and the solution was cooled to −78 °C. *n*BuLi (2.00 M in hexanes, 1.68 mL, 3.35 mmol, 1.10 equiv) was added, and the mixture was stirred for 20 min at −78 °C. After addition of MeI (4.33 g, 1.90 mL, 30.5 mmol, 10.0 equiv), the mixture was slowly warmed to rt and stirring was continued for 1 h. The reaction was terminated by addition of H_2_O (10.0 mL). The phases were separated, and the aqueous phase was extracted with Et_2_O (3 × 10.0 mL). The combined organic phases were washed with brine (50.0 mL), dried over MgSO_4_, filtered, and concentrated under reduced pressure. The residue was purified by flash column chromatography on silica gel (CH_2_Cl_2_ in hexanes, 20%) affording **16** (996 mg, 2.65 mmol, 71.4%) as a light yellow oil.

*R*_f_ (CH_2_Cl_2_ in hexanes, 20%): 0.51; ^1^H NMR (400 MHz, CDCl_3_) δ = 7.68–7.70 (m, 4H, C11–H), 7.35–7.44 (m, 6H, C12–H, C13–H), 5.42 (tsxt, *J* = 6.3 Hz, 1.2 Hz, 1H, C8–H), 4.22 (d, *J* = 6.4 Hz, 2H, C9–H), 2.18–2.23 (m, 2H, C5–H), 2.13–2.16 (m, 2H, C4–H), 1.76 (t, *J* = 2.4 Hz, 3H, C1–H), 1.43 (s, 3H, C7–H), 1.04 (s, 9H, C14–(CH_3_)_3_) ppm; ^13^C NMR (101 MHz, CDCl_3_) δ = 135.8 (C11), 134.2 (C6), 129.7 (C10), 127.7 (C12, C13), 125.1 (C8), 79.0 (C3), 75.9 (C2), 61.2 (C9), 39.0 (C5), 26.7 (C14–(CH_3_)_3_), 19.3 (C14), 17.8 (C4), 16.3 (C7), 3.6 (C1) ppm.

The spectroscopic and analytical data are in accordance with those reported in the literature.^52^

#### *tert*-Butyl-(((2*E*,6*E*)-7-iodo-3-methylocta-2,6-dien-1-yl)­oxy)­diphenylsilane (**17**)

Zirconocene dichloride (4.26 g, 14.6 mmol, 2.75 equiv) was dissolved in THF (29.2 mL), and the solution was cooled to 0 °C. DIBAL-H (1.00 M in hexanes, 14.6 mL, 14.6 mmol, 2.75 equiv) was added, and the mixture was stirred for 1 h at 0 °C. A solution of alkyne **16** (2.00 g, 5.28 mmol, 1.00 equiv) in THF (2.64 mL) was added, and the reaction mixture was stirred for 16 h at rt. Next iodine (2.68 g, 10.6 mmol, 2.00 equiv) was added, and the mixture was stirred for 15 min at rt, before the reaction was terminated by addition of 1.00 M aqueous HCl (15.0 mL). The phases were separated, and the aqueous phase was extracted with Et_2_O (3 × 15.0 mL). The combined organic phases were washed with saturated aqueous Na_2_S_2_O_3_ (60.0 mL) and brine (60.0 mL), dried over MgSO_4_, filtered, and concentrated under reduced pressure. It was then purified by flash column chromatography (CH_2_Cl_2_ in hexanes, 12%) to afford the vinyl iodide (dr 6:1). The diastereoisomers were separated by flash column chromatography (CH_2_Cl_2_ in hexanes, 6.3%) to afford the desired isomer **17** (1.25 g, 2.38 mmol, 44.7%, dr 12:1) as a colorless oil.

*R*_f_ (CH_2_Cl_2_ in hexanes, 12%): 0.5; ^1^H NMR (400 MHz, CDCl_3_) δ = 7.71–7.73 (m, 4H, C11–H), 7.38–7.45 (m, 6H, C–12H, C13–H), 6.15 (tsxt, *J* = 7.3, 1.5 Hz, 1H, C3–H), 5.40 (tsxt, *J* = 6.3, 1.2 Hz, 1H, C8–H), 6.24 (d, *J* = 6.3 Hz, 2H, C9–H), 2.39 (m, 3H, C1–H), 2.11–2.16 (m, 2H, C5–H), 2.01–2.06 (m, 2H, C4–H), 1.45 (s, 3H, C7–H), 1.07 (s, 9H, C14–(CH_3_)_3_) ppm; ^13^C NMR (101 MHz, CDCl_3_) δ = 140.8 (C9), 136.7 (C11), 134.2 (C13), 129.7 (C10), 127.8 (C12), 125.0 (C8), 93.8 (C2), 61.2 (C9), 53.6 (C6), 38.5 (C4), 27.7 (C1), 29.1 (C5), 27.0 (C14–(CH_3_)_3_), 19.3 (C14), 16.4 (C7) ppm.

Analytical data are in agreement with those reported in literature.^52^

#### *tert*-Butyl-(((2*E*,6*E*)-3-methyl-7-((3-methylbut-2-en-1-yl)­thio)­octa-2,6-dien-1-yl)­oxy)­diphenylsilane (**S5**) and (6*E*,10*E*)-2,2,7,11,15,15,19-Heptamethyl-3,3-diphenyl-4-oxa-12,16-dithia-3-silaicosa-6,10,18-triene (**22**)

Vinyl iodide **17** (1.20 g, 2.38 mmol, 1.0 equiv) and thiol **20** (267 mg, 2.61 mmol, 1.10 equiv) were dissolved in toluene (10.5 mL) and degassed under a stream of argon for 15 min. Then, [Cu­(phen)­(PPh_3_)_2_]­NO_3_ (**21**, 651 mg, 0.714 mmol, 30.0 mol %) and K_3_PO_4_ (908 mg, 4.28 mmol, 1.80 equiv) were added, and the reaction mixture was stirred for 16 h at 80 °C, before the reaction was terminated by addition of H_2_O (10.5 mL). The phases were separated, and the aqueous phase was extracted with EtOAc (3 × 12.0 mL). The combined organic phases were washed with brine (50.0 mL), dried over MgSO_4_, filtered, and concentrated under reduced pressure. It was then purified by flash column chromatography (CH_2_Cl_2_ in hexanes, 25%) to afford **S5** (975 mg, 2.04 mmol, 77.9%) as a colorless oil.

*R*_f_ (CH_2_Cl_2_ in hexanes, 15%): 0.5; ^1^H NMR (400 MHz, C_6_D_6_) δ = 7.83–7.85 (m, 4H, C16–H), 7.23–7.28 (m, 6H, C17–H, C18–H), 5.59 (tq, *J* = 6.3, 1.3 Hz, 1H, C13–H), 5.45 (tq, *J* = 7.1, 1.3 Hz, 1H, C4–H), 5.35 (tspt, *J* = 7.6, 1.5 Hz, 1H, C8–H), 4.35 (d, *J* = 6.4 Hz, 2H, C14–H), 3.29 (d, *J* = 7.6 Hz, 2H, C5–H), 2.05–2.11 (m, 2H, C10–H), 1.91–1.95 (m, 2H, C9–H), 1.82 (m, 3H, C7–H), 1.56 (s, 3H, C1–H), 1.49 (s, 3H, C3–H), 1.30 (s, 3H, C12–H), 1.20 (s, 9H, C19–(CH_3_)_3_) ppm; ^13^C NMR (101 MHz, C_6_D_6_) δ = 136.7 (C2), 136.1 (C11), 135.2 (C16), 134.5 (C18), 130.8 (C6), 129.9 (C15), 128.1 (C17), 125.8 (C4), 125.0 (C13), 120.4 (C8), 61.5 (C5), 39.5 (C9), 30.1 (C14), 27.5 (C10), 27.1 (C19–(CH_3_)_3_), 25.7 (C1), 19.5 (C19), 18.2 (C7), 17.7 (C3), 16.3 (C12) ppm; HRMS [ESI] *m*/*z* 501.2614 [M + Na]^+^ (calculated for C_30_H_42_OSSiNa^+^ [M + Na]^+^: 501.2604).

Compound **22** was isolated as a second byproduct by flash column chromatography (CH_2_Cl_2_ in hexanes, 25 to 50%) as a light yellow oil (74.2 mg, 0.162 mmol, 13.8%).

*R*_f_ (CH_2_Cl_2_ in hexanes, 15%): 0.25; ^1^H NMR (400 MHz, C_6_D_6_) δ = 7.83–7.85 (m, 4H, C22–H), 7.23–7.28 (m, 6H, C23–H, C24–H), 5.55–5.60 (m, 2H, C13–H, C18–H), 5.31 (tspt, *J* = 7.7, 1.4 Hz, 1H, C4–H), 4.35 (d, *J* = 6.3 Hz, 2H, C19–H), 3.04 (d, *J* = 7.8 Hz, 2H, C5–H), 2.86–2.90 (m, 2H, C10–H), 2.06–2.12 (m, 2H, C14–H), 1.91–1.95 (m, 2H, C15–H), 1.81–1.86 (m, 5H, C12–H, C9–H), 1.57 (s, 3H, C3–H), 1.53 (s, 3H, C1–H), 1.30 (s, 3H, C17–H), 1.20 (s, 9H, C12–(CH_3_)_3_), 1.15 (s, 6H, C7–H, C8–H) ppm; ^13^C NMR (101 MHz, C_6_D_6_) δ = 136.3 (C16), 135.7 (C22), 134.4 (C24), 134.1 (C2), 129.8 (C21), 129.6 (C11), 128.1 (C23), 125.8 (C13/C18), 124.7 (C13/C18), 120.6 (C4), 61.1 (C19), 44.9 (C6), 41.5 (C9), 39.5 (C15), 28.6 (C7, C8), 27.6 (C10), 27.2 (C20–(CH_3_)_3_), 26.8 (C14), 26.1 (C5), 25.3 (C3), 19.1 (C20), 17.7 (C12), 17.3 (C1), 16.0 (C17) ppm; HRMS [ESI] *m*/*z* 603.3127 [M + Na]^+^ (calculated for C_35_H_50_OS_2_SiNa^+^ [M + Na]^+^: 603.3126).

#### (2*E*,6*E*)-3-Methyl-7-((3-methylbut-2-en-1-yl)­thio)­octa-2,6-dien-1-ol (**18**)

**S5** (975 mg, 2.58 mmol, 1.00 equiv) was dissolved in THF (12.9 mL). No precautions were taken to ensure dry or inert conditions. TBAF (1.00 M in THF, 3.09 mL, 3.09 mmol, 1.20 equiv) was added, and the mixture was stirred for 1 h at rt, before the reaction was terminated by addition of H_2_O (10.0 mL). The phases were separated, and the aqueous phase was extracted with EtOAc (3 × 15.0 mL). The combined organic phases were washed with brine (50.0 mL), dried over MgSO_4_, filtered, and concentrated under reduced pressure (up to 250 mbar). It was then purified by flash column chromatography (Et_2_O in hexanes, 40%) to afford **18** (433 mg, 1.80 mmol, 69.9%) as a colorless oil. It was not possible to remove all of the solvent due to the volatile nature of the product.

*R*_f_ (Et_2_O in hexanes, 50%): 0.65; ^1^H NMR (400 MHz, C_6_D_6_) δ = 5.44 (tsxt, *J* = 7.14, 1.3 Hz, 1H, C4–H), 5.32–5.38 (m, 2H, C8–H, C13–H), 5.97 (d, *J* = 6.7 Hz, 2H, C14–H), 3.28 (d, *J* = 7.6 Hz, 2H, C5–H), 2.06–2.11 (m, 2H, C10–H), 1.91–1.95 (m, 2H, C9–H), 1.83 (m, 3H, C7–H), 1.56 (s, 3H, C1–H), 1.50 (s, 3H, C3–H), 1.44 (s, 3H, C12–H) ppm; ^13^C NMR (101 MHz, C_6_D_6_) δ = 137.6 (C11), 135.3 (C2), 130.9 (C6), 125.7 (C4), 125.3 (C8/C13), 120.4 (C8/C13), 59.3 (C5), 39.5 (C9), 30.1 (C14), 27.5 (C10), 25.6 (C1), 18.2 (C7), 17.7 (C3), 16.1 (C12) ppm.

#### ((2*E*,6*E*)-8-Chloro-6-methylocta-2,6-dien-2-yl)­(3-methylbut-2-en-1-yl)­sulfane (**S6**)

2,4,6-Collidine (145 mg, 0.159 mL, 1.20 mmol, 3.00 equiv) and LiCl (250 mg, 5.90 mmol, 14.7 equiv) were dissolved in CH_2_Cl_2_ (2.70 mL) and cooled to 0 °C. The mixture was cooled to 0 °C, before addition of MsCl (101 mg, 0.068 mL, 0.880 mmol, 2.20 equiv). The reaction mixture was stirred for 10 min at 0 °C before addition of **18** (96.2 mg, 0.400 mmol, 1.00 equiv) in minimal CH_2_Cl_2_. The mixture was then stirred for 3 h at 0 °C, before the reaction was terminated by addition of a saturated aqueous NaHCO_3_ solution (3.00 mL). The phases were separated, and the aqueous phase was extracted with CH_2_Cl_2_ (3 × 3.00 mL). The combined organic phases were washed with H_2_O (2 × 15.0 mL) and brine (15.0 mL), dried over MgSO_4_, filtered, and concentrated under reduced pressure (up to 350 mbar). It was then filtered through silica (Et_2_O in hexanes, 10%) and concentrated under reduced pressure (up to 350 mbar) to afford **S6** as a colorless oil (isolated yield not determined).

*R*_f_ (Et_2_O in hexanes, 10%): 0.95; ^1^H NMR (400 MHz, C_6_D_6_) δ = 5.32–5.38 (m, 2H, C4–H, C8–H), 5.29 (tsxt, *J* = 8.0, 1.4 Hz, 1H, C13–H), 3.75 (d, *J* = 8.0 Hz, 2H, C14–H), 3.29 (d, *J* = 7.7 Hz, 2H, C5–H), 1.96–2.01 (m, 2H, C10–H), 1.79–1.84 (m, 5H, C9–H, C7–H), 1.56 (s, 3H, C1–H), 1.50 (s, 3H, C3–H), 1.35 (s, 3H, C12–H) ppm; ^13^C NMR (101 MHz, C_6_D_6_) δ = 141.8 (C11), 135.4 (C2), 131.2 (C6), 125.0 (C4), 121.3 (C13), 120.3 (C8), 40.8 (C14), 39.3 (C9), 30.0 (C5), 27.2 (C10), 25.7 (C1), 18.2 (C7), 17.7 (C3), 15.8 (C12) ppm.

#### (2*E*,6*E*)-3-Methyl-7-((3-methylbut-2-en-1-yl)­thio)­octa-2,6-dien-1-yl Trihydrogen Diphosphate, Triammonia Salt (**12**)

(*n*Bu_4_N)_3_HP_2_O_7_·3H_2_O (631 mg, 0.700 mmol, 1.75 equiv) was dissolved in MeCN (8.00 mL); 3Å MS was added; and the mixture was cooled to 0 °C. Allyl chloride **S6** (158 mg, 0.40 mmol, 1.00 equiv) was added, and the reaction mixture was stirred for 16 h at rt. The solvent was removed under reduced pressure. The residue was taken up in an ion-exchange buffer [1.00 mL, 25.0 mM NH_4_HCO_3_ in iPrOH/H_2_O (2.00%, v/v)] and converted into the ammonium salt using an ion-exchange column (DOWEX AG 50W-X8 (100–200 mesh), NH_4_^+^ form). After removal of the solvent under reduced pressure, the crude product was dissolved in aqueous NH_4_HCO_3_ solution (0.050 M, 2.00 mL). A solution of MeCN and iPrOH (1:1, 10.0 mL) was added, and the mixture was centrifuged for 5 min at 5000*g*. The supernatant was decanted and the residue redissolved in aqueous NH_4_HCO_3_ solution (0.050 M, 2.00 mL), before addition of MeCN in iPrOH (1:1, 10.0 mL). It was then centrifuged again for 5 min at 5000*g*, before the supernatant was decanted. The combined supernatants were concentrated under reduced pressure and dried freeze-drying to afford **12** (90.1 mg, 20.0 mmol, 50.0% o2s) as a yellow amorphous solid.

^1^H NMR (400 MHz, D_2_O) δ = 5.42–5.49 (m, 2H, C4–H, C8–H), 5.24 (tsxt, *J* = 7.7, 1.3 Hz, 1H, C13–H), 4.44 (t, *J* = 6.6 Hz, 2H, C14–H), 3.35 (d, *J* = 7.7 Hz, 2H, C5–H), 2.20–2.26 (m, 2H, C9–H), 2.08–2.12 (m, 2H, C10–H), 1.86 (s, 3H, C7–H), 1.70 (s, 6H, C1–H, C3–H), 1.65 (s, 3H, C12–H) ppm; ^13^C NMR (101 MHz, D_2_O) δ = 142.2 (C6, C11), 137.5 (C2), 128.4 (C4), 120.2 (C8), 118.9 (C13), 62.4 (C14), 38.3 (C9), 29.0 (C5), 26.6 (C10), 24.8 (C1/C3), 17.3 (C7), 17.0 (C12), 15.6 (C1/C3) ppm; ^31^P NMR (162 MHz, D_2_O) δ = −6.38 (d, *J* = 21.0 Hz), −9.96 (d, *J* = 22.4 Hz) ppm.

### Biotransformations, Isolation, and Structure Elucidations of **23**, **24**, and **27**

#### δ-3-Thiocadinene (**23**)

Procedure 1: δ-3-Thiocadinene **23** was isolated as a colorless oil. The biotransformation was carried out according to the general procedure described for the semipreparative biotransformation by using the sesquiterpene synthase Omp7 and FPP derivative **12** as substrate. Procedure 2: δ-3-Thiocadinene **23** was isolated as a colorless oil. The biotransformation was carried out according to the general procedure described for the semipreparative biotransformation by using the sesquiterpene synthase Cop4 and FPP derivative **12** as substrate at pH 7.6. A second major product formed too. Procedure 3: δ-3-Thiocadinene **23** was isolated as a colorless oil. The biotransformation was carried out according to the general procedure described for the semipreparative biotransformation by using the sesquiterpene synthase Cop4 and FPP derivative **12** as substrate at pH 8.

*R*_f_ (*n*-pentane 100%): 0.35; RI: 1759; ^1^H NMR: (500 MHz, C_6_D_6_) δ = 5.35 (s, 1H), 2.67 (d, *J* = 9.4 Hz, 1H), 2.65–2.59 (m, 1H), 2.48–2.39 (m, 2H), 2.02–1.91 (m, 2H), 1.84 (m, 5H), 1.62 (s, 3H), 1.60–1.46 (m, 1H), 0.80 (d, *J* = 6.8 Hz, 3H), 0.64 (d, *J* = 6.9 Hz, 3H,) ppm; ^13^C NMR: (101 MHz, C_6_D_6_) δ = 135.1, 127.1, 125.1, 118.8, 46.8, 39.5, 32.7, 28.7, 27.8, 26.2, 23.6, 21.5, 19.3, 16.1 ppm; HRMS [ESI] *m*/*z* calculated C_14_H_23_S^+^ [M + H]^+^: 223.1515, found: 223.1588; HRMS [ESI] *m*/*z* 245.1330 [M + Na]^+^ (calculated for C_14_H_22_NaS^+^ [M + Na]^+^: 245.1334).

The following 2D NMR techniques were utilized for structure elucidation: COSY, HSQC, HMBC, and NOESY. Assignments of the ^1^H and ^13^C signals are given in the Supporting Information. The structure elucidation was further supported by an authentic sample of δ-cadinene **25**, which served for comparison reasons.

Manual olfactory analyses: stale/grumpy and no signs of cannabis or rotten eggs.

#### 3-Thiocadinol (**24**)

3-Thiocadinol **24** was isolated as a colorless oil. The biotransformation was carried out according to the general procedure described for the semipreparative biotransformation by using the sesquiterpene synthase Cop4 and FPP derivative **12** as substrate at pH 7.6.

*R*_f_ (10% Et_2_O in *n*-pentane): 0.50; ^1^H NMR: (400 MHz, C_6_D_6_) δ = 5.60 (dsxt, *J* = 6.0, 1.5 Hz, 1H), 2.81 (t, *J* = 12.3 Hz, 1H), 2.68–2.74 (m, 1H), 2.20 (dd, *J* = 13.1, 2.9 Hz, 1H), 1.96–2.11 (m, 2H), 1.83–1.90 (m, 3H), 1.72 (tt, *J* = 11.6, 3.1 Hz, 1H), 1.59 (p, *J* = 1.1 Hz, 3H), 1.45–1.51 (m, 1H), 1.32–1.34 (m, 4H), 0.78 (dd, 7.0, 4.3 Hz, 6H) ppm; ^13^C NMR: (101 MHz, C_6_D_6_) δ = 134.1, 125.5, 80.2, 46.3, 44.7, 34.6, 31.2, 30.3, 27.4, 24.5, 23.7, 21.6, 21.5, 15.5 ppm; HRMS [ESI] *m*/*z* 263.1437 [M + Na]^+^ (calculated for C_14_H_24_NaOS^+^ [M + Na]^+^: 263.1440).

The following 2D NMR techniques were utilized for structure elucidation: COSY, HSQC, HMBC, and NOESY. Assignments of the ^1^H and ^13^C signals are given in the Supporting Information. The structure elucidation was further supported by an authentic sample of δ-cadinol **26**, which served for comparison reasons.

Manual olfactory analyses: stale/grumpy and no signs of cannabis or rotten eggs.

#### Ketothiol (**27**)

Ketothiol **27** was isolated as a colorless oil. The biotransformation was carried out according to the general procedure described above by using STS BcBOT2 and FPP derivative **12** as substrate at pH 7.6.

*R*_f_ (10% Et_2_O in *n*-pentane): 0.38. ^1^H NMR: (600 MHz, C_6_D_6_) δ = 2.78 (td, *J* = 6.8, 3.0 Hz, 1H), 2.39 (ddd, *J* = 11.7, 6.9, 3.0 Hz, 1H), 2.30 (dd, *J* = 10.2, 3.0 Hz, 1H), 2.08–2.16 (m, 1H), 1.89 (s, 3H), 1.64 (q, *J* = 6.9 Hz, 2H), 1.24–1.42 (m, 4H), 1.16 (s, 3H), 1.10–1.16 (m, 1H), 0.87 (s, 3H), 0.69 (s, 3H) ppm; ^13^C NMR: (151 MHz, C_6_D_6_) δ = 208.7, 60.4, 60.2, 58.5, 57.6, 48.6, 44.2, 43.2, 30.4, 29.7, 29.6, 28.8, 25.4, 23.4 ppm; HRMS [ESI] *m*/*z* 263.1435 [M + Na]^+^ (calculated for C_14_H_24_NaOS^+^ [M + Na]^+^: 263.1440).

The following 2D NMR techniques were utilized for structure elucidation: COSY, HSQC, HMBC, and NOESY. Assignments of the ^1^H and ^13^C signals are found in the Supporting Information.

Manual olfactory analyses: unpleasant and spicy.

## Supplementary Material



## Notes

Detailed chemical procedures, biotransformations, and spectral data as well as copies of NMR spectra (PDF)
