# Supplementary material for: Chemoenzymatic Generation of Thio-analogues of δ‑Cadinene, δ‑Cadinol, and a Thio-diquinane Using 8‑Thio-farnesylpyrophosphate
Source: J Nat Prod. 2025 Jul 17;88(7):1653–62. doi: 10.1021/acs.jnatprod.5c00409 (PMC12305643; doi:10.1021/acs.jnatprod.5c00409)

S1UPPORTING INFORMATION

Chemoenzymatic generation of thio-analogues of δ-cadinene, δ-cadinol and a thio-diquinane using 8-thio-farnesylpyrophosphate

Birk Jäger[a], Jan Luca Budde[a], Norman Birke[a], Maximilian Hauke[a], and Andreas Kirschning*[a,b]

[a] Institute of Organic Chemistry, Leibniz University Hannover, Schneiderberg 1B, 30167 Hannover, Germany
[b] Uppsala Biomedical Center (BMC), Uppsala University, Husargatan 3, 752 37 Uppsala, Sweden
email address: andreas.kirschning@oci.uni-hannover.de

----

1. Experimental procedures **1**

1.1. General information 1

1.2. Protein expression, growth conditions and analytical assays 2

2. Chemical synthesis of FPP derivative 12 **5**

3. Biotransformations, isolation and structure elucidations of new terpenoids **12**

4. References (supporting information) **25**

5. Copies of NMR spectra **26**

6. GC-MS data of the new terpenoids **41**

1. Experimental Procedures

1.1. General information

General: All experiments were performed using an inert argon atmosphere and dry solvents unless otherwise specified. Glassware was dried with a heat gun before use.

Reagents and solvents: All commercially available solvents and reagents were used as received (ACROS, ABCR, SIGMA-ALDRICH, TCI, ALFA). THF was freshly distilled from Na/benzophenone. Deuterated solvents for NMR were acquired from DEUTERO.

Flash Column Chromatography: The silica gel used for manual flash column chromatography was acquired from MACHERY-NAGEL (type 60 M, grain size 40 µm to 63 µm). Automated flash column chromatography was conducted with the flash purification system SEPACORE® by BÜCHI using prepacked cartridges (PuriFlash® by INTERCHIM or Chromabond® by MACHERY-NAGEL). The eluents are given in parentheses.

Thin Layer Chromatography (TLC): For TLC, aluminum plates coated with silica gel, type 60 F_{254} by MERCK, were used and the spots were visualized with UV light (l = 248 nm) or alternatively by staining with anisaldehyde, vanillin or potassium permanganate solutions.

Ion Exchange Chromatography: Ion exchange chromatography was performed using the Amberchrom® 50WX8 (H^+-form, 100 – 200 mesh) which can be reused up to ten times. 100 g of the material were taken up in water and washed with excess of 6 % aq. NH_3, until a pH of 14 was reached. It was then washed with distilled water until pH = 7 was reached. Then it was equilibrated with IEB (aq. 25 mM NH_4HCO_3 with 2 % *i*PrOH, ~100 mL). When the purification was finished, the column was transformed back to its H^+-form by first washing it with an excess of 3 M HCl until pH = 1 and then washing it with an excess *of distilled water until pH = 7 was reached.*

NMR-Spectroscopy: ^{1}H-NMR, ^{13}C-NMR and ^{31}P-NMR spectra were recorded with the DPX-400 (400 MHz), AVS-400 (400 MHz) and the Ultrashield 500 (500 MHz) by BRUKER at 298 K. ^{1}H and ^{13}C positive chemical shifts (δ) are referenced to $CHCl_3$ (^{1}H: 7.26 ppm, ^{13}C: 77.2 ppm), D_2O (^{1}H: 4.79 ppm) or C_6D_6 (^{1}H: 7.16 ppm, ^{13}C: 128.06 ppm) and are given in parts per million (ppm). Coupling constants (*J*) are given in Hertz (Hz) and reported as observed. The ^{1}H-NMR data are reported as follows: chemical shift (multiplicity, coupling constants, integral, assignment). The ^{13}C-NMR data are reported as follows: chemical shift (assignment). The ^{31}P-NMR data are reported as follows: chemical shift (multiplicity, coupling constants). NMR assignments are made according to spin systems, using two-dimensional (COSY, HSQC, HMBC) NMR spectroscopy to assist the assignment. The multiplicities are reported as follows: s: singlet, d: doublet, t: triplet, q: quartet, p: pentet, sxt: sextet, spt: septet, m: multiplet, br: broad signal.

Gas Chromatography: GC/MS analyses were carried out with an Agilent 7890B GC with 5977B GC/MSD and Gerstel MPS Robotic XL with KAS 4C injector. Samples were analysed on an Optima 5HT column, 30 m x 250 µm i.d. x film thickness 0.25 µm). Carrier gas, He; injector temp.: 60 °C to 300 °C at 12°C/min, splitless or split ratio 1:40; temp. program: 50 °C (isothermal 1 min) to 300 °C, at 20 °C/min and held isothermal for 6.5 min at 300°C; FID: 300°C, H2: 30 mL/min, N2: 25 mL/min, MSD: ion source: EI 70 eV, 230 °C; detector: quadrupole, EI mass spectra were acquired over the mass range of 30 –650 amu. Further GC/MS analyses were carried out with an Agilent GC 7890B chromatograph with Gerstel CIS4 Cold Injector.

Mass Spectrometry: High resolution mass spectra (HRMS) were recorded with a MICROMASS LCT with a lockspray dual ion source in combination with a WATERS Alliance 2695 system. Injection was conducted in loop mode. Alternatively, a QTOF premier spectrometer (WATERS) in combination with a WATERS Acquity UPLC system was used. Ionisation was carried out via electrospray-ionisation (ESI). The calculated and the detected masses are reported. For analytical enzyme tests, an injection volume of 1 µL *splitless* was used.

Freeze-drying system: The lyophilisation of the diphosphate compounds shown was carried out on the CHRIST ALPHA 2-4 LDC-1M freeze-drying system at a temperature of –18 °C to –25 °C and a pressure of 0.250 mbar.

Naming of Compounds: Compound names are those generated by ChemBioDraw 22.2.0 software (PERKINELMER), following IUPAC nomenclature.

1.2. Protein expression, growth conditions and analytical assays

Heterologous expression in Escherichia coli: Heterogeneous expression of STCs was conducted in Escherichia coli BL21 (DE3). For the main culture, 50 – 100 mL of 2TY medium were inoculated with 20 % of a preculture. The latter was previously incubated for 16 hours at 37 °C and 200 rpm. The main culture was also cultivated at 37 °C and 200 rpm until exponential growth of the cell culture prevailed, reaching an OD_{600} of 0.5 – 0.8. Induction was then performed with 0.5 mM isopropyl β-d-1-

thiogalactopyranoside (IPTG). The primary culture was then incubated at 16 °C and 180 rpm for approximately 22 hours and followed by centrifugation for 10 min at 5000 g. The primary culture was then incubated at 16 °C and 180 – 200 rpm for approximately 22 hours and followed by centrifugation for 10 min at 5000 *g* and cell disruption was performed thereafter. Cell pellets not required initially were stored at –20 °C until further use.

To check the expression, samples of 1 mL each were taken from the main culture before induction with IPTG and after 22 hours of cultivation. The samples were centrifuged for 10 min at 5000 g. The pellet was prepared directly for use in an SDS-PAGE and stored at –20 °C.

Discontinuous SDS–polyacrylamide gel electrophoresis (SDS-PAGE): An SDS-PAGE experiment was conducted to separate proteins based on their size. To accomplish this, a 5 % collection gel and a 15 % separation gel were utilised (refer to Table). The distinct pH values and buffer strengths of the gels lead to a concentration of protein bands at the boundary of the separation gel, thereby enhancing the separation of bands.

Table S1: Composition of the collection- and separation gel.

Component	Separation gel (15 %, 10 mL)	Collection gel (5 %, 5 mL)
30 % acryl-bisacryl-mix	5 mL	0.83 mL
1.5 M TRIS-base	2.5 mL (pH 8.8)	0.63 mL (pH 6.8)
SDS	0.1 %	0.05 %
APS	0.1 %	0.05 %
TEMED	4 µL	5 µL

First, the gel electrophoresis protocol involved pouring the separating gel between two fixed glass discs, which were then coated with the collecting gel upon polymerisation. Once the collection gel had also polymerised, the gel could be placed in the vertical electrophoresis chamber, which was filled with 1x SDS running buffer. The protein samples were mixed with 100 µL Lämmli buffer and heated to 95 °C for 10 min for denaturation. Cell samples were also mixed with 100 µL Lämmli buffer and heated to 95 °C for 30 min for denaturation. As the size standard, *Colour Prestained Protein Standard* from New England Biolabs, Inc, was used. Electrophoresis was initially conducted at 100 V for 20 min., followed by 150 V for 100 min. Subsequently, the gel was treated with Coomassie staining solution for 16 h, followed by 4 h of decolorisation.

Ultrasonic cell disruption: For cell disruption by ultrasound, the cell pellets were resuspended in 1 mL of lysis buffer per 100 mg pellet. Disruption was performed for 7 min on ice at an amplitude of 45 % with a 6 s pause per 10 s. This was followed by separation of the insoluble cell debris from the cell lysate by centrifugation for 20 min at 4 °C and 10,000 *g*.

Protein purification by immobilised-metal-ion affinity chromatography: The STCs dissolved in the cell lysate were purified using immobilised metal ion affinity chromatography (IMAC), as the proteins have a 6 x His tag due to their expression in pET28a(+), which binds to nickel-nitrilotriacetic acid (Ni-NTA). With increasing concentration of imidazole, the interaction was interrupted and the target proteins were eluted.

Concentrating protein solutions: To increase the concentration of proteins in a protein solution, it was concentrated to 0.2 – 0.5 mL using an Amicon Ultra-15 centrifugal filter from MERCK KGaA with an exclusion limit of 30 kDa and the filtrate was discarded.

UV/VIS-Spectroscopy for the determination of the protein concentration: To determine the enzyme concentration using UV/VIS spectroscopy, the absorbance of 1 µL purified protein solution was determined at 280 nm. The extinction coefficient of the protein without disulphide bridges stored in the device was used to determine the concentration.

Analytical in vitro enzyme tests: *In vitro* enzyme tests on a 500 µL scale were carried out to determine the protein activity and the acceptance and transformation of an allylic pyrophosphate. A HEPES or TRIS buffer was used for this purpose. The STC, at a concentration of 0.01 g/L and present in the corresponding buffer (1:1 HEPES-buffer/50 % glycol), was incubated with either FPP (**7**, 150 µM) or pyrophosphate **115** (150 µM) for 30 min at a temperature of 30 °C. Negative controls consisted of analogous preparations in the absence of pyrophosphate **115** or the STC. Afterward, extraction was performed by adding 100 µL of n-hexane, mixing for 30 s on the vortex mixer, and centrifuging for 5 min. at 3000 rpm and 4 °C. Subsequently, 50 µL of the organic phase were extracted, separated via gas chromatography, and analysed using GC-MS.

Centrifuging: The centrifuging was carried out on the THERMO SCIENTIFIC Heraeus Megafuge 16R Centrifuge for 5 min at a temperature of 4 °C and 3000 rpm.

Compositions of buffers and media: The compositions of the used buffers, media and stains are shown in Table .

Table S2: Composition of the buffers, media and stains used during this project.

Buffer/medium/stain	Composition
LB-medium	0.050 % yeast extract<br>1.00 % Tryptone<br>0.050 % NaCl
2TY-medium	1.00 % yeast extract<br>1.60 % Tryptone<br>0.500 % NaCl
TRIS buffer	40.0 mM TRIS-HCl<br>100 mM NaCl<br>pH 8.00
HEPES buffer	50.0 mM HEPES<br>5.00 mM DTT<br>pH 7.60
Preservation buffer	25.0 mM HEPES<br>2.50 mM DTT<br>25.0 % (v/v) glycerine<br>pH 7.60
Lämmli buffer	150 mM TRIS-HCl (pH 6.8)<br>6.00 % (m/v) SDS<br>30.0 % (v/v) glycerine<br>0.020 % (m/v) bromophenol blue
Coomassie stain	25.0 % (v/v) iPrOH<br>10.0 % (v/v) AcOH<br>0.100 % (m/v) Coomassie brilliant blue P250

2. Chemical synthesis of FPP derivative 12

3-Methylbut-2-ene-1-thiol (20)

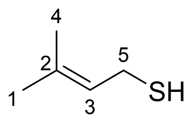

20
$C_5H_{10}S$
102,19 g/mol

To a solution of prenyl bromide (**19**) (4.47 g, 30.0 mmol, 1.00 eq.) in EtOH (24.0 mL) was added thiourea (2.28 g, 30.0 mmol, 1.00 eq.). The mixture was warmed to 80 °C and stirred for 3 h. NaOH (aq., 10.0 wt%, 24.0 mL) was added and the mixture was stirred for an additional 3 h at 70 °C. The reaction mixture was cooled to rt and diluted with Et_2O (25.0 mL). The phases were separated and HCl (aq., 1.00 M) was added to acidify the aqueous phase to pH = 2. It was then extracted with Et_2O (3 × 20.0 mL) and the combined organic phases were dried over $MgSO_4$ and filtered. The crude material was purified via distillation (80 – 100 °C at 1 bar) to afford **20** (1.43 g, 14.0 mmol, 46.8 %) as a colorless liquid (2.03 M solution in $EtOH/Et_2O$).

R$_f$ (EtOAc in hexanes, 12.5 %): 0.6.

^{1}H-NMR (400 MHz, $CDCl_3$) δ 5.28 (tsxt, *J* = 7.8, 1.2 Hz, 1H, C3-H), 3.10 (t, *J* = 7.5 Hz, 2H, C5-H), 1.67 (s, 3H, C4-H), 1.61 (s, 3H, C1-H) ppm.

^{13}C-NMR (101 MHz, $CDCl_3$) δ 134.1 (C2), 123.4 (C3), 25.6 (C4), 22.2 (C5), 17.4 (C1) ppm.

NMR spectroscopic data are in agreement with those reported in literature.[S1]

(*E*)-5-(3,3-Dimethyloxiran-2-yl)-3-methylpent-2-en-1-yl acetate (S1)

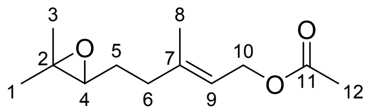

S1
$C_{12}H_{20}O_3$
212,29 g/mol

Geranyl acetate **13** (1.96 g, 10.0 mmol, 1.00 eq.) was dissolved in CH_2Cl_2 (13.0 mL) at rt. The mixture was then cooled to 0 °C and *m*CPBA (70 wt%, 2.71 g, 11.0 mmol, 1.10 eq.) was added slowly. The solution was stirred for 2.5 h at 0 °C and the reaction was terminated by addition of H_2O (15.0 mL). The phases were separated, and the aqueous phase was extracted with EtOAc (3 × 15.0 mL). Subsequently, the combined organic phases were washed with brine (60.0 mL), dried over $MgSO_4$, filtered and concentrated under reduced pressure. The residue was purified by flash column chromatography on silica gel (EtOAc in hexanes, 17 %) affording **S1** (1.72 g, 8.09 mmol, 80.9 %) as a colorless oil.

R$_f$ (EtOAc in hexanes, 17 %): 0.6.

^{1}H-NMR (400 MHz, $CDCl_3$) δ 5.38 (tsxt, *J* = 7.1, 1.3 Hz, 1H, C9-H), 4.99 (d, *J* = 7.1 Hz, 2H, C10-H), 2.70 (t, *J* = 6.2 Hz, 1H, C4-H), 2.11 – 2.26 (m, 2H, C6-H), 2.05 (s, 3H, C12-H), 1.72 (s, 3H, C8-H), 1.63 – 1.69 (m, 2H, C5-H), 1.30 (s, 3H, C1-H/C3-H), 1.26 (s, 3H, C1-H/C3-H) ppm.

^{13}C-NMR (101 MHz, $CDCl_3$) δ 171.2 (C11), 141.4 (C7), 119.0 (C9), 64.0 (C4), 61.4 (C10), 58.5 (C2), 36.3 (C6), 27.2 (C5), 25.0 (C1/C3), 21.2 (C12), 18.9 (C1/C3), 16.6 (C8) ppm.

Analytical data are in agreement with those reported in literature.[S2]

(*E*)-3-Methyl-6-oxohex-2-en-1-yl acetate (S2)

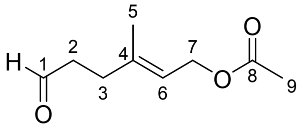

151
$C_9H_{14}O_3$
170,21 g/mol

Periodic acid (2.21 g, 9.71 mmol, 1.20 eq.) was dissolved in THF (72.8 mL) and cooled to 0 °C. Epoxide **S1** (1.72 g, 8.09 mmol, 1.00 eq.) in Et_2O (12.1 mL) was then added and the mixture was stirred for 1.5 h. The reaction mixture was diluted with Et_2O (50.0 mL) and the phases were separated. The organic phase was washed with sat. aq. $NaHCO_3$ (100 mL), brine (100 mL), dried over $MgSO_4$, filtered and concentrated under reduced pressure to afford **S2** (isolated yield not determined) as a light yellow oil. It was used for the next step without further purification.

R_f (EtOAc in hexanes, 10 %): 0.8.

^{1}H-NMR (400 MHz, $CDCl_3$) δ 9.77 (s, 1H, C1-H), 5.36 (tsxt, J = 7.1, 1.3 Hz, 1H, C6-H), 4.57 (d, J = 7.1 Hz, 2H, C7-H), 2.55 – 2.60 (m, 2H, C3-H), 2.35 – 2.40 (m, 2H, C2-H), 2.05 (s, 3H, C9-H), 1.72 (s, 3H, C5-H) ppm.

^{13}C-NMR (101 MHz, $CDCl_3$) δ 201.8 (C1), 171.2 (C8), 140.1 (C4), 119.5 (C6), 61.2 (C7), 41.9 (C2), 32.0 (C3), 21.2 (C9), 16.7 (C5) ppm.

NMR spectroscopic data are in agreement with those reported in literature.[S2]

(*E*)-7,7-Dibromo-3-methylhepta-2,6-dien-1-yl acetate (S3)

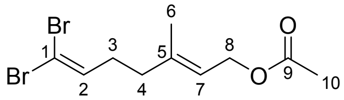

S3
$C_{10}H_{14}Br_2O_2$
326,03 g/mol

A solution of PPh_3 (36.7 g, 140 mmol, 2.80 eq.) in CH_2Cl_2 (250 mL) was cooled to 0 °C, before CBr_4 (23.2 g, 70.0 mmol, 1.40 eq.) was added. The mixture was stirred for 30 min. at 0 °C and for additional 30 min. at rt. Then, the mixture was cooled to 0 °C, before a solution of **S2** (8.51 g, 50.0 mmol, 1.00 eq.) in CH_2Cl_2 (25.0 mL) was added. The reaction mixture was stirred for 1.5 h, before being diluted using PE (500 mL). The mixture was filtered through a pad of Celite™. The residue was dissolved in CH_2Cl_2 (30.0 mL), before PE (300 mL) was added. It was again filtered through a pad of Celite™. This was repeated two additional times, before the filtrate was concentrated under reduced pressure and filtered through silica, affording **S3** (isolated yield not determined) as a yellow oil.

R_f (EtOAc in hexanes, 10 %): 0.8.

^{1}H-NMR (400 MHz, $CDCl_3$) δ 6.35 (t, J = 6.9 Hz, 1H, C2-H), 5.36 (tsxt, J = 7.1, 1.3 Hz, 1H, C7-H), 4.59 (d, J = 7.1 Hz, 2H, C8-H), 2.21 – 2.27 (m, 2H, C4-H), 2.12 – 2.16 (m, 2H, C3-H), 2.06 (s, 3H, C10-H), 1.71 (s, 3H, C6-H) ppm.

^{13}C-NMR (101 MHz, $CDCl_3$) δ 171.2 (C9), 140.5 (C5), 137.8 (C1), 119.8 (C7), 89.4 (C2), 61.3 (C8), 37.3 (C3), 31.2 (C4), 21.2 (C10), 16.5 (C6) ppm.

NMR spectroscopic data are in agreement with those reported in literature.[S2]

(*E*)-7,7-Dibromo-3-methylhepta-2,6-dien-1-ol (14)

Acetate **S3** (13.2 g, 40.5 mmol, 1.00 eq.) was dissolved in MeOH (243 mL) at rt. No precautions were taken to ensure dry or inert

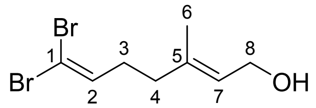

14
$C_8H_{12}Br_2O$
283,99 g/mol

conditions. K_2CO_3 (6.16 g, 44.6 mmol, 1.10 eq.) was added and the mixture was stirred for 30 min. at rt. CH_2Cl_2 (250 mL) was added and the phases were separated. The aqueous phase was extracted with CH_2Cl_2 (3 × 100 mL) and the combined organic phases were washed with brine (400 mL), dried over $MgSO_4$, filtered and concentrated under reduced pressure to afford **14** (10.1 g,

35.6 mmol, 88.0 % o4s) as a colorless oil. It could be employed in the next step without further purification.

R$_f$ (EtOAc in hexanes, 10 %): 0.02.

^{1}H-NMR (400 MHz, $CDCl_3$) *δ* 6.37 (t, *J* = 6.8 Hz, 1H, C2-H), 5.43 (tsxt, *J* = 6.9, 1.3 Hz, 1H, C7-H), 4.17 (d, *J* = 6.7 Hz, 2H, C8-H), 2.21 – 2.27 (m, *J* = 2H, C4-H), 2.11 – 2.15 (m, 2H, C3-H), 1.69 (m, 3H, C6-H), 1.58 (brs, 1H, C8-OH) ppm.

^{13}C-NMR (101 MHz, $CDCl_3$) *δ* 138.0 (C1), 124.7 (C5), 89.2 (C2), 59.5 (C8), 37.4 (C3), 31.3 (C4), 16.3 (C6) ppm.

NMR spectroscopic data are in agreement with those reported in literature.[S2]

(E)-tert-Butyl((7,7-dibromo-3-methylhepta-2,6-dien-1-yl)oxy)diphenylsilane (15)

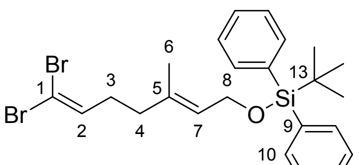

15
$C_{24}H_{30}Br_2OSi$
522,40 g/mol

Alcohol **14** (10.1 g, 35.6 mmol, 1.00 eq.) was dissolved in DMF (35.6 mL). Imidazole (5.34 g, 78.4 mmol, 2.20 eq.) was added and the mixture was stirred for 10 min. at rt, before being cooled down to 0 °C. TBDPSCl (8.05 g, 53.4 mmol, 1.50 eq.) was added and the mixture was stirred for 16 h and the reaction was terminated by addition of H_2O (35.0 mL). The phases were separated, and the aqueous phase was extracted with CH_2Cl_2 (3 × 35.0 mL). The combined organic phases were washed with brine (125 mL), dried over $MgSO_4$, filtered and concentrated under reduced pressure. The residue was purified by flash column chromatography on silica gel (EtOAc in hexanes, 5 %) affording **15** (17.5 g, 33.5 mmol, 94.2 %) as a colourless oil.

Rf (EtOAc in hexanes, 10 %): 0.9.

^{1}H-NMR (400 MHz, CDCl3) δ 7.68 – 7.70 (m, 4H, C10-H), 7.36 – 7.42 (m, 6H, C11-H, C12-H), 6.35 (t, J = 7.0 Hz, 1H, C2-H), 5.39 (tsxt, J = 6.3, 1.2 Hz, 1H, C7-H), 4.23 (dsxt, J = 6.2, 0.7 Hz, 2H, C8-H), 2.15 – 2.22 (m, 2H, C4-H), 2.05 – 2.10 (m, 2H, C3-H), 1.44 (s, 3H, C6-H), 1.05 (s, 9H, C13-$(CH_3)_3$) ppm.

^{13}C-NMR (101 MHz, CDCl3) δ 138.2 (C5), 135.8 (C2), 135.4 (C9), 134.1 (C12), 129.7 (C10/C11), 127.8 (C10/C11), 125.5 (C7), 89.0 (C1), 61.2 (C8), 37.3 (C4), 31.3 (C3), 27.0 (C13-$(CH_3)_3$), 19.3 (C13), 16.3 (C6) ppm.

NMR spectroscopic data are in agreement with those reported in literature.[S2]

(*E*)-*tert*-Butyl((3-methylhept-2-en-6-yn-1-yl)oxy)diphenylsilane (S4):

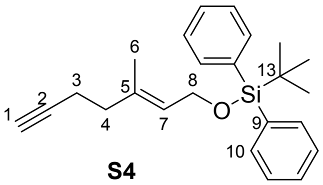

S4
$C_{24}H_{30}OSi$
362,59 g/mol

Dibromoalkene **15** (2.29 g, 4.39 mmol, 1.00 eq.) was dissolved in Et_2O (50.0 mL) and the solution was cooled to 0 °C. *n*BuLi (2.0 M in hexane, 4.83 mL, 9.66 mmol, 2.20 eq.) was slowly added and the mixture was stirred for 1 h at 0 °C. The reaction was terminated by the addition of H_2O (50.0 mL). The phases were separated, and the aqueous phase was extracted with Et_2O (3 × 50.0 mL). The combined organic phases were washed with brine (200 mL), dried over $MgSO_4$, filtered and concentrated under reduced pressure. The residue was purified by flash column chromatography on silica gel (CH_2Cl_2 in hexanes, 20 %) affording **S4** as a light yellow oil (1.32 g, 3.51 mmol, 80.0 %).

R$_f$ (CH_2Cl_2 in hexanes, 20 %): 0.51.

^{1}H-NMR (400 MHz, $CDCl_3$) *δ* 7.68 – 7.71 (m, 4H, C10-H), 7.36 – 7.42 (m, 6H, C11-H, C12-H), 5.43 (tsxt, *J* = 6.3 Hz, 1.2 Hz, 1H, C7-H), 4.23 (d, *J* = 6.3 Hz, 2H, C8-H), 2.24 – 2.29 (m, 2H, C4-H), 2.17 – 2.22 (m, 2H, C3-H), 1.94 (t, *J* = 2.6 Hz, 1H, C1-H), 1.44 (s, 3H, C6-H), 1.04 (s, 9H, C13-$(CH_3)_3$) ppm.

^{13}C-NMR (101 MHz, $CDCl_3$) δ 135.8 (C5), 135.2 (C9), 134.1 (C12) 129.7 (C10/C11), 127.7 (C10/C11), 125.5 (C7), 84.2 (C2), 68.6 (C1), 61.1 (C8), 38.3 (C4), 27.0 (C13-$(CH_3)_3$), 19.3 (C13), 17.4 (C3), 16.6 (C6) ppm.

HRMS [ESI] *m/z* calculated $C_{24}H_{30}OSiNa^+$ $[M+H]^+$: 363.2144, found: 363.2127.

(*E*)-*tert*-Butyl((3-methyloct-2-en-6-yn-1-yl)oxy)diphenylsilane (16)

Procedure 1: S4 (169 mg, 0.320 mmol, 1.00 eq.) was dissolved in THF (0.809 mL) and the solution was cooled to 0 °C. *n*BuLi (2.15 M in hexane, 0.380 mL, 0.810 mmol, 2.50 eq.) was slowly added and the mixture was stirred for 1.5 h at rt. Then, the mixture was cooled back down to 0 °C before slow addition of a second portion of *n*BuLi (2.15 M in hexane, 0.170 mL, 0.360 mmol, 1.10 eq.). It was then stirred for 45 min. at 0 °C, before addition of MeI (459 mg, 0.200 mL, 3.24 mmol, 10.0 eq.) and stirring was continued for an additional 45 min. at 0 °C. Then it was warmed to rt while stirring for 3 h. The reaction was then terminated by addition of aq. sat. NH_4Cl (1.00 mL). The phases were separated, and the aqueous phase was extracted with Et_2O (3 × 1.00 mL). The combined organic phases were washed with brine (5.00 mL), dried over $MgSO_4$, filtered and concentrated under reduced pressure. The residue was purified by flash column chromatography on silica gel (CH_2Cl_2 in hexanes, 20 %) affording **16** (43.6 mg, 0.116 mmol, 35.8 %) as a light yellow oil.

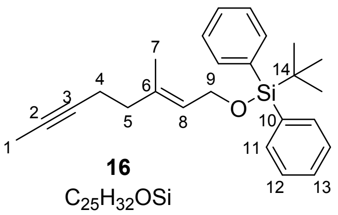

16
$C_{25}H_{32}OSi$
376,61 g/mol

Procedure 2: S4 (1.11 g, 3.05 mmol, 1.00 eq.) was dissolved in THF (9 mL) and the solution was cooled to –78 °C. *n*BuLi (2.00 M in hexanes, 1.68 mL, 3.35 mmol, 1.10 eq.) was added and the mixture was stirred for 20 min. at – 78 °C. After addition of MeI (4.33 g, 1.90 mL, 30.5 mmol, 10.0 eq.), the mixture was slowly warmed to rt and stirring was continued for 1 h. The reaction was terminated by addition of H_2O (10.0 mL). The phases were separated, and the aqueous phase was extracted with Et_2O (3 × 10.0 mL). The combined organic phases were washed with brine (50.0 mL), dried over $MgSO_4$, filtered and concentrated under reduced pressure. The residue was purified by flash column chromatography on silica gel (CH_2Cl_2 in hexanes, 20 %) affording **16** (996 mg, 2.65 mmol, 71.4 %) as a light yellow oil.

R_f (CH_2Cl_2 in hexanes, 20 %): 0.51.

^{1}H-NMR (400 MHz, $CDCl_3$) δ 7.68 – 7.70 (m, 4H, C11-H), 7.35 – 7.44 (m, 6H, C12-H, C13-H), 5.42 (tsxt, J = 6.3 Hz, 1.2 Hz, 1H, C8-H), 4.22 (d, J = 6.4 Hz, 2H, C9-H), 2.18 – 2.23 (m, 2H, C5-H), 2.13 – 2.16 (m, 2H, C4-H), 1.76 (t, J = 2.4 Hz, 3H, C1-H), 1.43 (s, 3H, C7-H), 1.04 (s, 9H, C14-$(CH_3)_3$) ppm.

^{13}C-NMR (101 MHz, $CDCl_3$) δ 135.8 (C11), 134.2 (C6), 129.7 (C10), 127.7 (C12, C13), 125.1 (C8), 79.0 (C3), 75.9 (C2), 61.2 (C9), 39.0 (C5), 26.7 (C14-$(CH_3)_3$), 19.3 (C14), 17.8 (C4), 16.3 (C7), 3.6 (C1) ppm.

The spectroscopic and analytical data are in accordance with those reported in the literature.[S3]

***tert*-Butyl-(((2*E*,6*E*)-7-iodo-3-methylocta-2,6-dien-1-yl)oxy)diphenylsilane (17)**

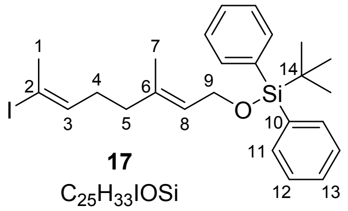

17
$C_{25}H_{33}IOSi$
504,53 g/mol

Zirconocene dichloride (4.26 g, 14.6 mmol, 2.75 eq.) was dissolved in THF (29.2 mL) and the solution was cooled to 0 °C. DIBAL-H (1.00 M in hexanes, 14.6 mL, 14.6 mmol, 2.75 eq.) was added and the mixture was stirred for 1 h at 0 °C. A solution of alkyne **16** (2.00 g, 5.28 mmol, 1.00 eq.) in THF (2.64 mL) was added and the reaction mixture was stirred for 16 h at rt. Next iodine (2.68 g, 10.6 mmol, 2.00 eq.) was added and the mixture was stirred for 15 min. at rt, before the reaction was terminated by addition of 1.00 M aq. HCl (15.0 mL). The phases were separated and the aqueous phase was extracted with Et_2O (3 × 15.0 mL). The combined organic phases were washed with sat. aq. $Na_2S_2O_3$ (60.0 mL), brine (60.0 mL), dried over $MgSO_4$, filtered and concentrated under reduced pressure. It was then purified by flash column chromatography (CH_2Cl_2 in hexanes, 12 %) to afford the vinyliodide (d.r. 6:1). The diastereoisomers were separated by flash column chromatography (CH_2Cl_2 in hexanes, 6.3 %) to afford the desired isomer **17** (1.25 g, 2.38 mmol, 44.7 %, d.r. 12:1) as a colorless oil.

$\mathbf{R_f}$ (CH_2Cl_2 in hexanes, 12 %): 0.5.

^{1}H-NMR (400 MHz, $CDCl_3$) δ 7.71 – 7.73 (m, 4H, C11-H), 7.38 – 7.45 (m, 6H, C-12H, C13-H), 6.15 (tsxt, J = 7.3, 1.5 Hz, 1H, C3-H), 5.40 (tsxt, J = 6.3, 1.2 Hz, 1H, C8-H), 6.24 (d, J = 6.3 Hz, 2H, C9-H), 2.39 (m, 3H, C1-H), 2.11 – 2.16 (m, 2H, C5-H), 2.01 – 2.06 (m, 2H, C4-H), 1.45 (s, 3H, C7-H), 1.07 (s, 9H, C14-$(CH_3)_3$) ppm.

^{13}C-NMR (101 MHz, $CDCl_3$) δ 140.8 (C9), 136.7 (C11), 134.2 (C13), 129.7 (C10), 127.8 (C12), 125.0 (C8), 93.8 (C2), 61.2 (C9), 53.6 (C6), 38.5 (C4), 27.7 (C1), 29.1 (C5), 27.0 (C14-$(CH_3)_3$), 19.3 (C14), 16.4 (C7) ppm.

Analytical data are in agreement with those reported in literature.[S4]

***tert*-Butyl-(((2*E*,6*E*)-3-methyl-7-((3-methylbut-2-en-1-yl)thio)octa-2,6-dien-1-yl)oxy)diphen-ylsilane S5) and (6*E*,10*E*)-2,2,7,11,15,15,19-Heptamethyl-3,3-diphenyl-4-oxa-12,16-dithia-3-silaicosa-6,10,18-triene (22)**

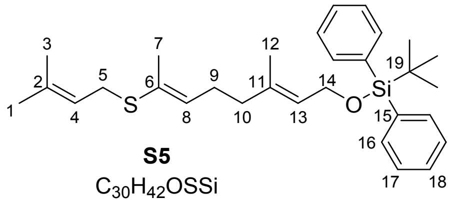

S5
$C_{30}H_{42}OSSi$
478,81 g/mol

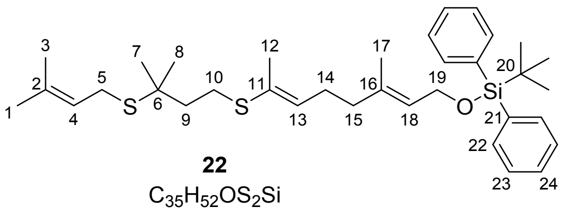

22
$C_{35}H_{52}OS_2Si$
581,01 g/mol

Vinyliodide **17** (1.20 g, 2.38 mmol, 1.0 eq.) and thiol **20** (267 mg, 2.61 mmol, 1.10 eq.) were dissolved in toluene (10.5 mL) and degassed under a stream of argon for 15 min. Then, $[Cu(phen)(PPh_3)_2]NO_3$ (**21**, 651 mg, 0.714 mmol, 30.0 mol%) and K_3PO_4 (908 mg, 4.28 mmol, 1.80 eq.) were added and the reaction mixture was stirred for 16 h at 80 °C, before the reaction was terminated by addition of H_2O (10.5 mL). The phases were separated and the aqueous phase was extracted with EtOAc (3 × 12.0 mL). The combined organic phases were washed with brine (50.0 mL), dried over $MgSO_4$, filtered and concentrated under reduced pressure. It was then purified by flash column chromatography (CH_2Cl_2 in hexanes, 25 %) to afford **S5** (975 mg, 2.04 mmol, 77.9 %) as a colorless oil.

$\mathbf{R_f}$ (CH_2Cl_2 in hexanes, 15 %): 0.5.

^{1}H-NMR (400 MHz, C_6D_6) δ 7.83 – 7.85 (m, 4H, C16-H), 7.23 – 7.28 (m, 6H, C17-H, C18-H), 5.59 (tq, J = 6.3, 1.3 Hz, 1H, C13-H), 5.45 (tq, J = 7.1, 1.3 Hz, 1H, C4-H), 5.35 (tspt, J = 7.6, 1.5 Hz, 1H, C8-H), 4.35 (d, J = 6.4 Hz, 2H, C14-H), 3.29 (d,

J = 7.6 Hz, 2H, C5-H), 2.05 – 2.11 (m, 2H, C10-H), 1.91 – 1.95 (m, 2H, C9-H), 1.82 (m, 3H, C7-H), 1.56 (s, 3H, C1-H), 1.49 (s, 3H, C3-H), 1.30 (s, 3H, C12-H), 1.20 (s, 9H, C19-(CH_3)$_3$) ppm.

^{13}C-NMR (101 MHz, C_6D_6) δ 136.7 (C2), 136.1 (C11), 135.2 (C16), 134.5 (C18), 130.8 (C6), 129.9 (C15), 128.1 (C17), 125.8 (C4), 125.0 (C13), 120.4 (C8), 61.5 (C5), 39.5 (C9), 30.1 (C14), 27.5 (C10), 27.1 (C19-(CH_3)$_3$), 25.7 (C1), 19.5 (C19), 18.2 (C7), 17.7 (C3), 16.3 (C12) ppm.

HRMS [ESI] *m/z* calculated $C_{30}H_{42}OSSiNa^+$ $[M+Na]^+$: 501.2604, found: 501.2614.

Compound **22** was isolated as a second by-product by flash column chromatography (CH_2Cl_2 in hexanes, 25 to 50 %) as a light yellow oil (74.2 mg, 0.162 mmol, 13.8 %).

R_f (CH_2Cl_2 in hexanes, 15 %): 0.25.

^{1}H-NMR (400 MHz, C_6D_6) δ 7.83 – 7.85 (m, 4H, C22-H), 7.23 – 7.28 (m, 6H, C23-H, C24-H), 5.55 – 5.60 (m, 2H, C13-H, C18-H), 5.31 (tspt, J = 7.7, 1.4 Hz, 1H, C4-H), 4.35 (d, J = 6.3 Hz, 2H, C19-H), 3.04 (d, J = 7.8 Hz, 2H, C5-H), 2.86 – 2.90 (m, 2H, C10-H), 2.06 – 2.12 (m, 2H, C14-H), 1.91 – 1.95 (m, 2H, C15-H), 1.81 – 1.86 (m, 5H, C12-H, C9-H), 1.57 (s, 3H, C3-H), 1.53 (s, 3H, C1-H), 1.30 (s, 3H, C17-H), 1.20 (s, 9H, C12-(CH_3)$_3$), 1.15 (s, 6H, C7-H, C8-H) ppm.

^{13}C-NMR (101 MHz, C_6D_6) δ 136.3 (C16), 135.7 (C22), 134.4 (C24), 134.1 (C2), 129.8 (C21), 129.6 (C11), 128.1 (C23), 125.8 (C13/C18), 124.7 (C13/C18), 120.6 (C4), 61.1 (C19), 44.9 (C6), 41.5 (C9), 39.5 (C15), 28.6 (C7, C8), 27.6 (C10), 27.2 (C20-(CH_3)$_3$), 26.8 (C14), 26.1 (C5), 25.3 (C3), 19.1 (C20), 17.7 (C12), 17.3 (C1), 16.0 (C17) ppm.

HRMS [ESI] *m/z* calculated $C_{35}H_{50}OS_2SiNa^+$ $[M+Na]^+$: 603.3126, found: 603.3127.

(2*E*,6*E*)-3-Methyl-7-((3-methylbut-2-en-1-yl)thio)octa-2,6-dien-1-ol (18)

S5 (975 mg, 2.58 mmol, 1.00 eq.) was dissolved in THF (12.9 mL). No precautions were taken to ensure dry or inert conditions.

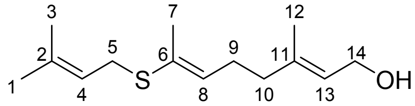

18
$C_{14}H_{24}OS$
240,41 g/mol

TBAF (1.00 M in THF, 3.09 mL, 3.09 mmol, 1.20 eq.) was added and the mixture was stirred for 1 h at rt, before the reaction was terminated by addition of H_2O (10.0 mL). The phases were separated and the aqueous phase was extracted with EtOAc (3 × 15.0 mL). The combined organic phases were washed with brine (50.0 mL), dried over $MgSO_4$, filtered and concentrated under reduced pressure (up to 250 mbar). It was then purified by flash column chromatography (Et_2O in hexanes, 40 %) to afford **18** (433 mg, 1.80 mmol, 69.9 %) as a colorless oil. It was not possible to remove all of the solvent due to the volatile nature of the product.

R_f (Et_2O in hexanes, 50 %): 0.65.

^{1}H-NMR (400 MHz, C_6D_6) δ 5.44 (tsxt, J = 7.14, 1.3 Hz, 1H, C4-H), 5.32 – 5.38 (m, 2H, C8-H, C13-H), 5.97 (d, J = 6.7 Hz, 2H, C14-H), 3.28 (d, J = 7.6 Hz, 2H, C5-H), 2.06 – 2.11 (m, 2H, C10-H), 1.91 – 1.95 (m, 2H, C9-H), 1.83 (m, 3H, C7-H), 1.56 (s, 3H, C1-H), 1.50 (s, 3H, C3-H), 1.44 (s, 3H, C12-H) ppm.

^{13}C-NMR (101 MHz, C_6D_6) δ 137.6 (C11), 135.3 (C2), 130.9 (C6), 125.7 (C4), 125.3 (C8/C13), 120.4 (C8/C13), 59.3 (C5), 39.5 (C9), 30.1 (C14), 27.5 (C10), 25.6 (C1), 18.2 (C7), 17.7 (C3), 16.1 (C12) ppm.

((2*E*,6*E*)-8-Chloro-6-methylocta-2,6-dien-2-yl)(3-methylbut-2-en-1-yl)sulfane (S6)

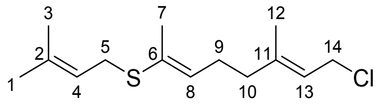

Sx
$C_{14}H_{23}ClS$
258,85 g/mol

2,4,6-Collidine (145 mg, 0.159 mL, 1.20 mmol, 3.00 eq.) and LiCl (250 mg, 5.90 mmol, 14.7 eq.) were dissolved in CH_2Cl_2 (2.70 mL) and cooled to 0 °C. The mixture was cooled to 0 °C, before addition of MsCl (101 mg, 0.068 mL, 0.880 mmol, 2.20 eq.). The reaction mixture was stirred for 10 min. at 0 °C before addition of **18** (96.2 mg, 0.400 mmol, 1.00 eq.) in minimal CH_2Cl_2. The mixture was then stirred for 3 h at 0 °C, before the reaction was terminated by addition of a sat. aq. $NaHCO_3$ solution (3.00 mL). The phases were separated and the aqueous phase was extracted with CH_2Cl_2 (3 × 3.00 mL). The combined organic phases were washed with H_2O (2 × 15.0 mL), brine (15.0 mL), dried over $MgSO_4$, filtered and concentrated under reduced pressure (up to 350 mbar). It was then filtered through silica (Et_2O in hexanes, 10 %) and concentrated under reduced pressure (up to 350 mbar) to afford **S6** as a colorless oil (isolated yield not determined).

$\mathbf{R_f}$ (Et_2O in hexanes, 10 %): 0.95.

^{1}H-NMR (400 MHz, C_6D_6) δ 5.32 – 5.38 (m, 2H, C4-H, C8-H), 5.29 (tsxt, J = 8.0, 1.4 Hz, 1H, C13-H), 3.75 (d, J = 8.0 Hz, 2H, C14-H), 3.29 (d, J = 7.7 Hz, 2H, C5-H), 1.96 – 2.01 (m, 2H, C10-H), 1.79 – 1.84 (m, 5H, C9-H, C7-H), 1.56 (s, 3H, C1-H), 1.50 (s, 3H, C3-H), 1.35 (s, 3H, C12-H) ppm.

^{13}C-NMR (101 MHz, C_6D_6) δ 141.8 (C11), 135.4 (C2), 131.2 (C6), 125.0 (C4), 121.3 (C13), 120.3 (C8), 40.8 (C14), 39.3 (C9), 30.0 (C5), 27.2 (C10), 25.7 (C1), 18.2 (C7), 17.7 (C3), 15.8 (C12) ppm.

(2*E*,6*E*)-3-Methyl-7-((3-methylbut-2-en-1-yl)thio)octa-2,6-dien-1-yl trihydrogen diphosphate, triammonia salt (12)

$(nBu_4N)_3HP_2O_7 \cdot 3\ H_2O$ (631 mg, 0.700 mmol, 1.75 eq.) was dissolved in MeCN (8.00 mL), 3Å MS was added and the mixture was cooled to 0 °C. Allyl chloride **S6** (158 mg, 0.40 mmol, 1.00 eq.) was added and the reaction mixture was stirred for 16 h at rt. The solvent was removed under reduced pressure. The residue was taken up in an ion exchange buffer (1.00 mL, 25.0 mM NH_4HCO_3 in *i*PrOH/H_2O (2.00 % v/v)) and converted into the ammonium salt using an ion exchange column (DOWEX AG 50W-X8 (100 – 200 mesh), NH_4^+-form). After removal of the solvent under reduced pressure, the crude product was dissolved in aq. NH_4HCO_3 solution (0.050 M, 2.00 mL). A solution of MeCN and *i*PrOH (1:1, 10.0 mL) was added and the mixture was centrifuged for 5 min. at 5000 *g*. The supernatant was decanted and the residue redissolved in aq. NH_4HCO_3 solution (0.050 M, 2.00 mL), before addition of MeCN in *i*PrOH (1:1, 10.0 mL). It was then centrifuged again for 5 min. at 5000 *g*, before the supernatant was decanted. The combined supernatants were concentrated under reduced pressure and dried freeze-drying to afford **12** (90.1 mg, 20.0 mmol, 50.0 % o2s) as a yellow amorphous solid.

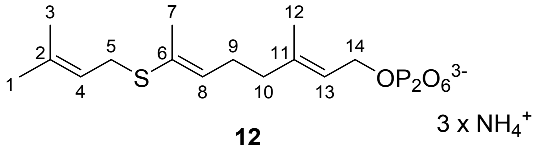

12
$C_{14}H_{35}N_3O_7P_2S$
451,46 g/mol

^{1}H-NMR (400 MHz, D_2O) δ 5.42 – 5.49 (m, 2H, C4-H, C8-H), 5.24 (tsxt, J = 7.7, 1.3 Hz, 1H, C13-H), 4.44 (t, J = 6.6 Hz, 2H, C14-H), 3.35 (d, J = 7.7 Hz, 2H, C5-H), 2.20 – 2.26 (m, 2H, C9-H), 2.08 – 2.12 (m, 2H, C10-H), 1.86 (s, 3H, C7-H), 1.70 (s, 6H, C1-H, C3-H), 1.65 (s, 3H, C12-H) ppm.

^{13}C-NMR (101 MHz, D_2O) δ 142.2 (C6, C11), 137.5 (C2), 128.4 (C4), 120.2 (C8), 118.9 (C13), 62.4 (C14), 38.3 (C9), 29.0 (C5), 26.6 (C10), 24.8 (C1/C3), 17.3 (C7), 17.0 (C12), 15.6 (C1/C3) ppm.

^{31}P-NMR (162 MHz, D_2O) δ –6.38 (d, J = 21.0 Hz), –9.96 (d, J = 22.4 Hz) ppm.

3. Biotransformations, isolation and structure elucidations of new terpenoids

General procedure for the semipreparative biotransformations:

In a 100 mL Erlenmeyer flask was added HEPES buffer (50 mM), DTT (5 mM), NaCl (50 mM), $MgCl_2$ (10 mM). The pH was adjusted with an aqueous solution of NaOH (1 M) until pH = 7.6 or 8.0. Subsequently, the desired ammonium pyrophosphate (1 mM) and 0.1 g/L of the respective STS were added. The amount was calculated for a total volume of 25 mL. The entire procedure was performed a second time and both Erlenmeyer flasks were closed and placed in an incubator (37 °C, 100 rpm, 24 h). Subsequently, a stir bar and GC-MS grade *n*-pentane (20 mL) were added to each Erlenmeyer flask. The mixtures were stirred at rt for 12 h. Afterwards, the mixtures were combined and the phases were separated. Subsequently, the aqueous phase was extracted with *n*-pentane (3 x 50 mL). The combined organic phases were washed with brine, dried over Na_2SO_4, filtered and concentrated to a volume of 1 mL under reduced pressure (750 mbar, 40 °C).

δ-3-Thiocadinene (23)

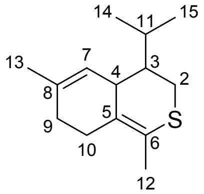

$C_{14}H_{22}S$
222,39 g/mol

Procedure 1: δ-3-Thiocadinene **23** was isolated as a colorless oil. The biotransformation was carried out according to the general procedure described above by using the sesquiterpene synthase Omp7 and FPP derivative **12** as substrate at pH 7.6.

Procedure 2: δ-3-Thiocadinene **23** was isolated as a colorless oil. The biotransformation was carried out according to the general procedure described above by using the sesquiterpene synthase Cop4 and FPP derivative **12** as substrate at pH 7.6. A second major product formed too.

Procedure 3: δ-3-Thiocadinene **23** was isolated as a colorless oil. The biotransformation was carried out according to the general procedure described for the semipreparative biotransformation by using the sesquiterpene synthase Cop4 and FPP derivative **12** as substrate at pH 8.

$\mathbf{R_f}$ (*n*-pentane 100%): 0.35.

RI: 1759.

Manual olefactory analyses: Stale/grumpy, no signs of cannabis or rotten eggs.

^{1}H-NMR: (500 MHz, C_6D_6) δ 5.35 (s, 1H, C7-H), 2.67 (d, *J* = 9.4 Hz, 1H, C4-H), 2.65 – 2.59 (m, 1H, C10-H), 2.48 – 2.39 (m, 2H, C2-H), 2.02 – 1.91 (m, 2H, C9-H, C11-H), 1.84 (m, 5H, C9-H, C12-H), 1.62 (s, 3H, C13-H), 1.60 – 1.46 (m, 1H, C3-H), 0.80 (d, *J* = 6.8 Hz, 3H, C14-H/C15-H), 0.64 (d, *J* = 6.9 Hz, 3H, C14-H/C15-H) ppm.

^{13}C-NMR: (101 MHz, C_6D_6) δ 135.2 (C8), 127.1 (C6), 125.1 (C7), 118.8 (C5), 46.8 (C3), 39.5 (C4), 32.7 (C9), 28.7 (C10), 27.8 (C11), 26.2 (C2), 23.6 (C13), 21.5 (C14/C15), 19.3 (C12), 16.1 (C14/C15) ppm.

The following 2D NMR techniques were utilized: COSY, HSQC, HMBC, NOESY and H2BC. The structure elucidation was further supported by an authentic sample δ-cadinene **25**. The NMR data of δ-cadinene **25** are also attached. It helped for assigning NMR signals.

δ-3-Thiocadinene **23**, ^{1}H NMR, C_6D_6:

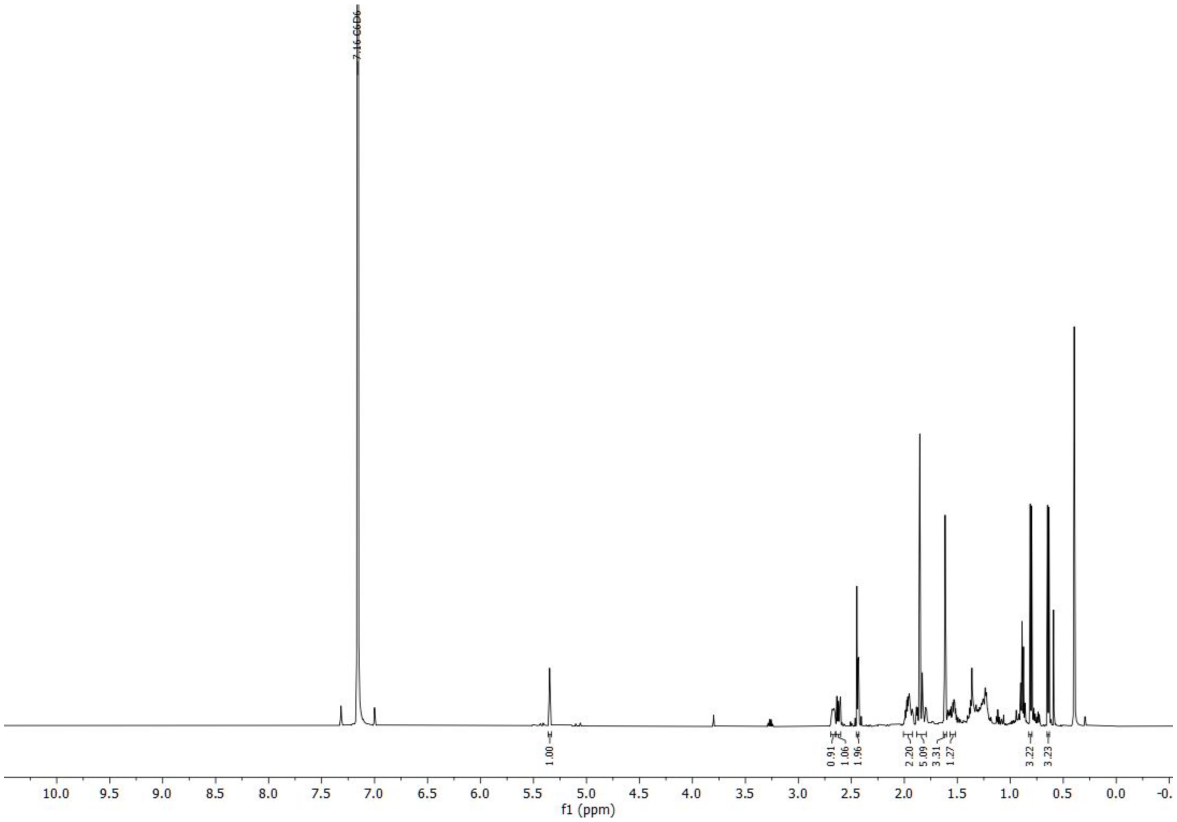

δ-3-Thiocadinene **23**, ^{13}C NMR, C_6D_6:

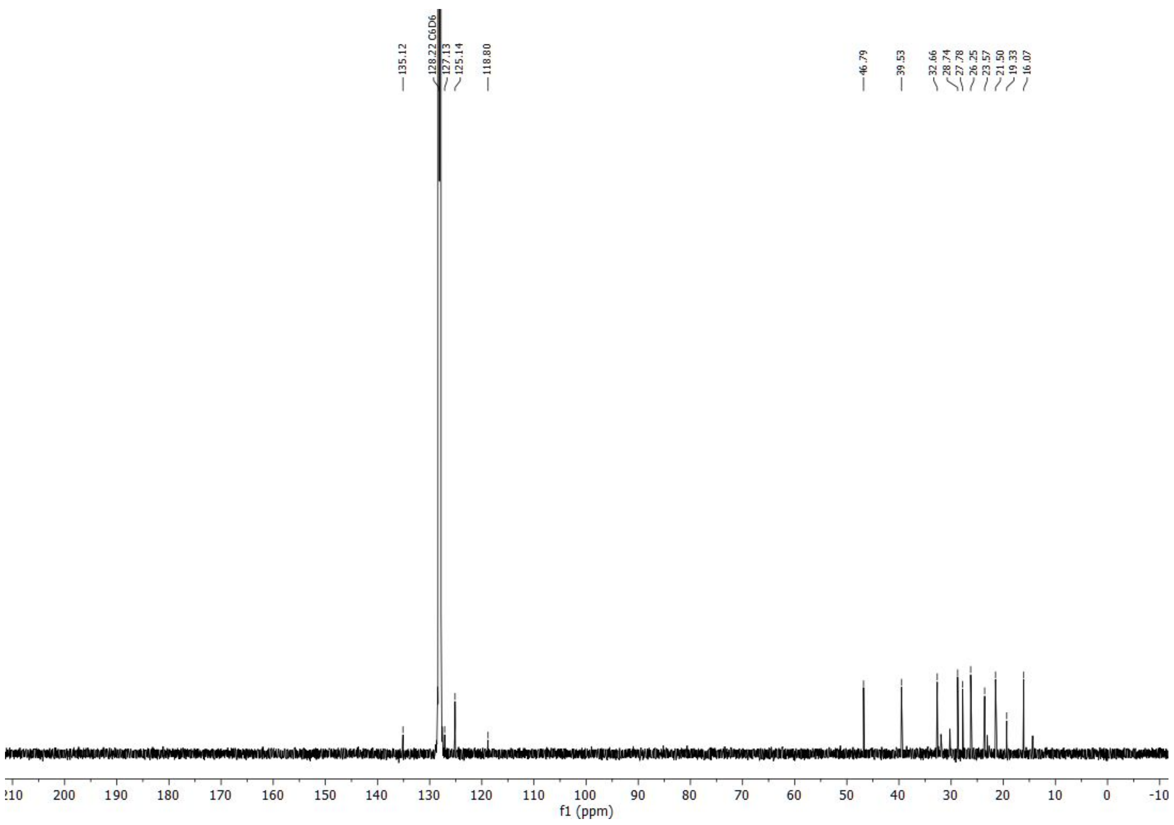

δ-3-Thiocadinene **23**, ^{1}H-^{13}C-HSQC, C_6D_6:

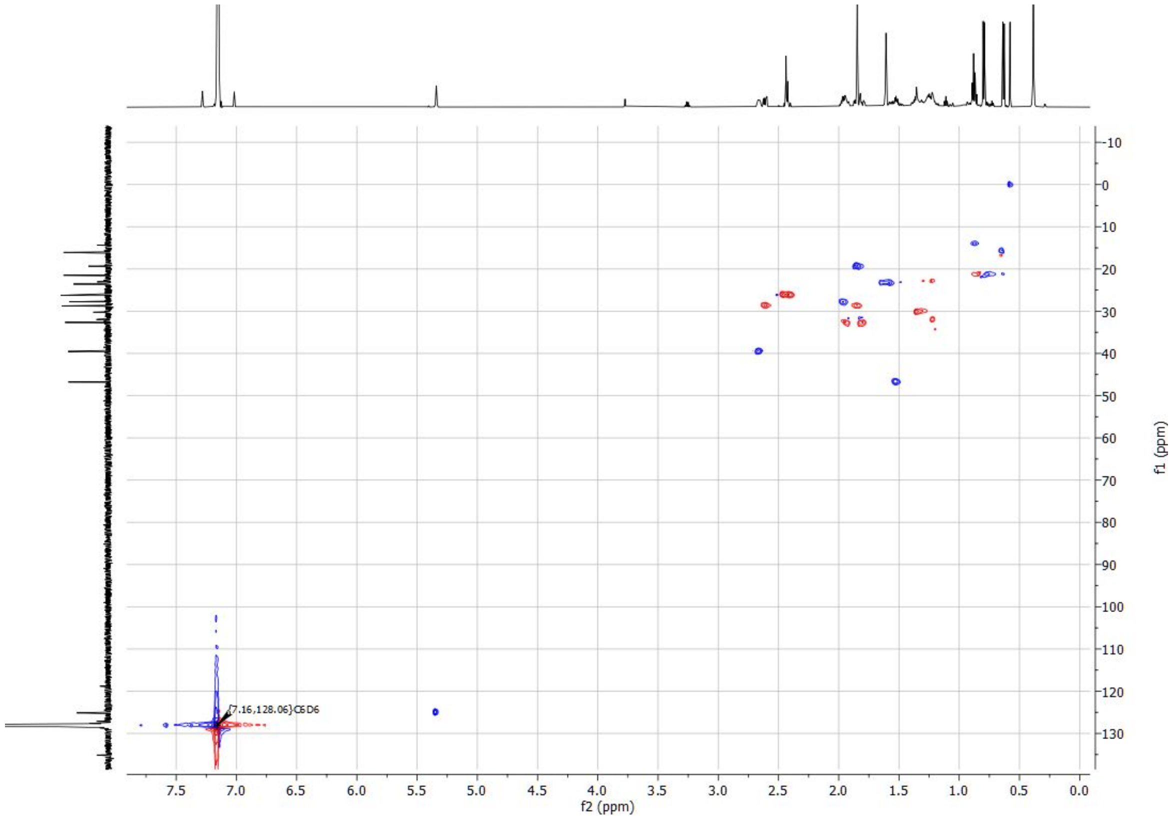

δ-3-Thiocadinene **23**, ^{1}H-^{13}C-HMBC, C_6D_6:

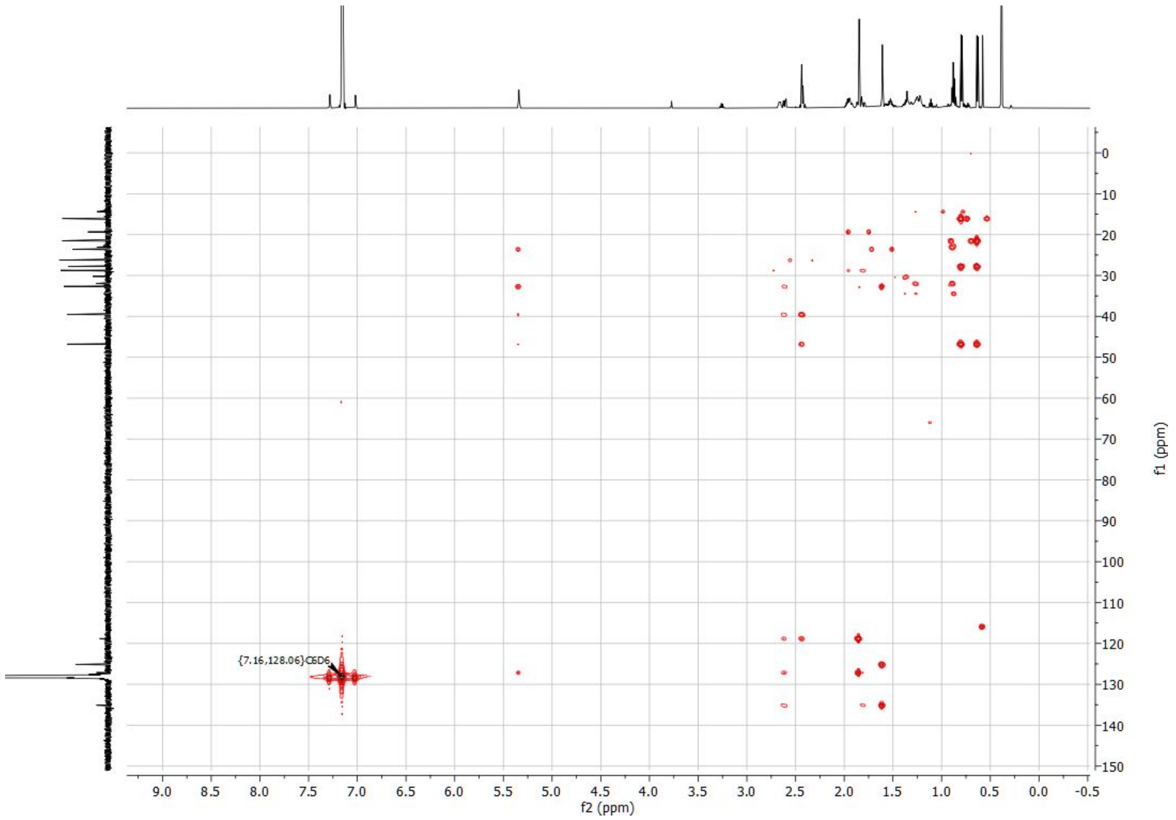

δ-3-Thiocadinene **23**, ^{1}H-^{1}H-COSY, C_6D_6:

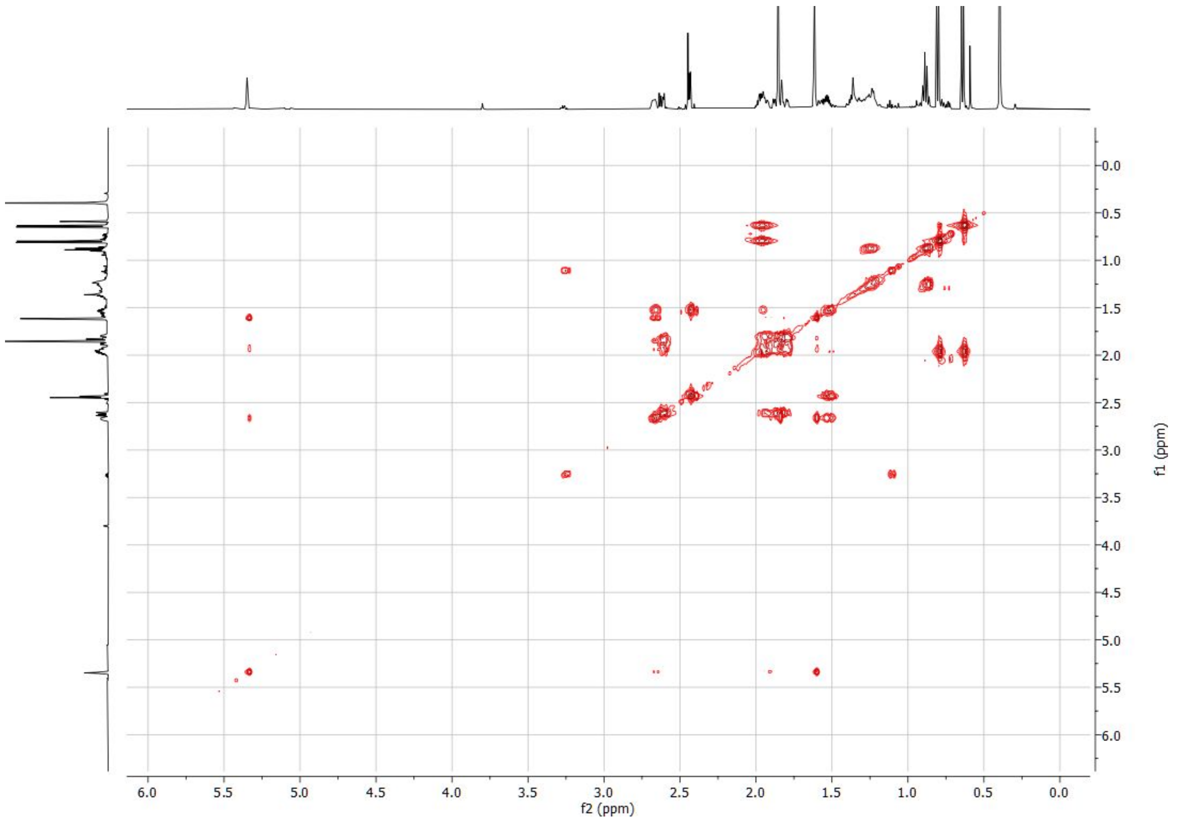

δ-3-Thiocadinene **23**, ^{1}H-^{1}H-NOESY, C_6D_6:

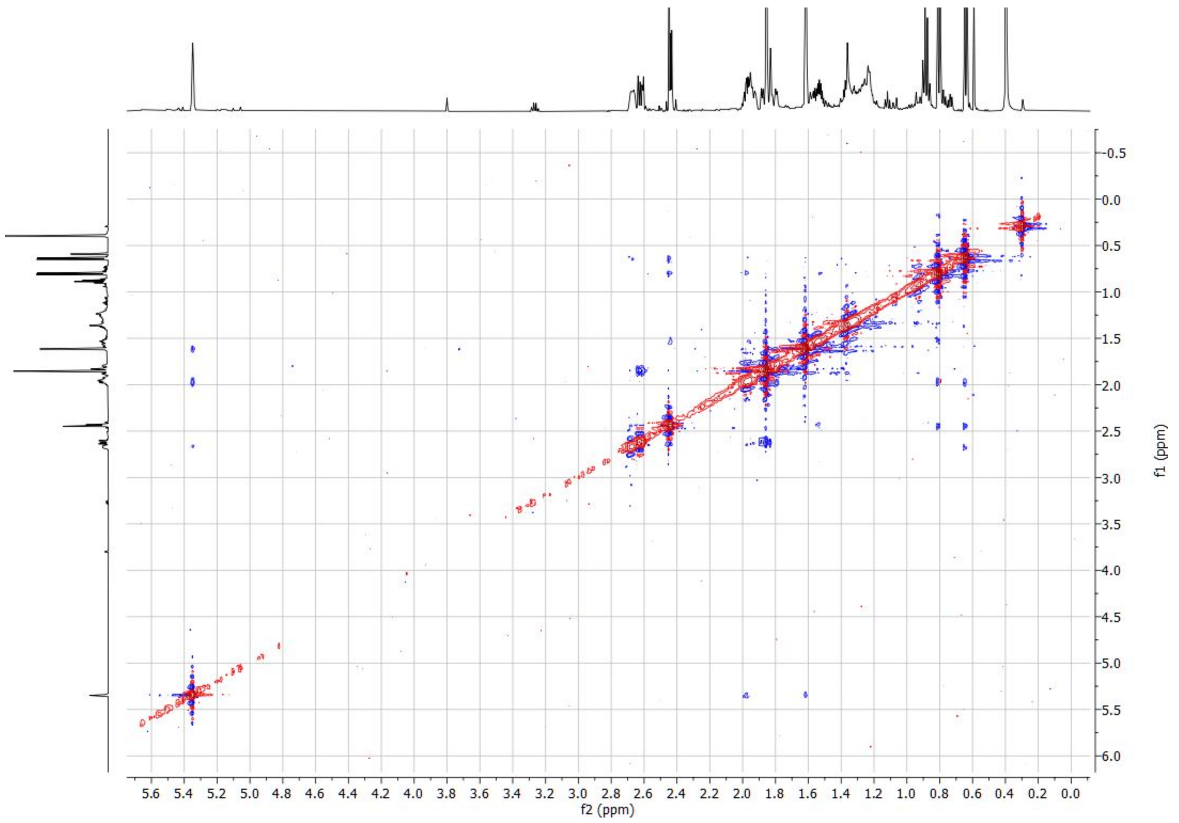

δ-Cadinene (25)

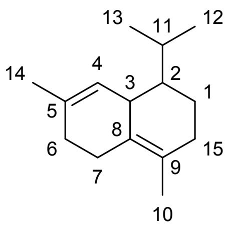

$C_{14}H_{22}S$
222,39 g/mol

δ-Cadinene (**25**) with a purity of over 95.0% (GC) was purchased from TCI and used for NMR measurements without further purification.

^{1}H-NMR: (400 MHz, C_6D_6) δ 5.56 – 5.59 (m, 1H, C4-H), 2.67 – 2.72 (m, 1H, C7-H), 2.60 – 2.67 (m, 1H, C3-H), 2.03 – 2.11 (m, 1H, C11-H), 1.87 – 2.02 (m, 5H,C1-H, C6-H, C7-H), 1.66 – 1.68 (m, 3H, C10-H), 1.63 – 1.65 (m, 3H, C14-H), 1.53 – 1.59 (m, 1H, C1-H), 1.15 – 1.21 (m, 2H, C1-H, C2-H), 0.94 (d, J = 6.9 Hz, 3H, C12-H), 0.80 (d, J = 6.9 Hz, 3H, C13-H) ppm.

^{13}C-NMR: (101 MHz, C_6D_6) δ 134.0 (C5), 130.3 (C8), 125.1 (C4), 124.2 (C9), 45.8 (C2), 40.0 (C3), 32.7 (C15), 32.4 (C6), 27.3 (C7), 27.1 (C11), 23.8 (C14), 22.0 (C12), 21.7 (C1), 18.7 (C10), 15.9 (C12) ppm.

δ-Cadinene **25**, ^{1}H NMR, C_6D_6:

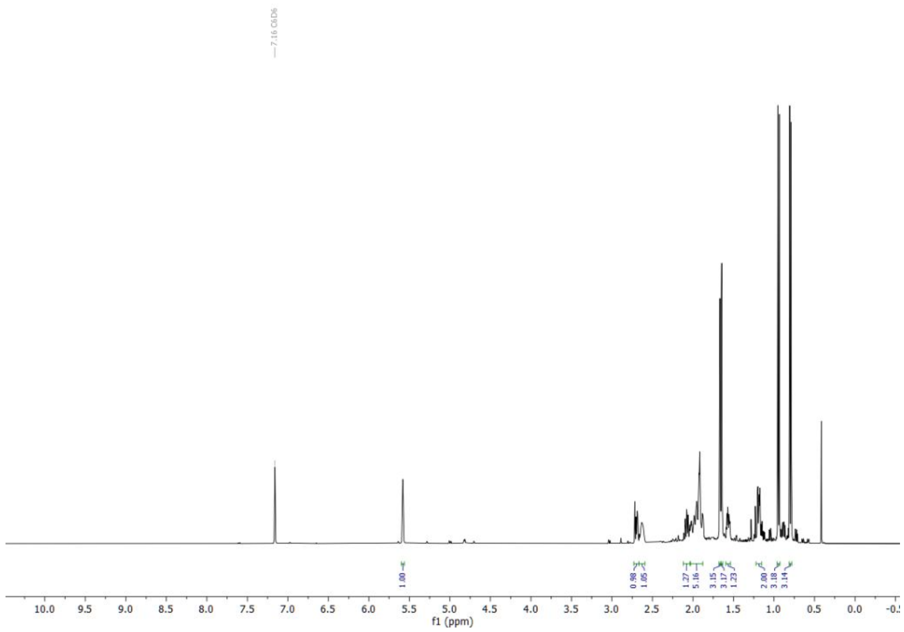

δ-Cadinene **25**, ^{13}C NMR, C_6D_6:

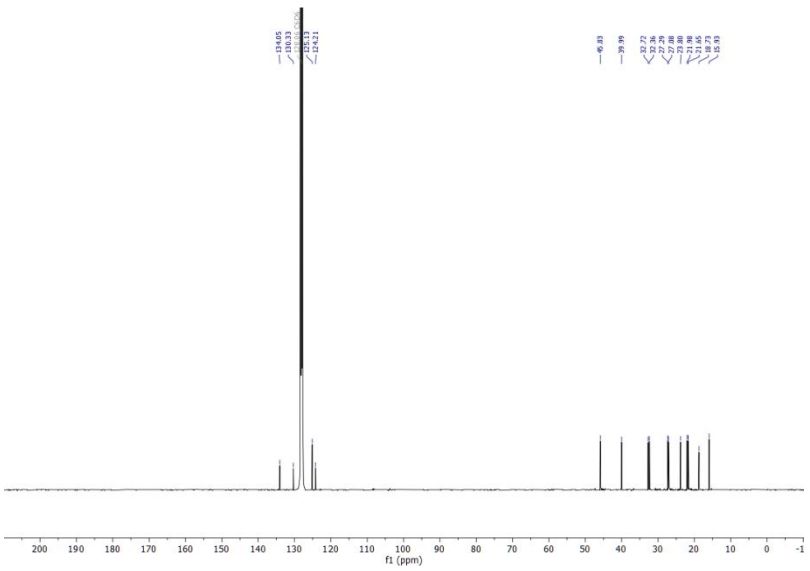

δ-Cadinene **25**, ^{1}H-^{13}C-HSQC, C_6D_6:

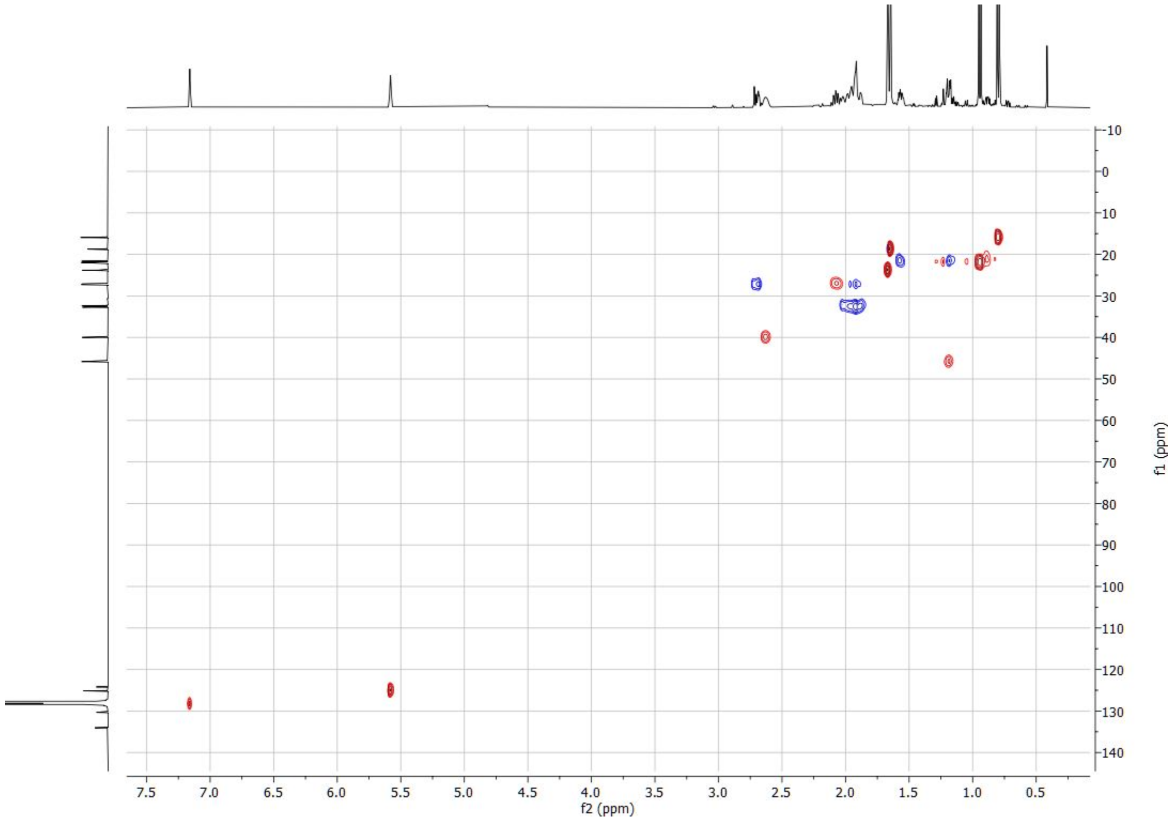

δ-Cadinene **25**, ^{1}H-^{13}C-HMBC, C_6D_6:

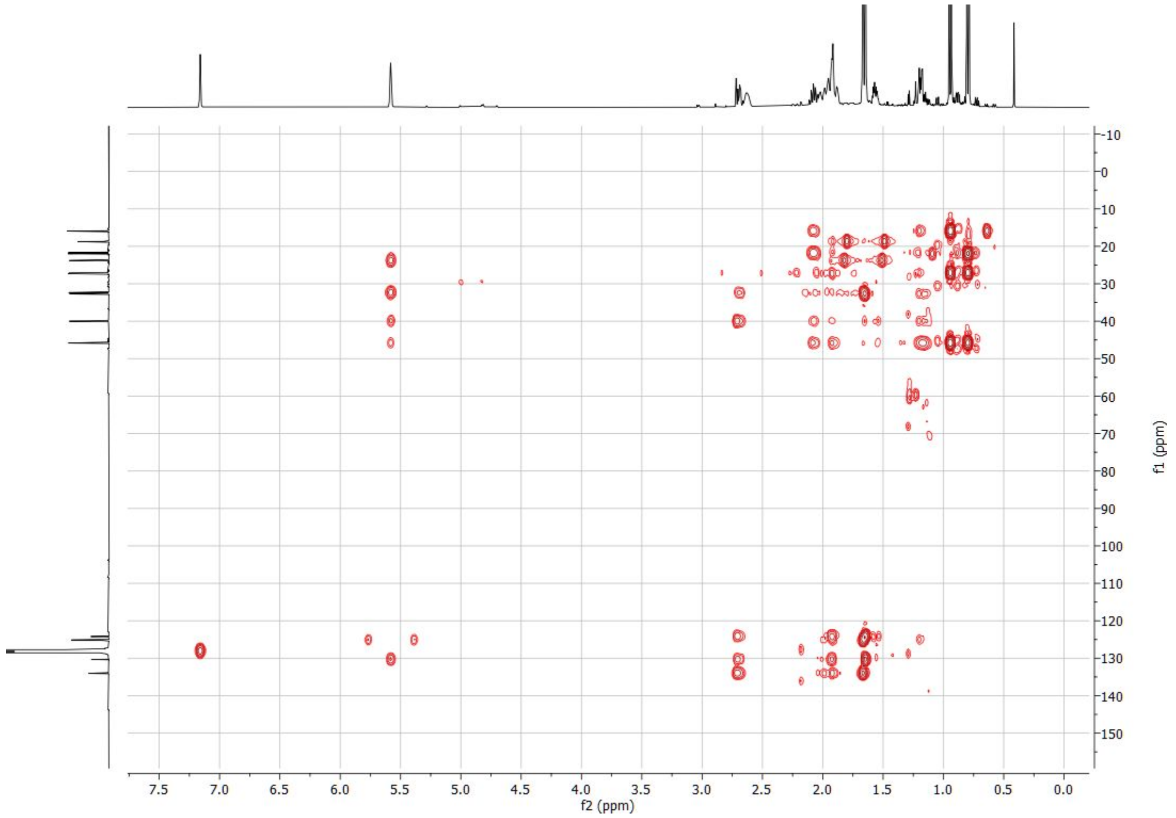

δ-Cadinene **25**, ^{1}H-^{1}H-COSY, C_6D_6:

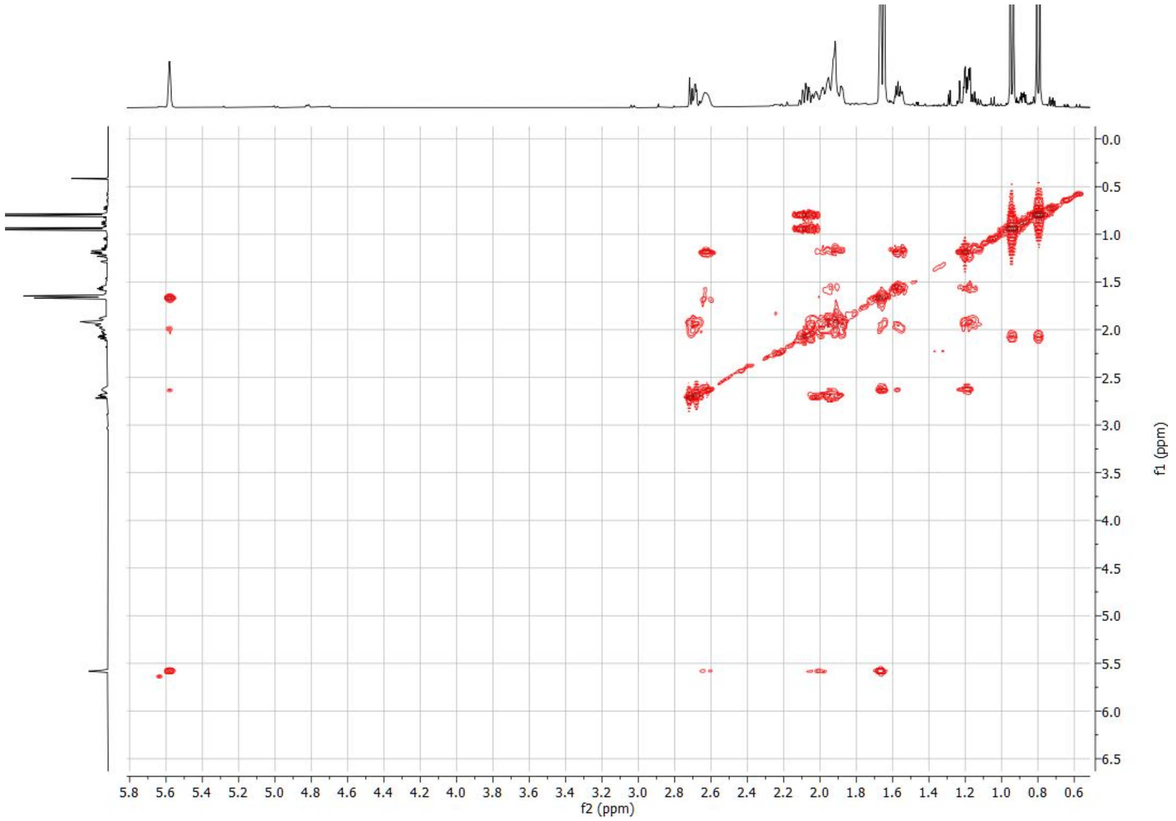

3-Thiocadinol (24)

Procedure 1: 3-Thiocadinol **24** was isolated after biotransformation of **12** with Cop4 (pH 7.6) as one of the two major products, using the general semipreperative biotransformation procedure, as a colorless oil.

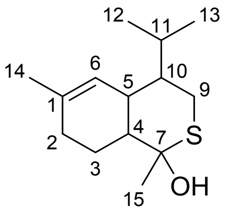

$C_{14}H_{24}OS$
240,41 g/mol

Procedure 2: 3-Thiocadinol **24** was isolated after biotransformation of **12** with Cop4 (pH 8.0) as one of the three major products, using the general semipreperative biotransformation procedure, as a colorless oil.

R_f (10% Et_2O in n-pentane): 0.5.

RI: 1853.

Manual olefactory analyses: Stale/grumpy, no signs of cannabis or rotten eggs.

^{1}H-NMR: (400 MHz, C_6D_6) δ 5.60 (dsxt, J = 6.0, 1.5 Hz, 1H, C6-H), 2.81 (t, J = 12.3 Hz, 1H, C9-H), 2.68 – 2.74 (m, 1H, C5-H), 2.20 (dd, J = 13.1, 2.9 Hz, 1H, C9-H), 1.96 – 2.11 (m, 2H, C3-H, C11-H), 1.83 – 1.90 (m, 3H, C2-H, C4-H), 1.72 (tt, J = 11.6, 3.1 Hz, 1H, C10), 1.59 (p, J = 1.1 Hz, 3H, C14), 1.45 – 1.51 (m, 1H, C3-H), 1.32 – 1.34 (m, 4H, C15-H), 0.78 (dd, 7.0, 4.3 Hz, 6H, C12-H, C13-H) ppm.

^{13}C-NMR: (101 MHz, C_6D_6) δ 134.1 (C1), 125.5 (C6), 80.2 (C7), 46.3 (C4), 44.7 (C10), 34.6 (C5), 31.2 (C2), 30.3 (C15), 27.4 (C11), 24.5 (C9), 23.7 (C14), 21.6 (C3), 21.5 (C12/C13), 15.5 (C12/C13) ppm.

3-Thiocadinol **24**, ^{1}H NMR, C_6D_6:

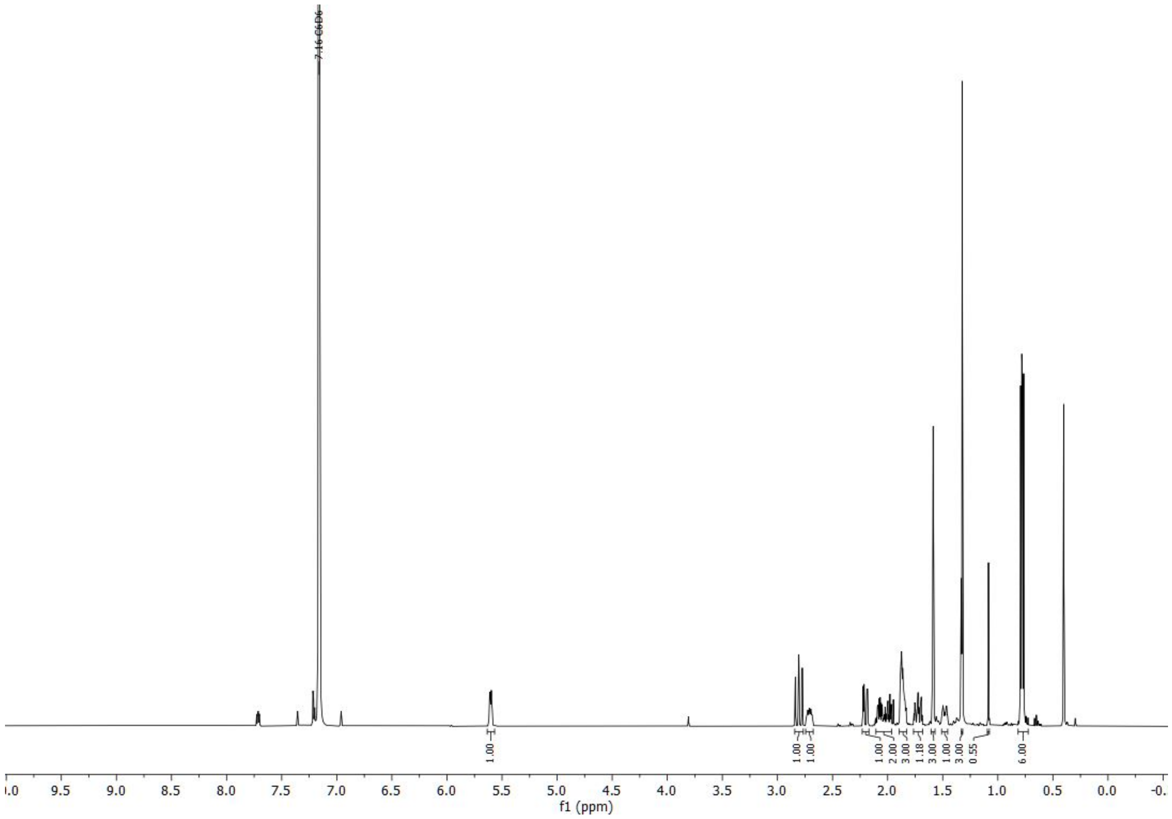

3-Thiocadinol **24**, ^{13}C NMR, C_6D_6:

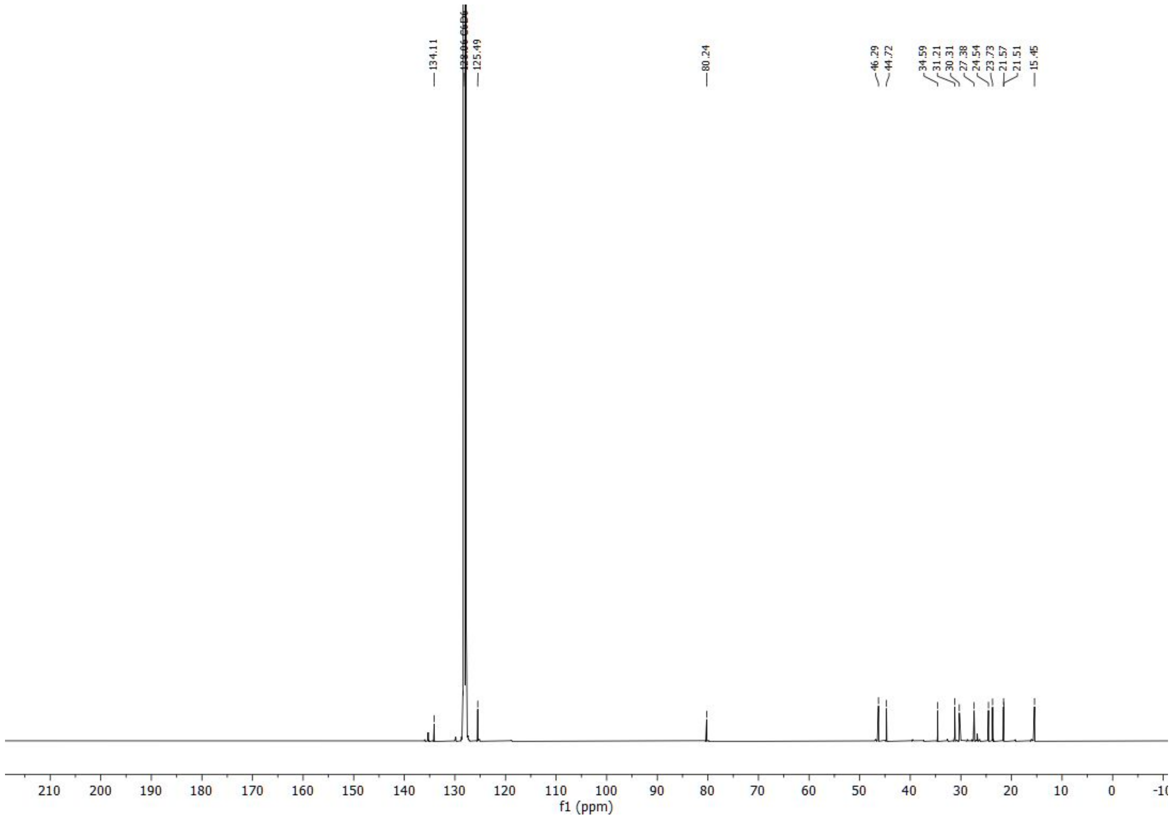

3-Thiocadinol **24**, ^{1}H-^{13}C-HSQC, C_6D_6:

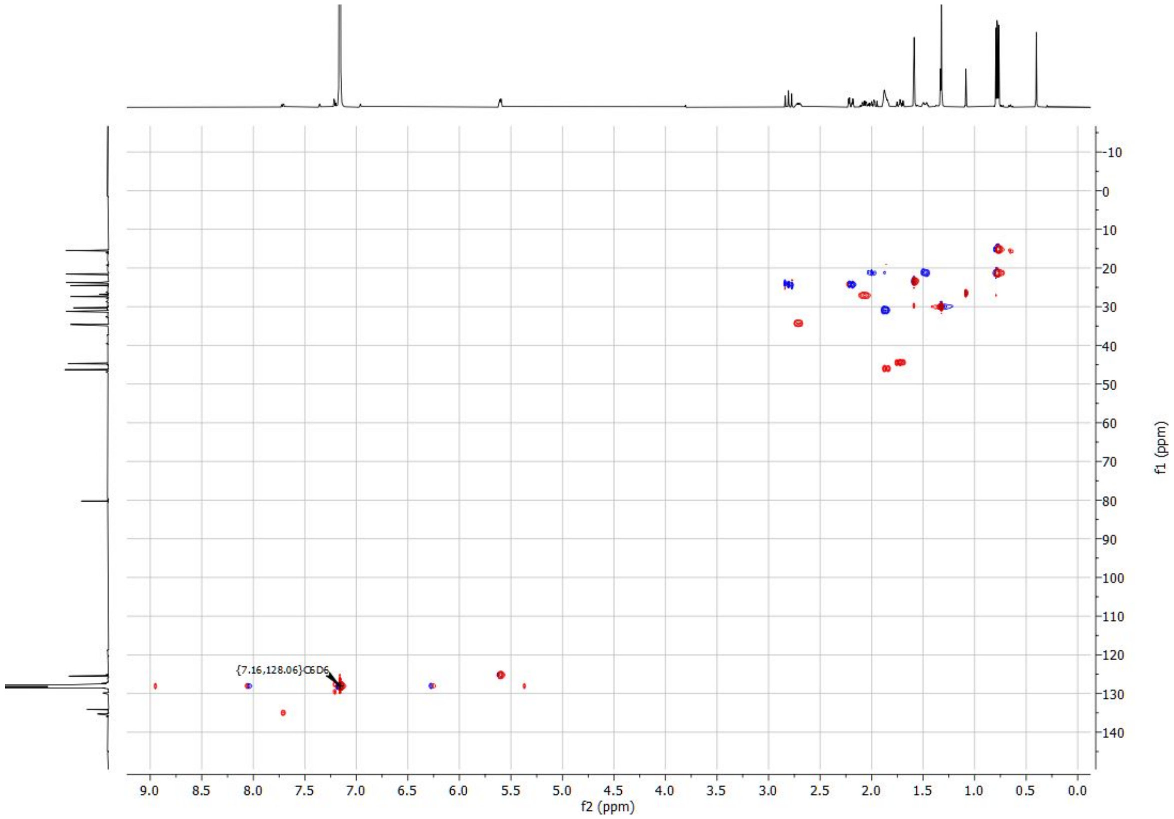

3-Thiocadinol **24**, ^{1}H-^{13}C-HMBC, C_6D_6:

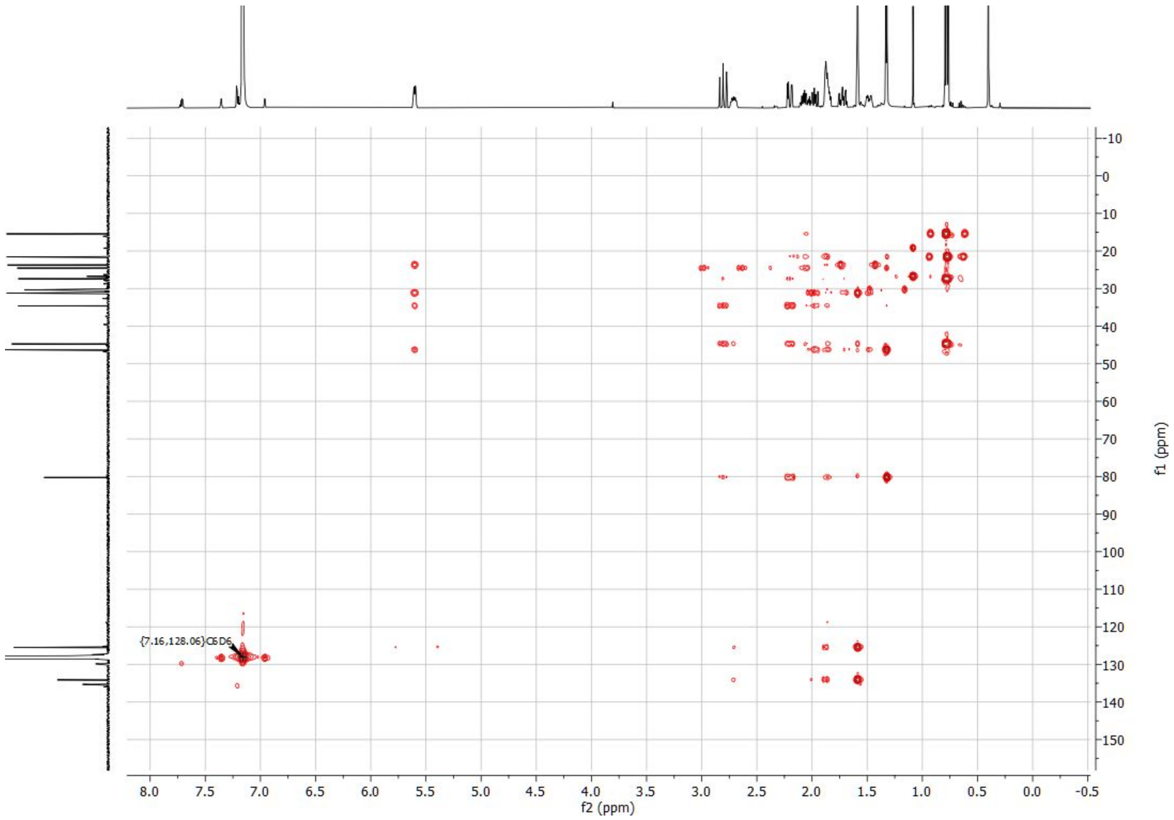

3-Thiocadinol **24**, ^1H-^1H-COSY, C_6D_6:

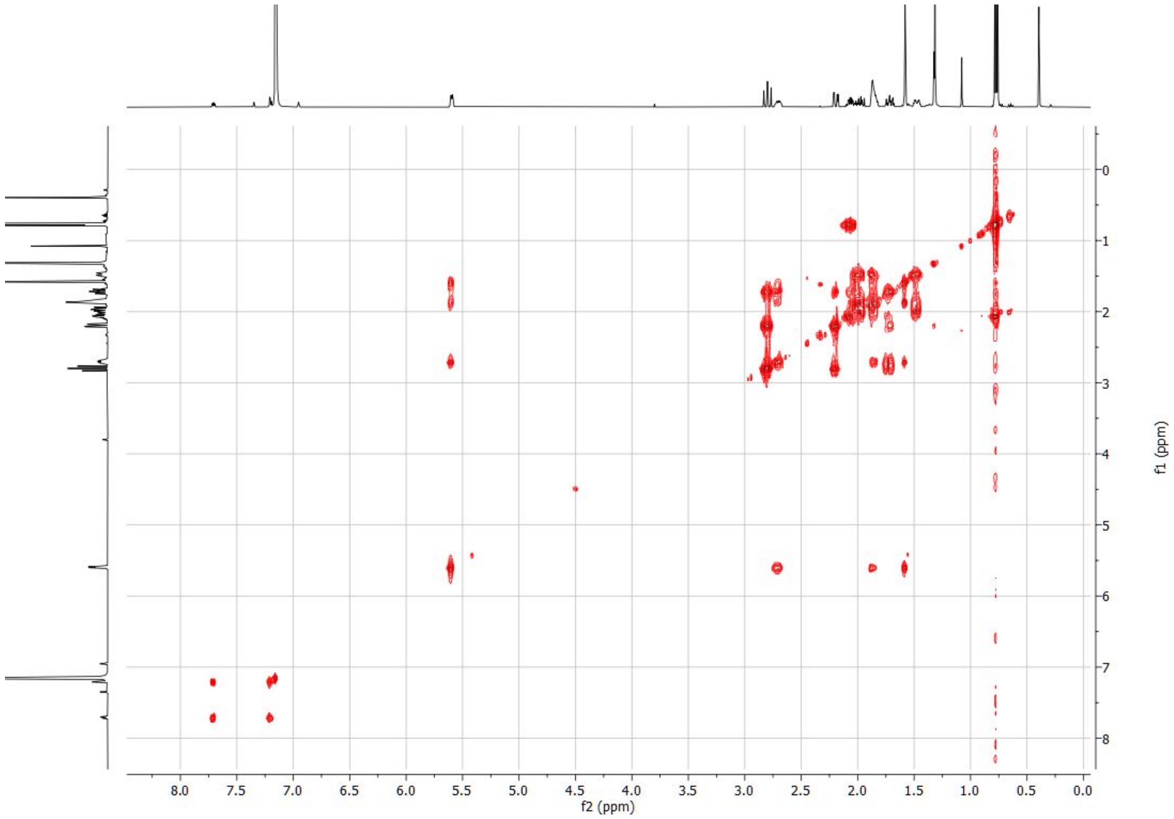

3-Thiocadinol **24**, ^1H-^1H-NOESY, C_6D_6:

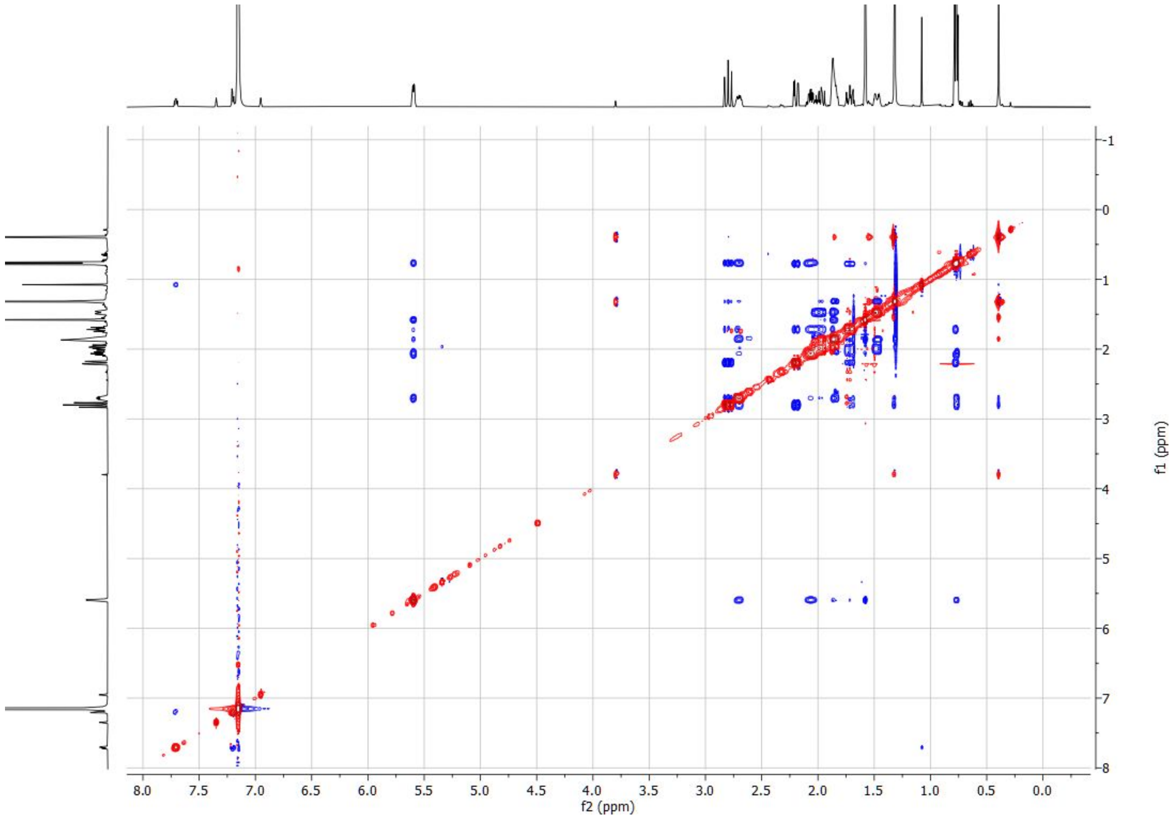

Ketothiol 27

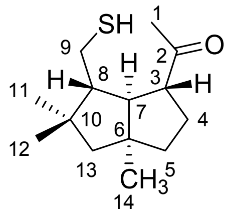

$C_{14}H_{24}OS$

240,41

The ketothiol **27** was isolated as a colorless oil. The biotransformation was carried out according to the general procedure described for the semipreparative biotransformation by using the sesquiterpene synthase BcBot2 and FPP derivative **12** as substrate at pH 7.6.

R$_f$ (10% Et_2O in *n*-pentane): 0.38.

RI: 1759.

Manual olefactory analyses: Unpleasant, spicy

^{1}H-NMR: (600 MHz, C_6D_6) δ 2.78 (td, J = 6.8, 3.0 Hz, 1H, C3-H), 2.39 (ddd, J = 11.7, 6.9, 3.0 Hz, 1H, C9-H), 2.30 (dd, J = 10.2, 3.0 Hz, 1H, C7-H), 2.08 – 2.16 (m, 1H, C9-H), 1.89 (s, 3H, C1-H), 1.64 (q, J = 6.9 Hz, 2H, C4-H), 1.24 – 1.42 (m, 4H, C5-H, C13-H), 1.16 (s, 3H, C14-H), 1.10 – 1.16 (m, 1H, C8-H), 0.87 (s, 3H, C12-H), 0.69 (s, 3H, C11-H) ppm.

^{13}C-NMR: (151 MHz, C_6D_6) δ 208.7 (C2), 60.4 (C3), 60.2 (C-8), 58.5 (C7), 57.6 (C13), 48.6 (C6), 44.2 (C10), 43.2 (C5), 30.4 (C4), 29.7 (C14), 29.6 (C12), 28.8 (C1), 25.4 (C9), 23.4 C11) ppm.

Ketothiol **27**, ^{1}H NMR, C_6D_6:

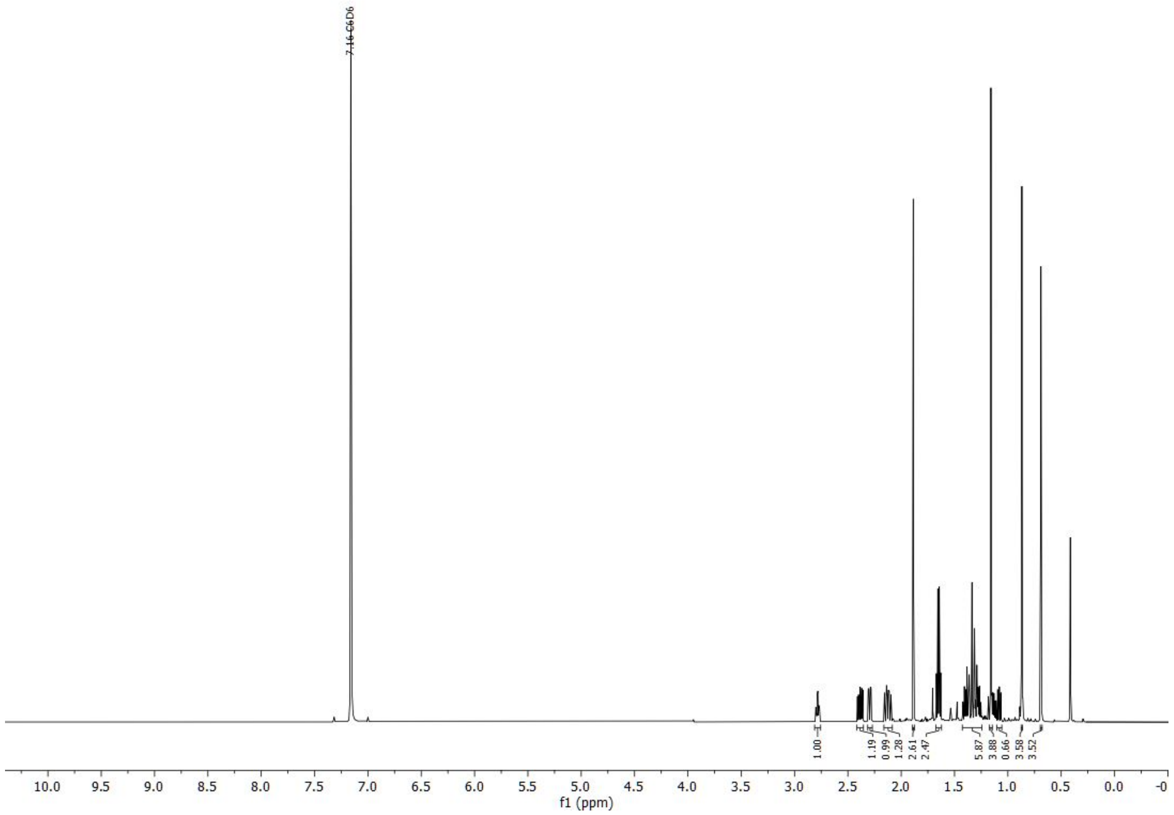

Ketothiol **27**, ^{13}C NMR, C_6D_6:

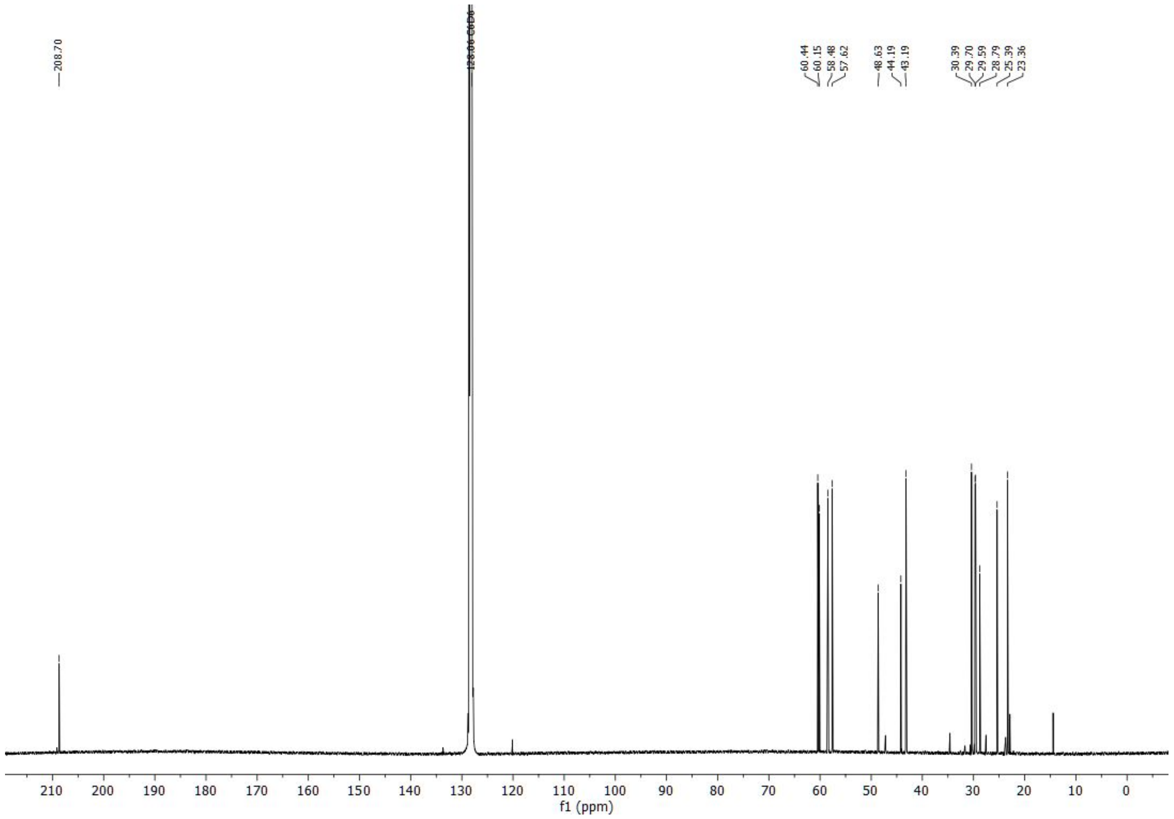

Ketothiol **27**, ^{1}H-^{13}C-HSQC, C_6D_6:

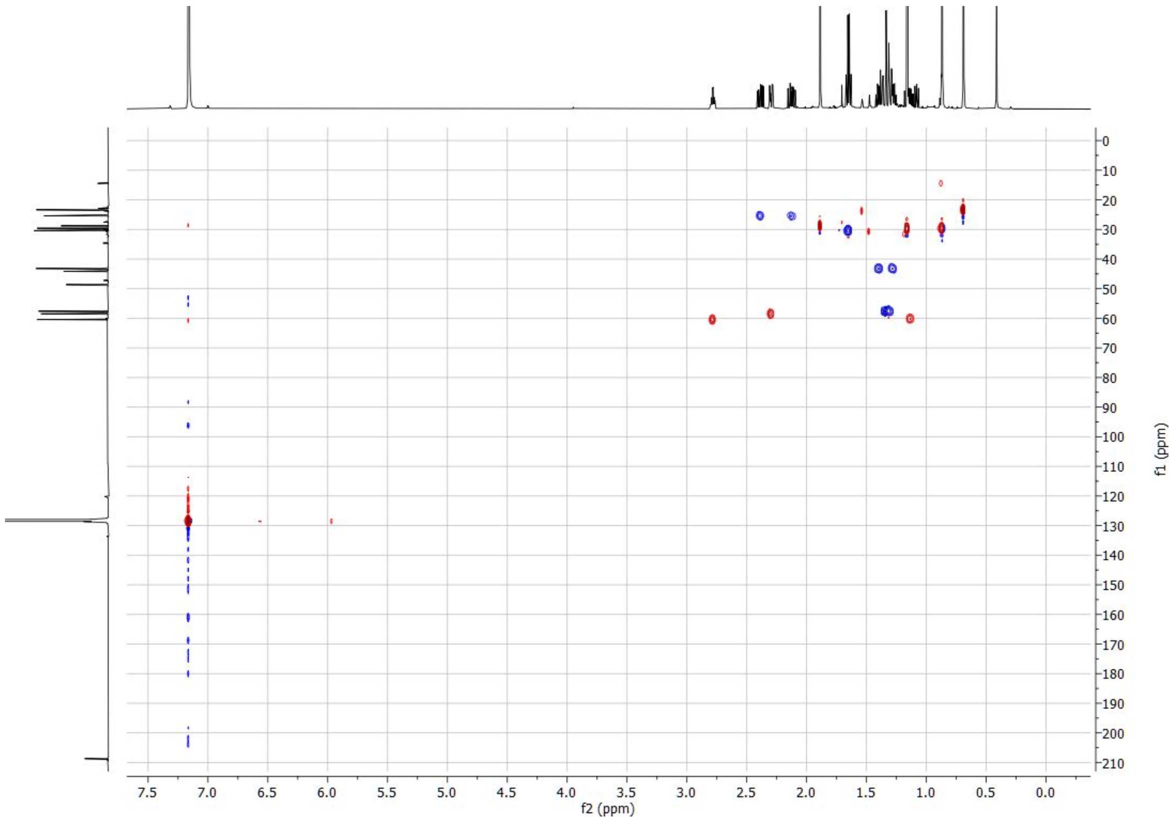

Ketothiol **27**, ^{1}H-^{13}C-HMBC, C_6D_6:

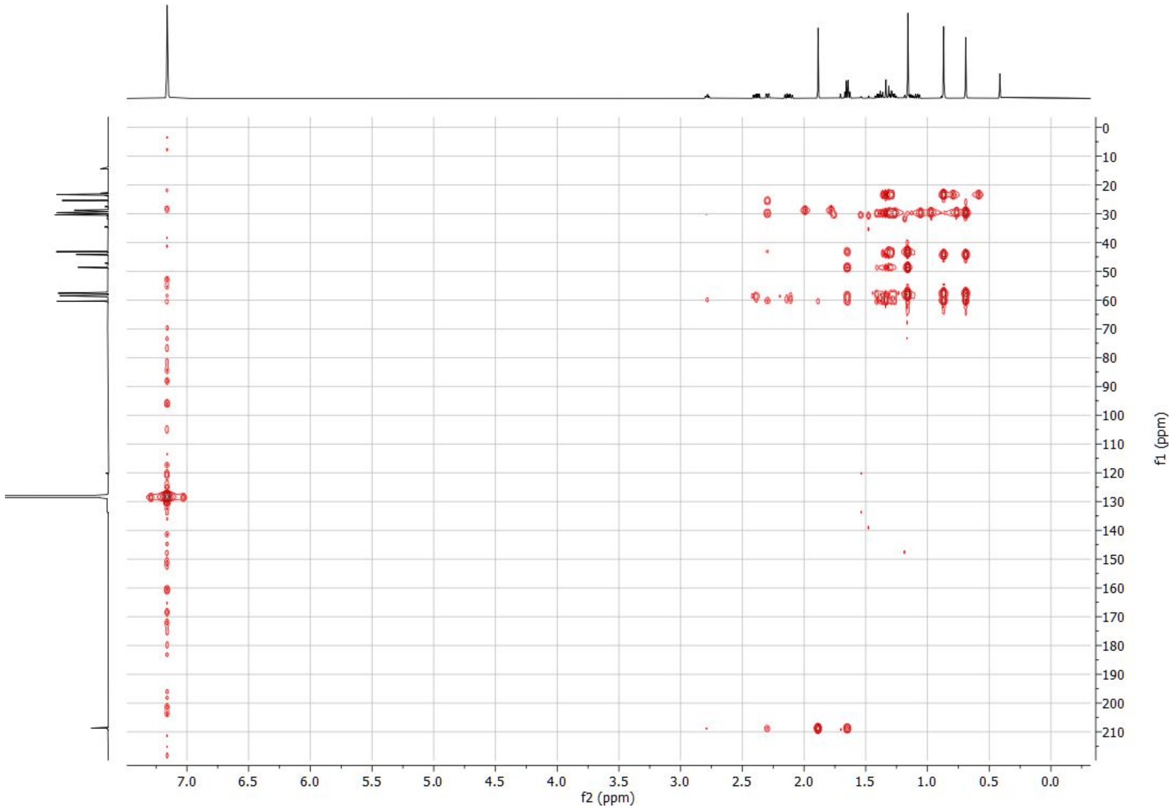

Ketothiol **27**, ^{1}H-^{1}H-COSY, C_6D_6:

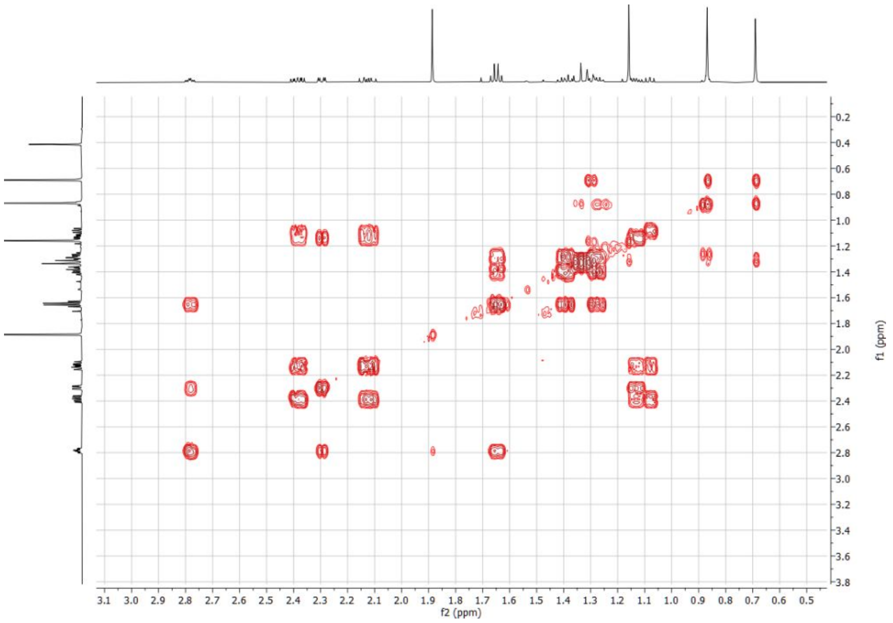

Ketothiol **27**, ^{1}H-^{1}H-NOESY, C_6D_6:

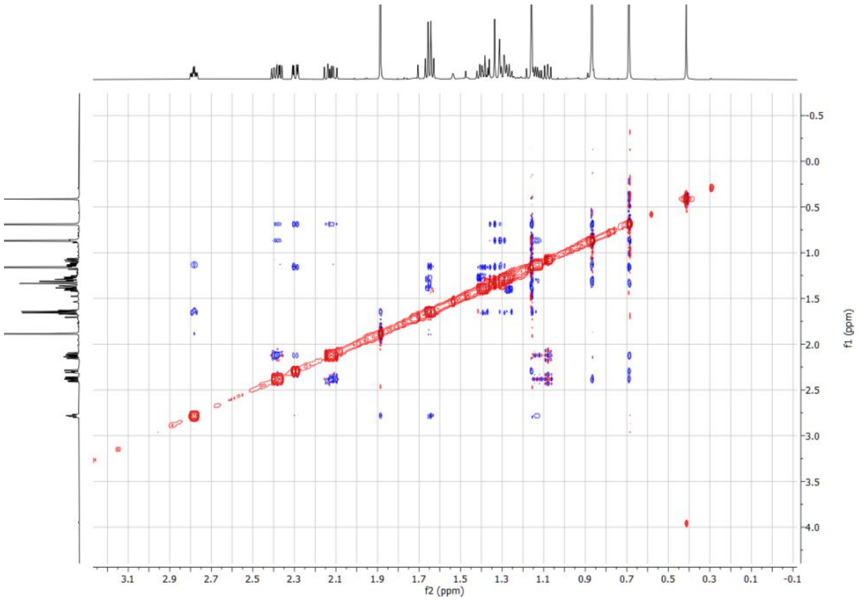

4. References SI

(S1) Oberhauser, C.; Harms, V.; Seidel, K.; Schröder, B.; Ekramzadeh, K.; Beutel, S.; Winkler, S.; Lauterbach, L.; Dickschat, J. S.; Kirschning, A., Exploiting the Synthetic Potential of Sesquiterpene Cyclases for Generating Unnatural Terpenoids, *Angew. Chem. Int. Ed.* **2018**, *36*, 11802–11806.

(S2) Nelsen, D. L.; Gagné, M. R., Platinum(II) Enyne Cycloisomerization Catalysis: Intermediates and Resting States, *Organometallics* **2009**, *28*, 950–952.

(S3) Mohr, P. J.; Halcomb, R. L., Total synthesis of (+)-phomactin a using a B-alkyl Suzuki macrocyclization, *J. Am. Chem. Soc.* **2003**, *125*, 1712–1713.

(S4) Isaka, R.; Yu, L.; Sasaki, M.; Igarashi, Y.; Fuwa, H., Complete Stereochemical Assignment of Campechic Acids A and B, *J. Org. Chem.* **2016**, *81*, 3638–3647.

5. Copies of NMR spectra

3-Methylbut-2-ene-1-thiol **20**, ^{1}H-NMR, 400 MHz, $CDCl_3$:

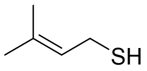

20

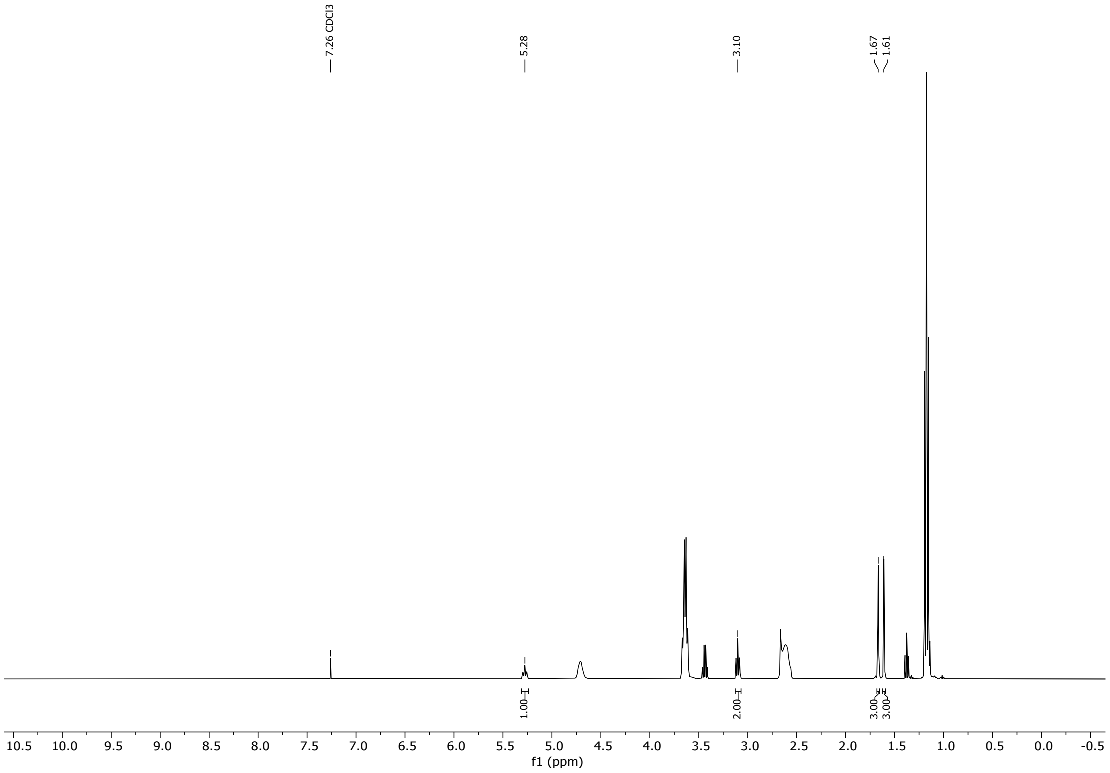

3-Methylbut-2-ene-1-thiol **20**, ^{13}C-NMR, 101 MHz, $CDCl_3$:

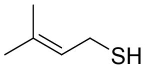

20

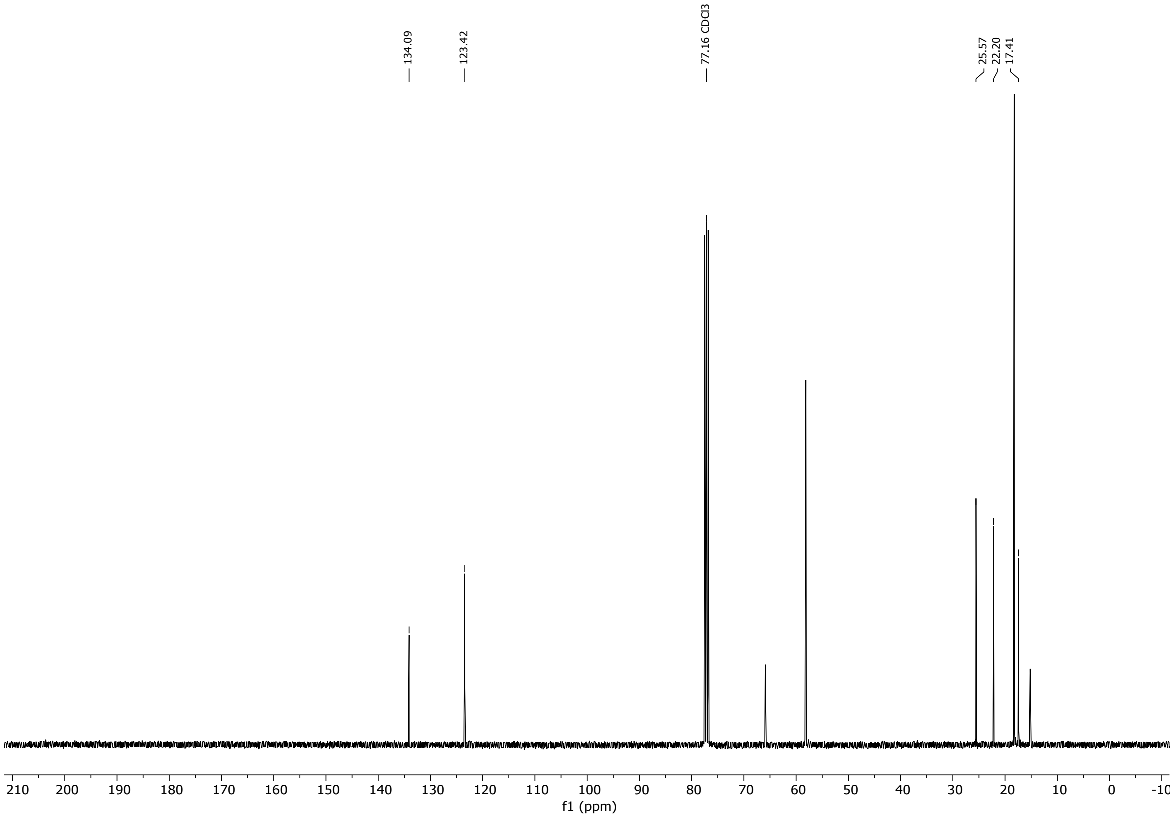

(*E*)-5-(3,3-Dimethyloxiran-2-yl)-3-methylpent-2-en-1-yl acetate **S1**, ^{1}H-NMR, 400 MHz, $CDCl_3$:

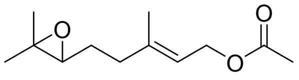

S1

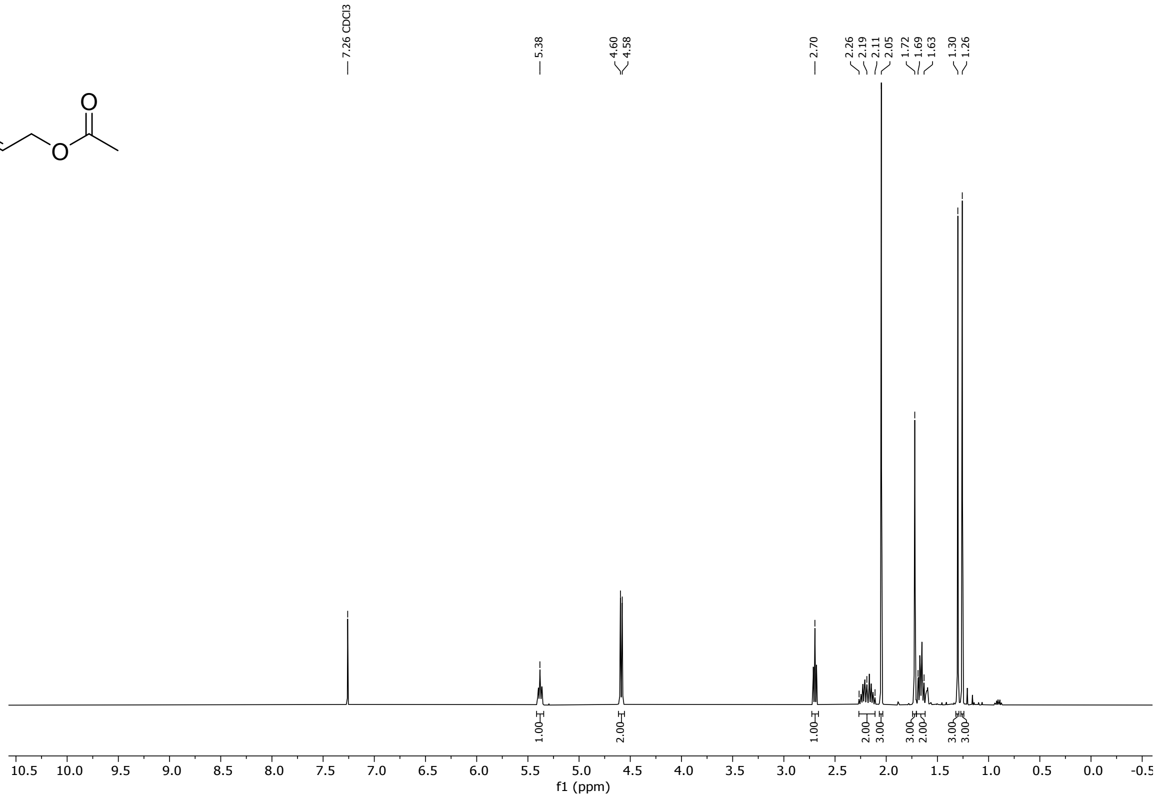

(*E*)-5-(3,3-Dimethyloxiran-2-yl)-3-methylpent-2-en-1-yl acetate **S1**, ^{13}C-NMR, 101 MHz, $CDCl_3$:

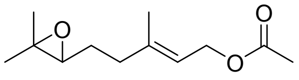

S1

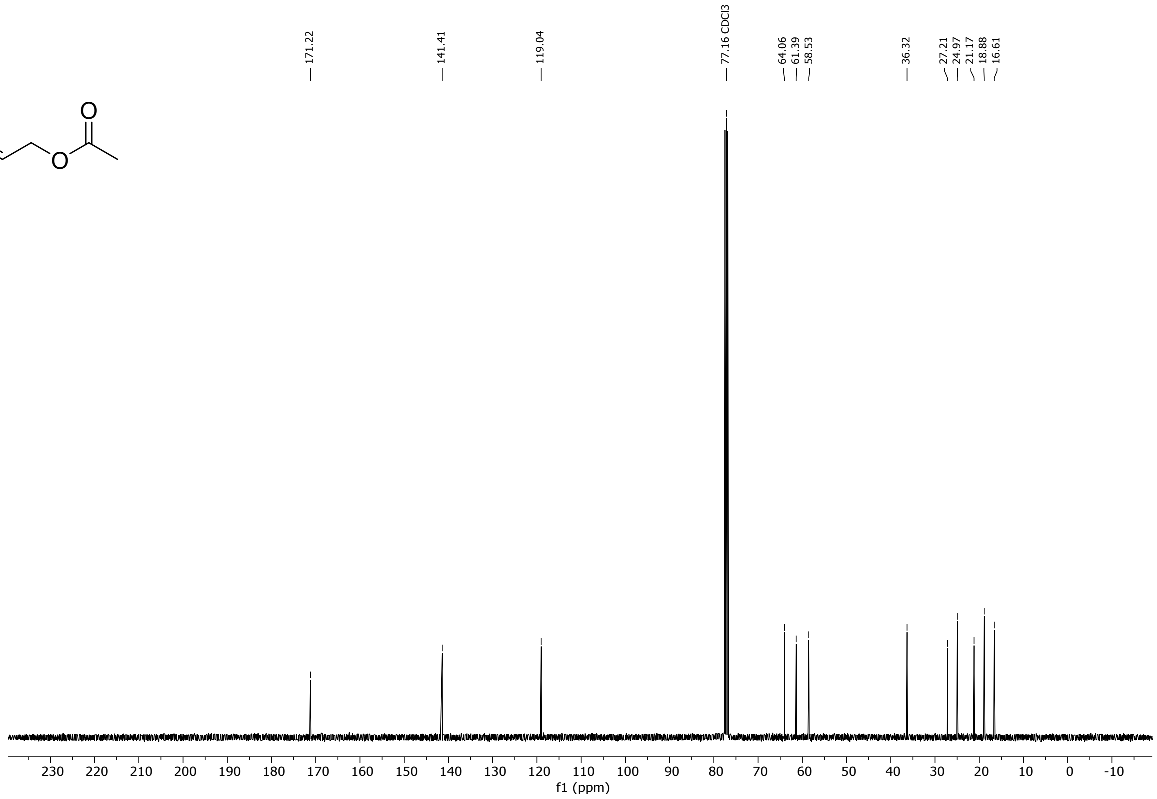

(*E*)-3-Methyl-6-oxohex-2-en-1-yl acetate **S2**, ^{1}H-NMR, 400 MHz, $CDCl_3$:

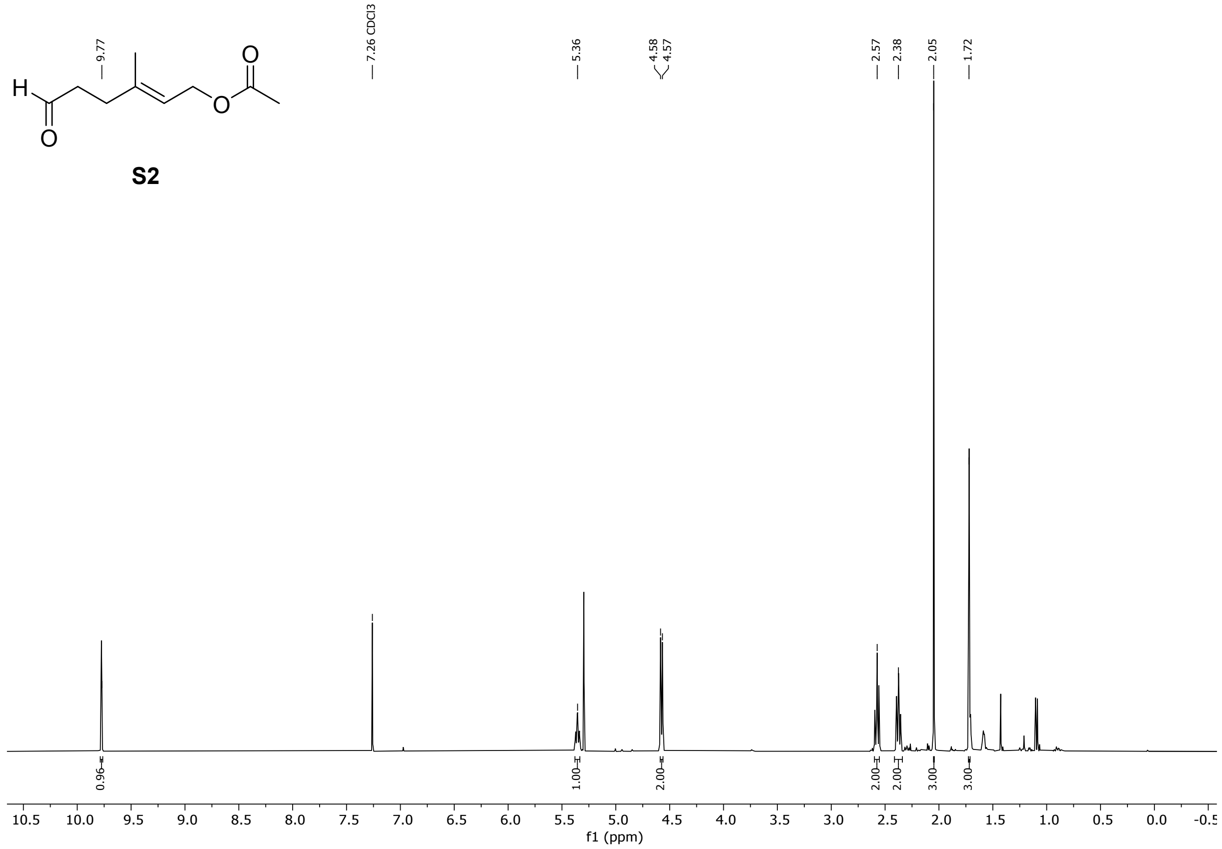

(*E*)-3-Methyl-6-oxohe x-2-en-1-yl acetate **S2**, ^{13}C-NMR, 101 MHz, $CDCl_3$:

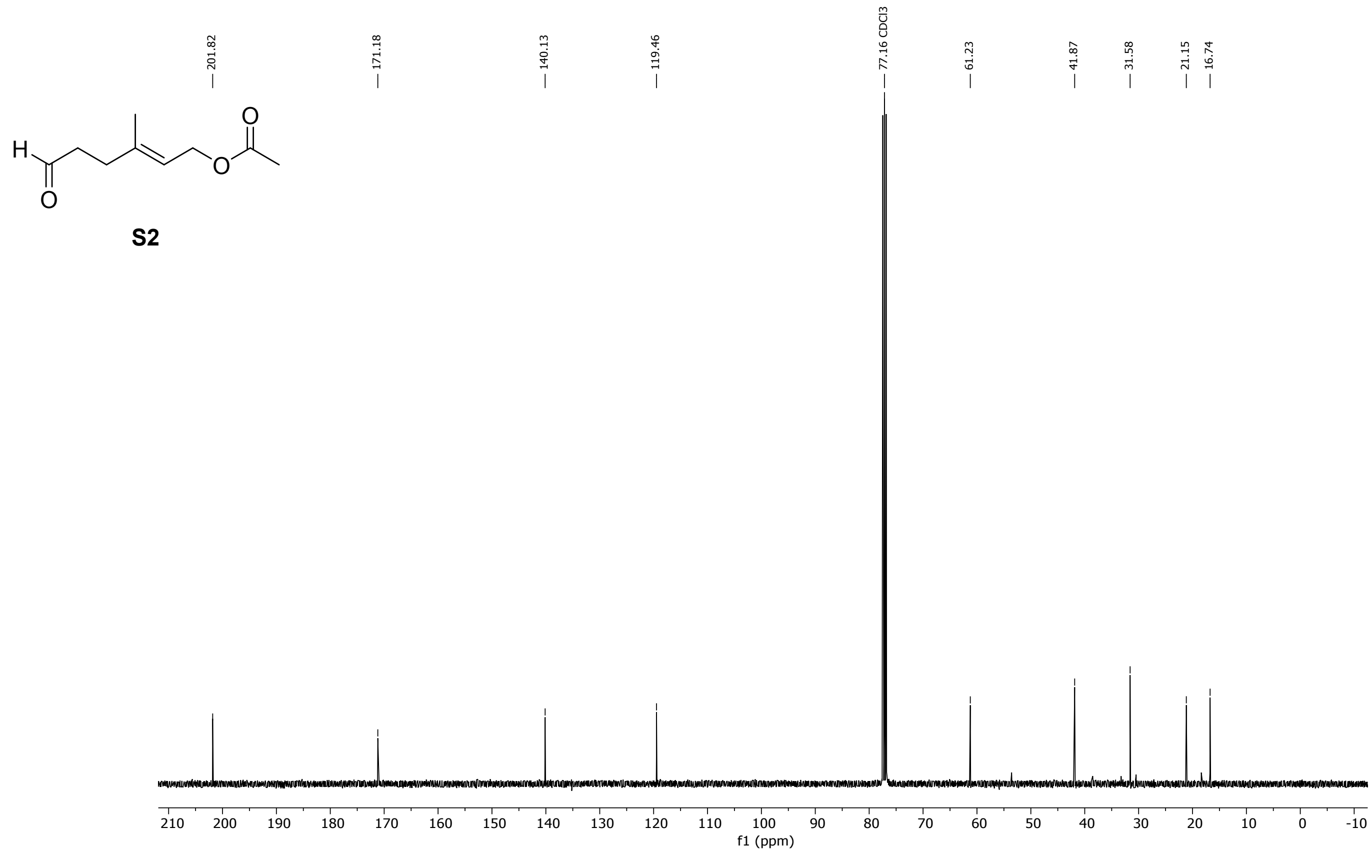

(*E*)-7,7-Dibromo-3-methylhepta-2,6-dien-1-yl acetate **S3**, ^{1}H-NMR, 400 MHz, $CDCl_3$:

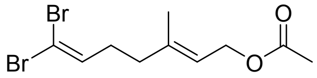

S3

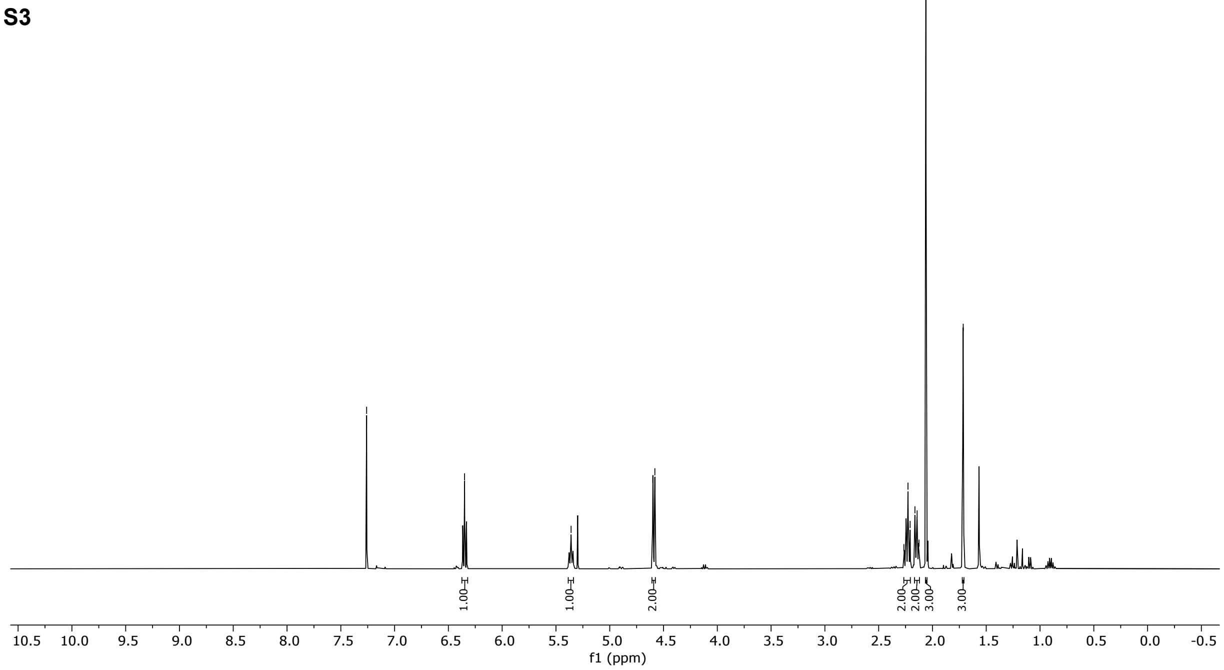

(*E*)-7,7-Dibromo-3-methylhepta-2,6-dien-1-yl acetate **S3**, ^{13}C-NMR, 101 MHz, $CDCl_3$:

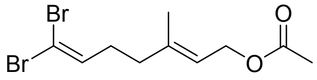

S3

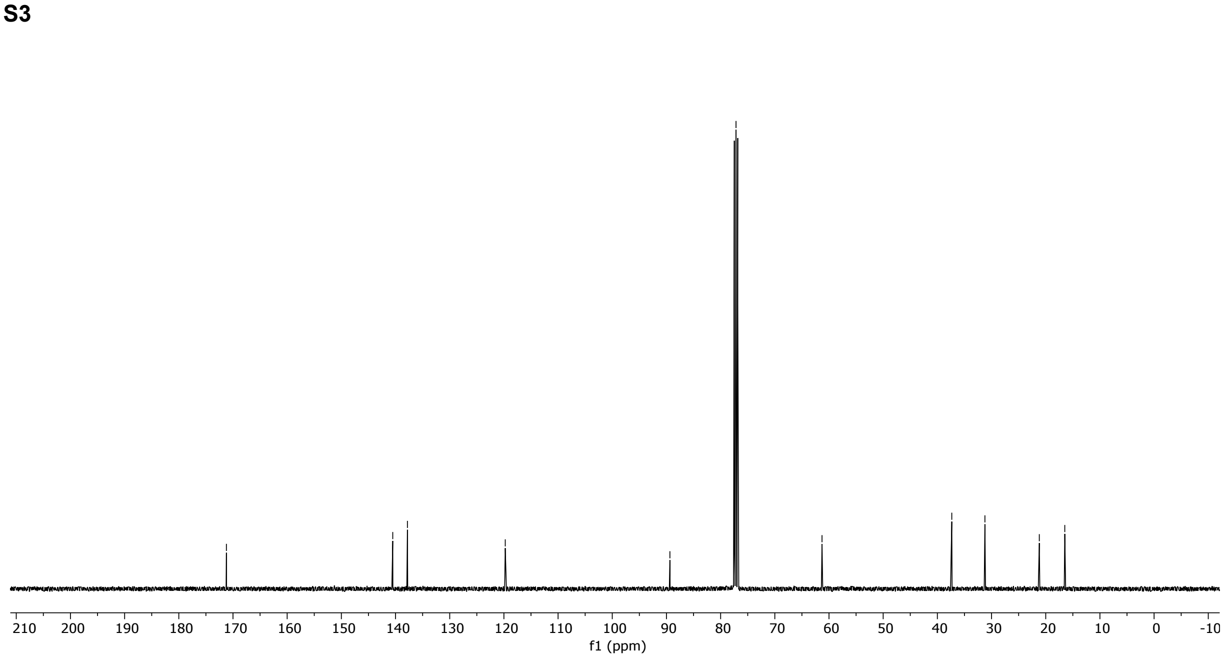

(*E*)-7,7-Dibromo-3-methylhepta-2,6-dien-1-ol **14**, ^{1}H-NMR, 400 MHz, $CDCl_3$:

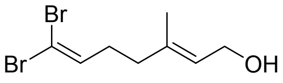

14

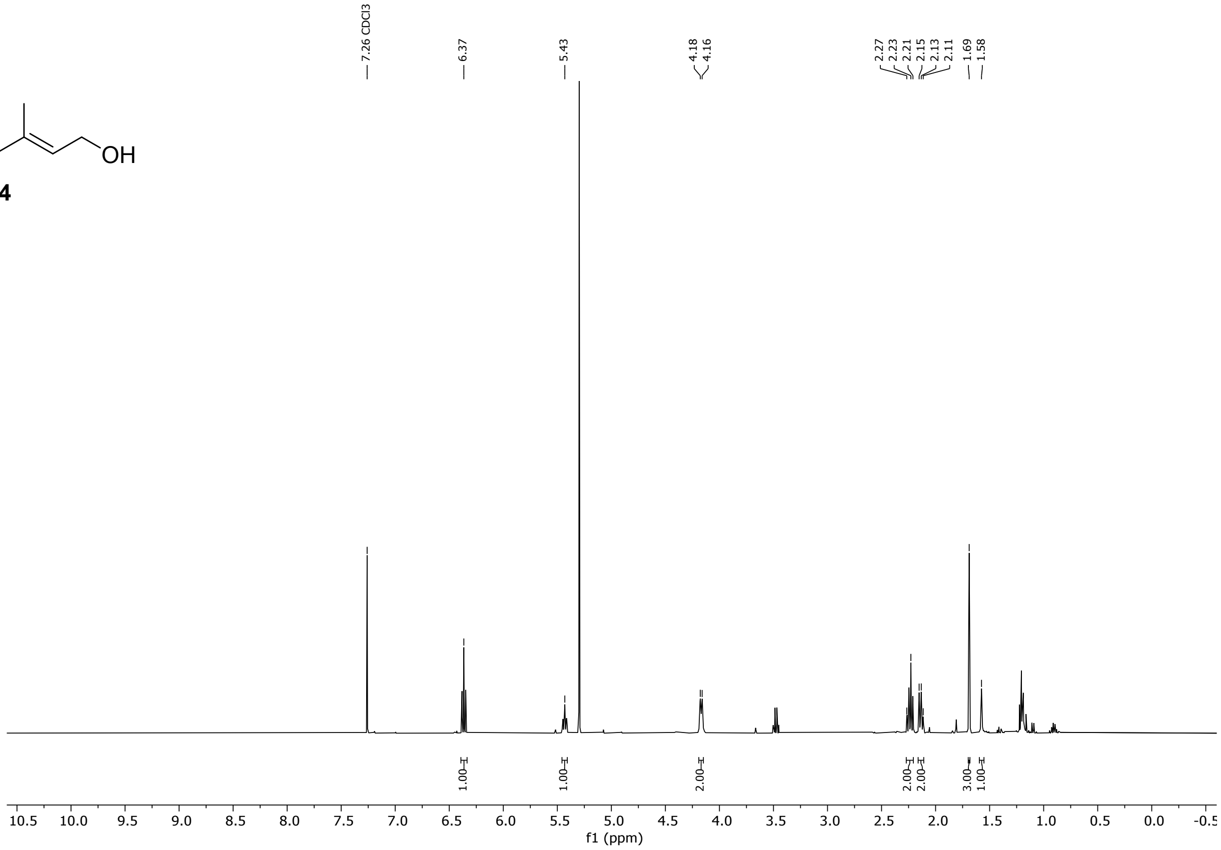

(*E*)-7,7-Dibromo-3-methylhepta-2,6-dien-1-ol **14**, ^{13}C-NMR, 101 MHz, $CDCl_3$:

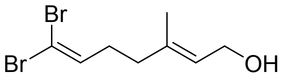

14

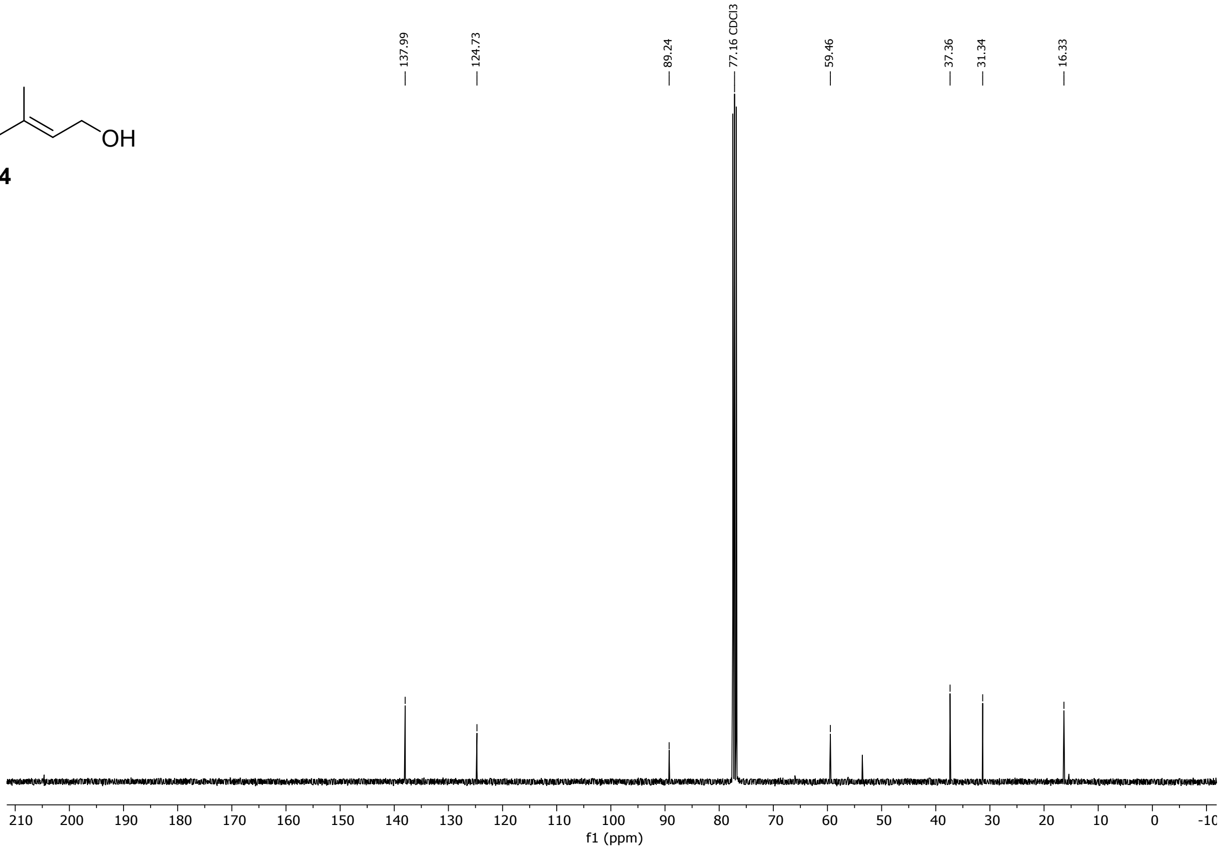

(E)-*tert*-Butyl((7,7-dibromo-3-methylhepta-2,6-dien-1-yl)oxy)diphenylsilane **15**, ^{1}H-NMR, 400 MHz, $CDCl_3$:

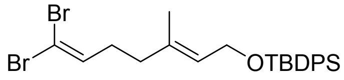

15

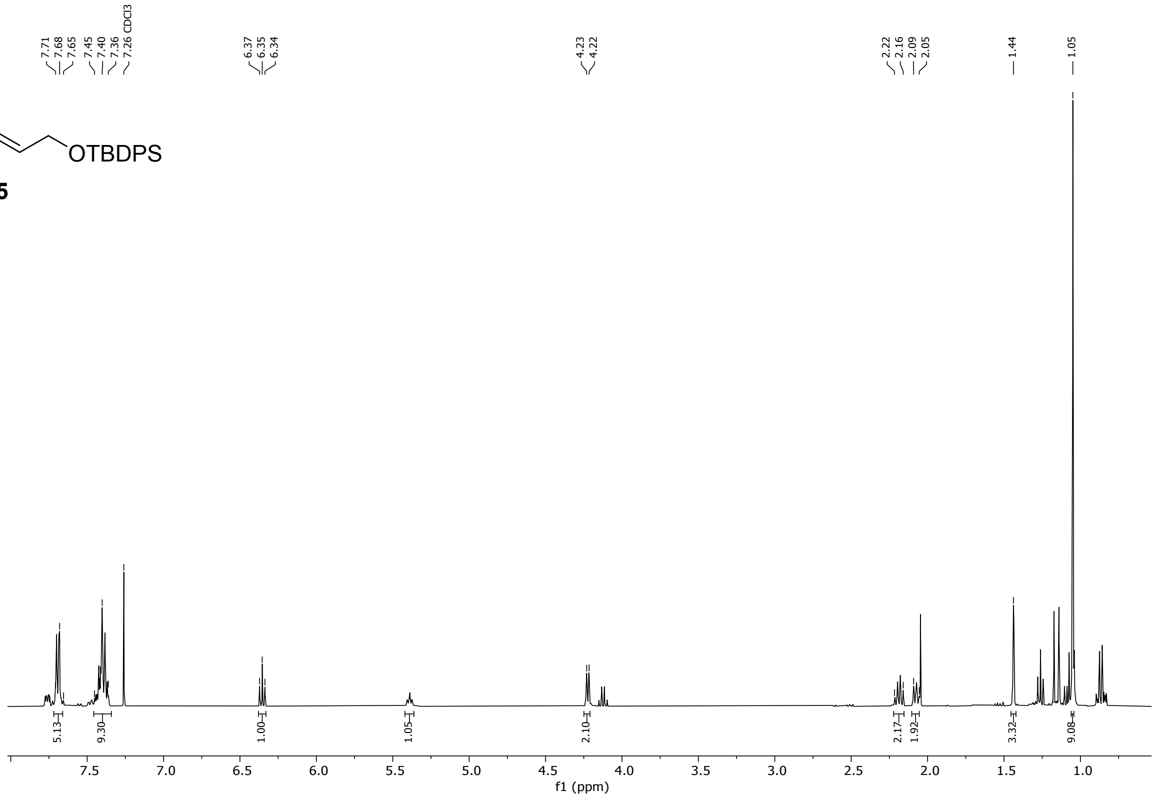

(E)-*tert*-Butyl((7,7-dibromo-3-methylhepta-2,6-dien-1-yl)oxy)diphenylsilane **15**, ^{13}C-NMR, 101 MHz, $CDCl_3$:

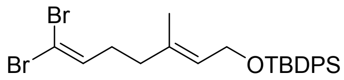

15

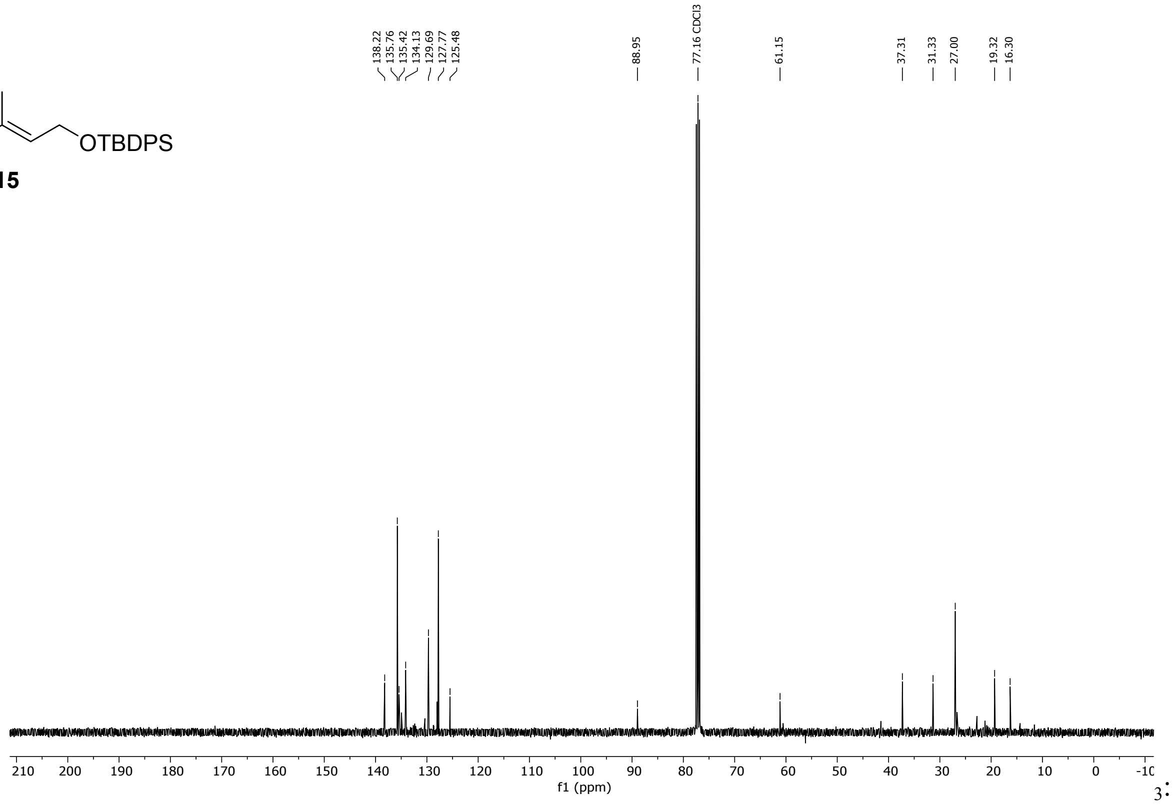

(*E*)-*tert*-Butyl((3-methylhept-2-en-6-yn-1-yl)oxy)diphenylsilane **S4**, ^{1}H-NMR, 400 MHz, $CDCl_3$:

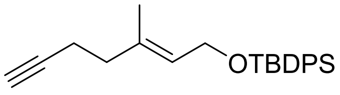

S4

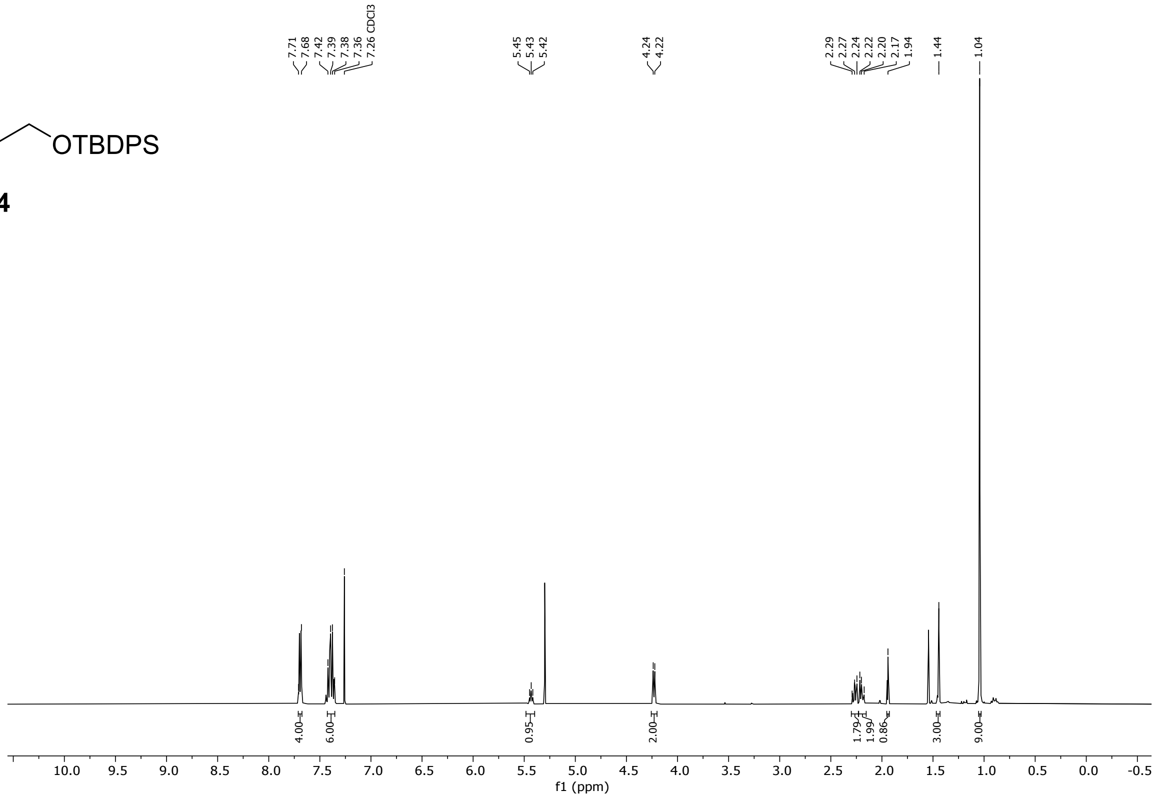

(*E*)-*tert*-Butyl((3-methylhept-2-en-6-yn-1-yl)oxy)diphenylsilane **S4**, ^{13}C-NMR, 101 MHz, $CDCl_3$:

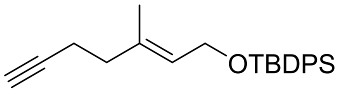

S4

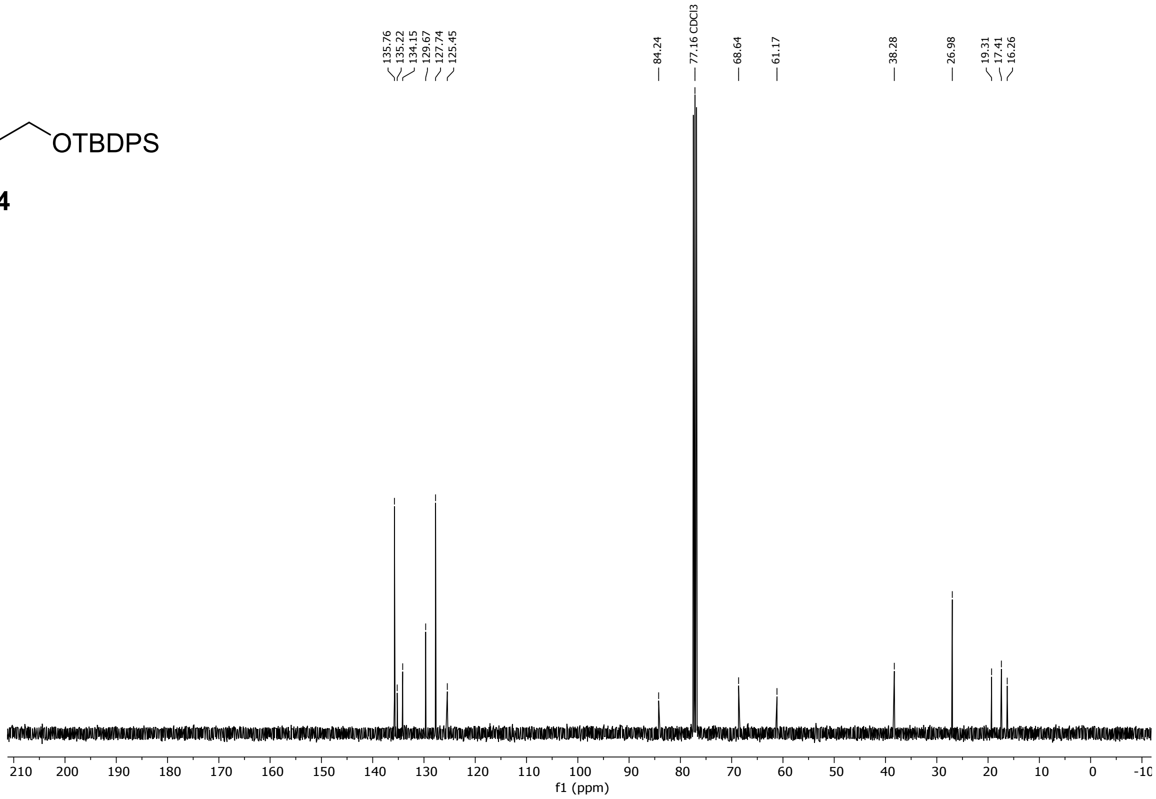

(*E*)-*tert*-Butyl((3-methyloct-2-en-6-yn-1-yl)oxy)diphenylsilane **16**, ^{1}H-NMR, 400 MHz, $CDCl_3$:

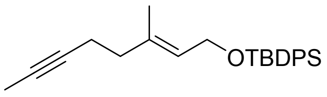

16

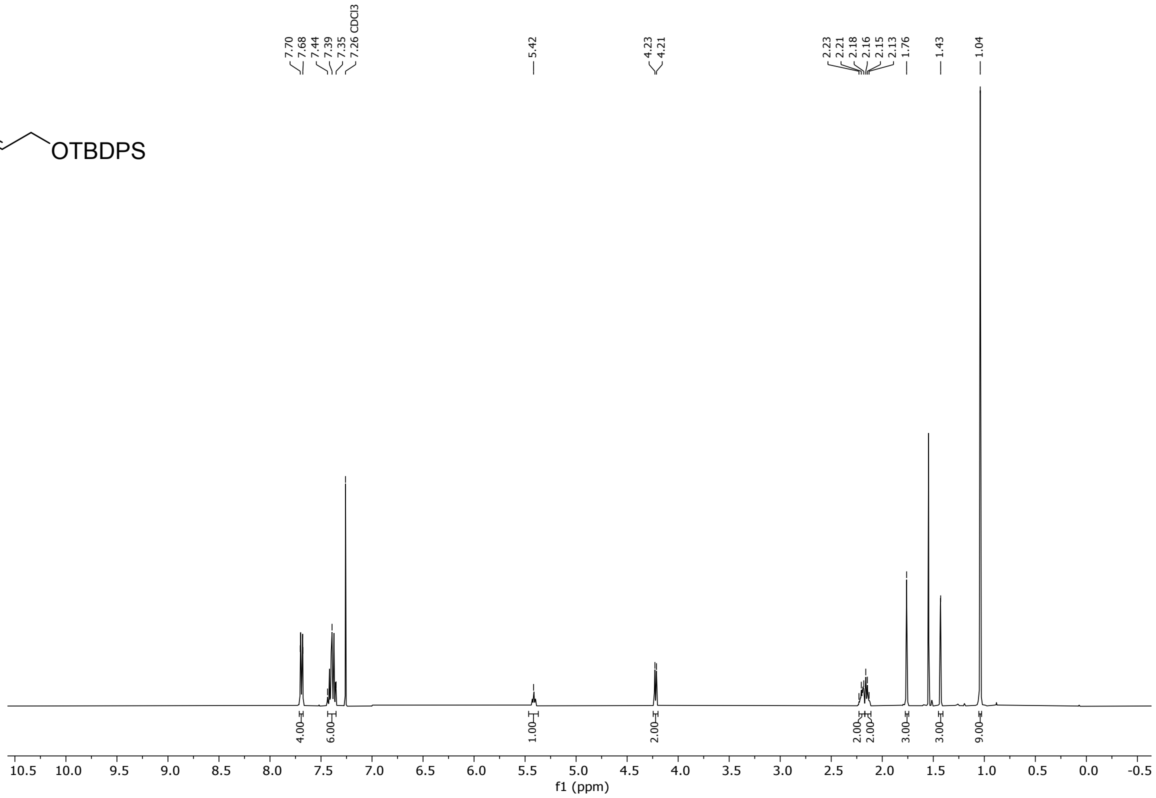

(*E*)-*tert*-Butyl((3-methyloct-2-en-6-yn-1-yl)oxy)diphenylsilane **16**, ^{13}C-NMR, 101 MHz, $CDCl_3$:

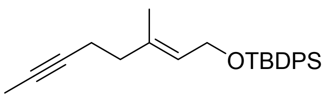

16

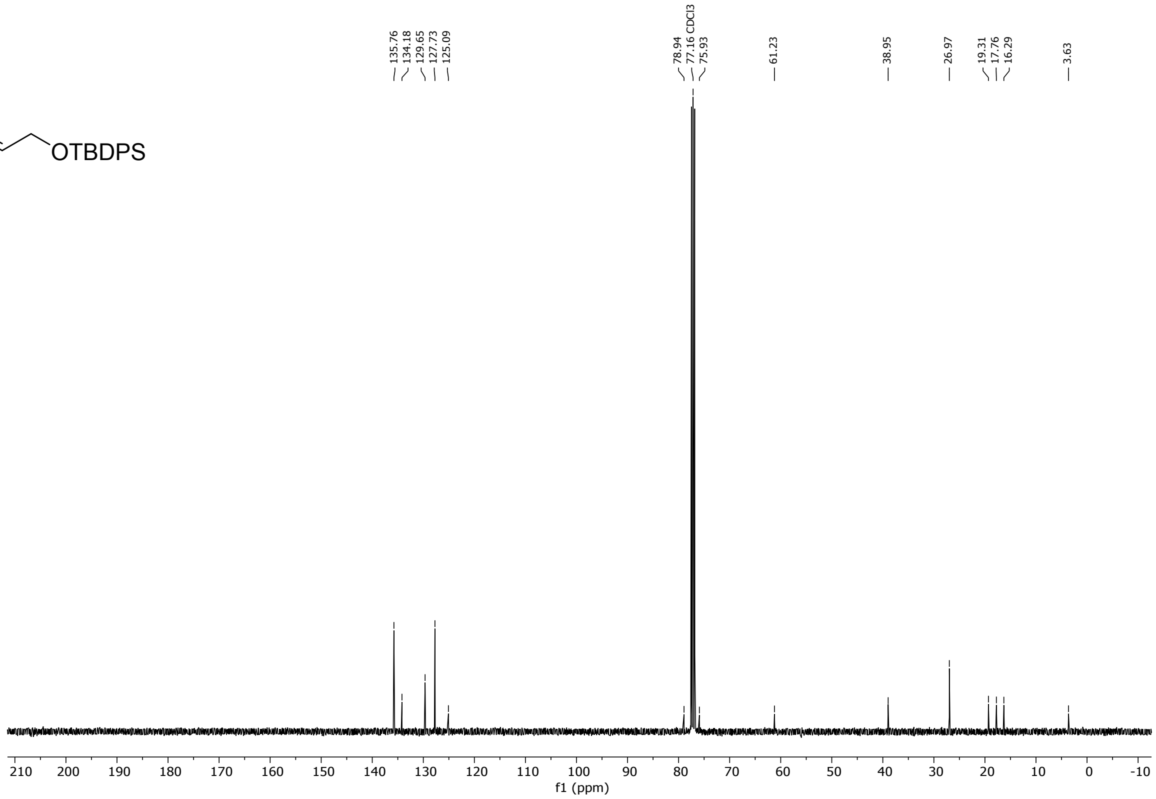

tert-Butyl-(((2E,6E)-7-iodo-3-methylocta-2,6-dien-1-yl)oxy)diphenylsilane **17**, ^{1}H-NMR, 400 MHz, $CDCl_3$:

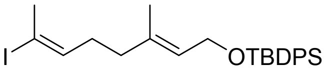

17

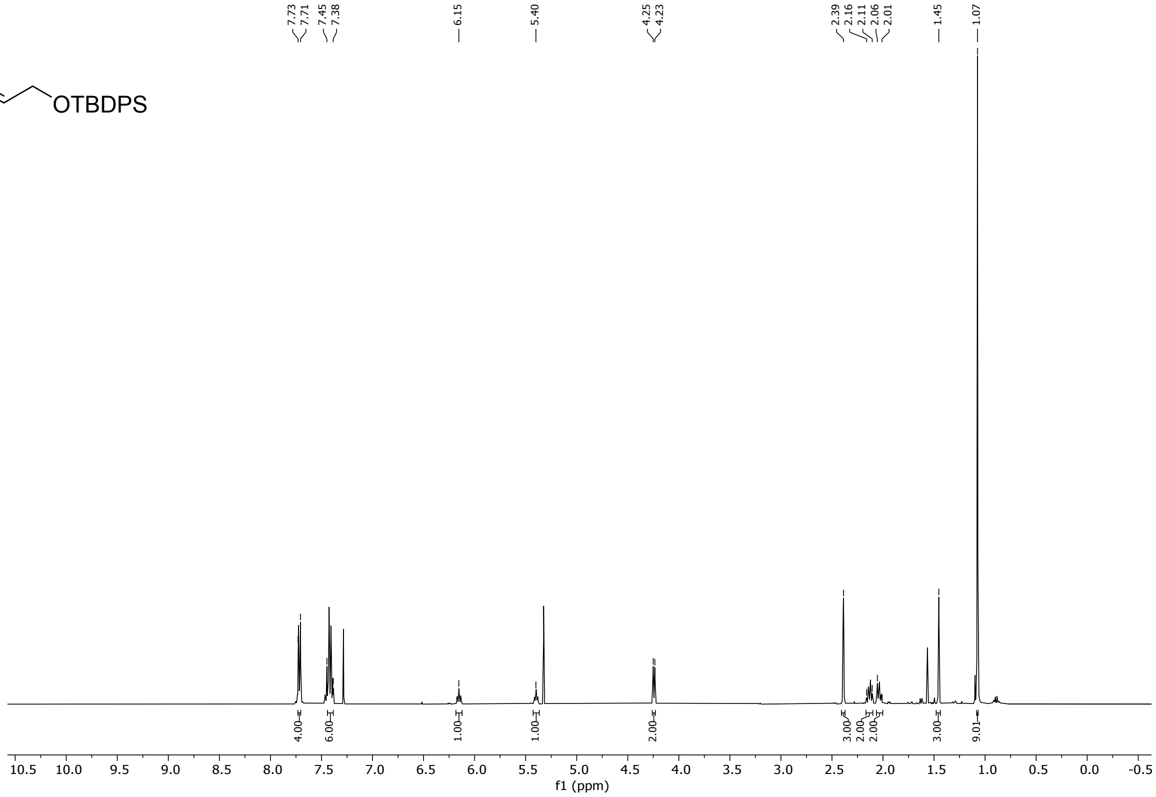

tert-Butyl-(((2E,6E)-7-iodo-3-methylocta-2,6-dien-1-yl)oxy)diphenylsilane **17**, ^{13}C-NMR, 101 MHz, $CDCl_3$:

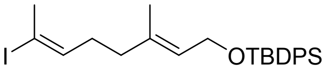

17

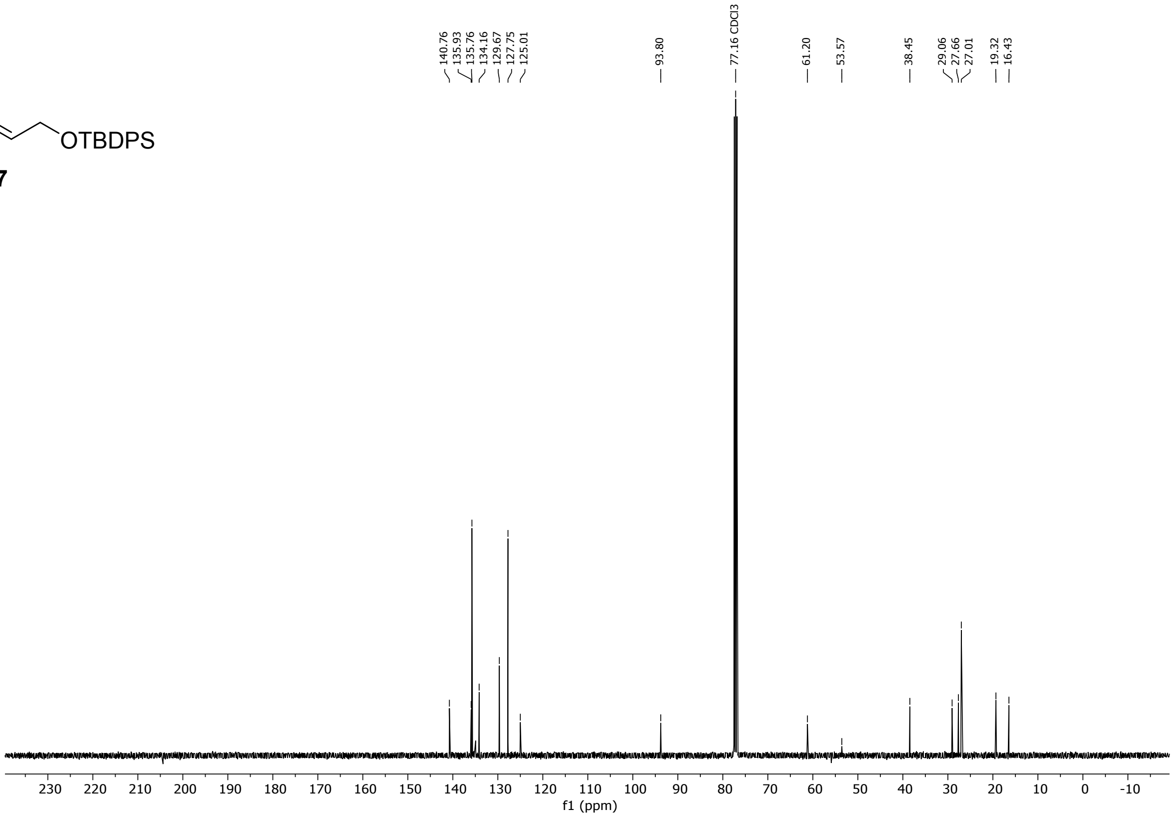

tert-Butyl-(((2*E*,6*E*)-3-methyl-7-((3-methylbut-2-en-1-yl)thio)octa-2,6-dien-1-yl)oxy)diphen-ylsilane **S5**, ^{1}H-NMR, 400 MHz, $CDCl_3$:

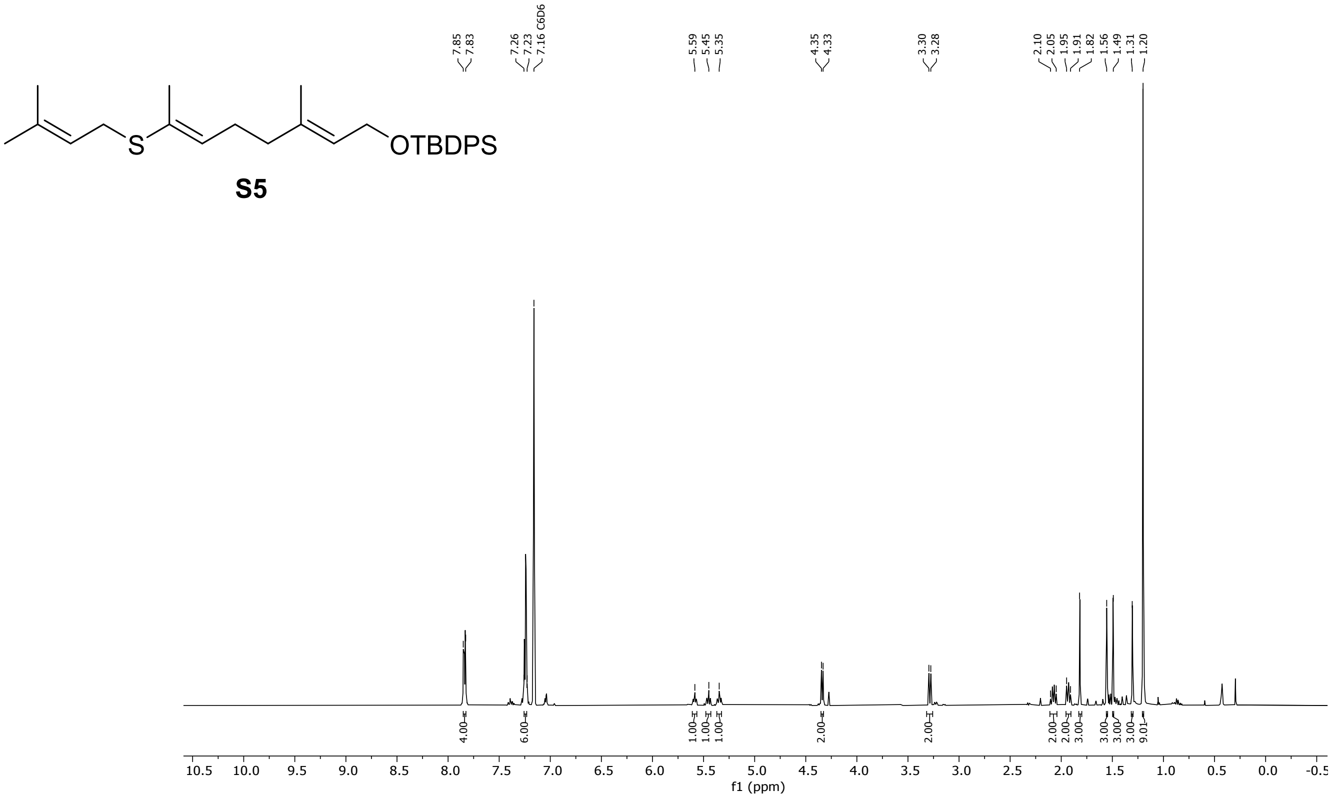

tert-Butyl-(((2*E*,6*E*)-3-methyl-7-((3-methylbut-2-en-1-yl)thio)octa-2,6-dien-1-yl)oxy)diphen-ylsilane **S5**, ^{13}C-NMR, 101 MHz, $CDCl_3$:

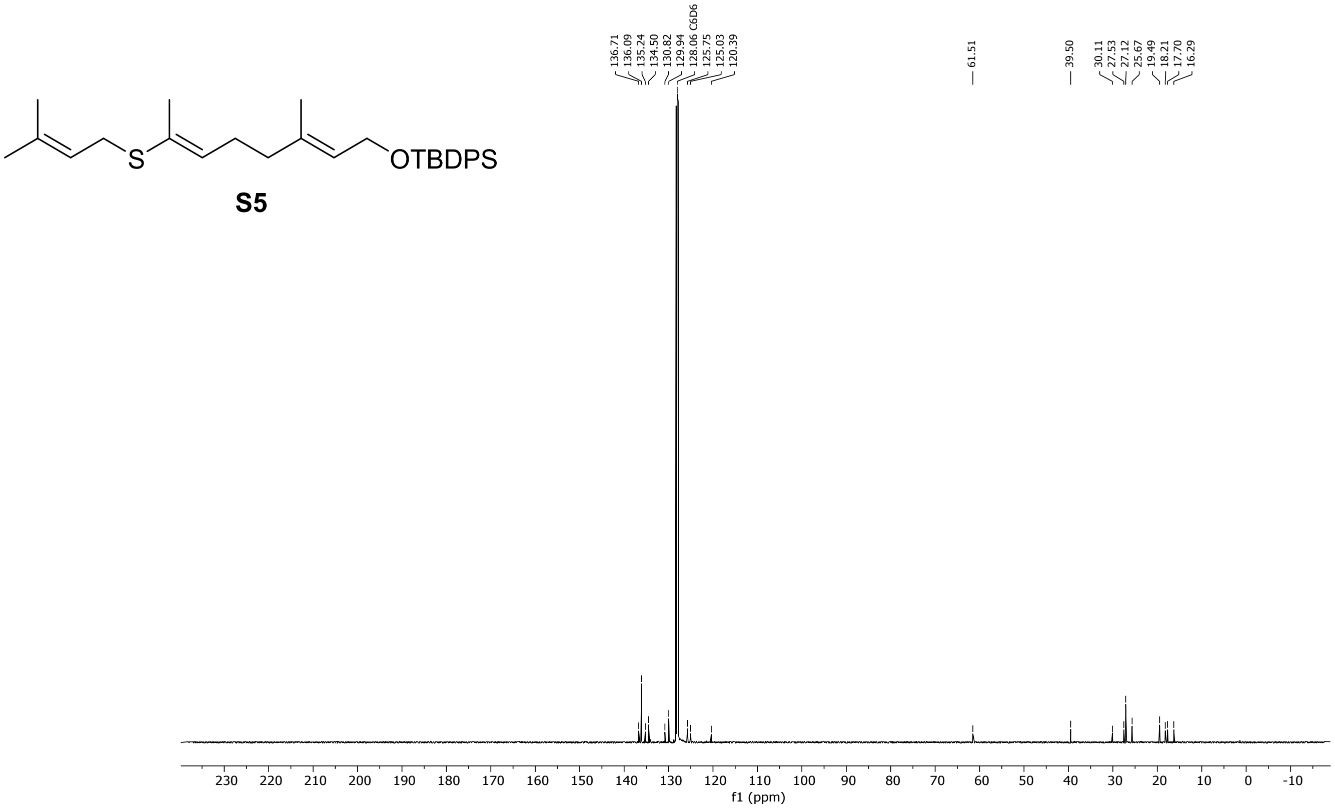

(6*E*,10*E*)-2,2,7,11,15,15,19-Heptamethyl-3,3-diphenyl-4-oxa-12,16-dithia-3-silaicosa-6,10,18-triene **22**, ^{1}H-NMR, 400 MHz, $CDCl_3$:

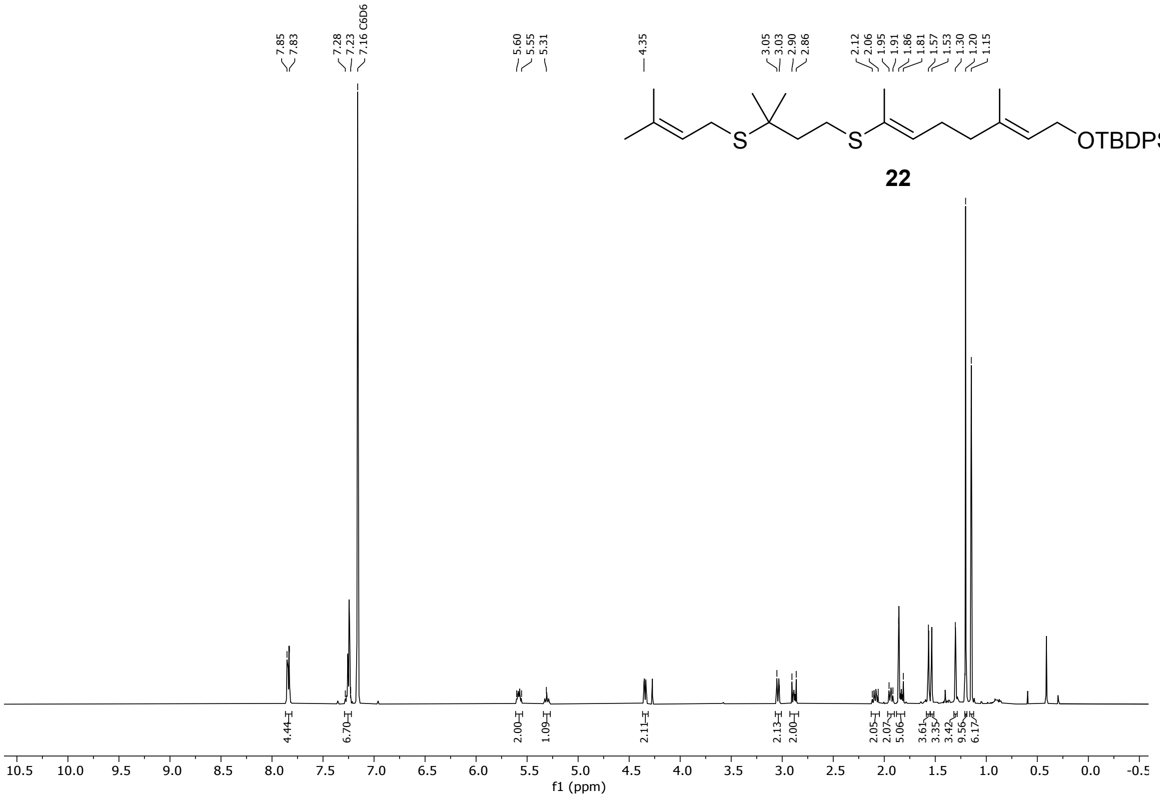

(6*E*,10*E*)-2,2,7,11,15,15,19-Heptamethyl-3,3-diphenyl-4-oxa-12,16-dithia-3-silaicosa-6,10,18-triene **22**, ^{13}C-NMR, 101 MHz, $CDCl_3$:

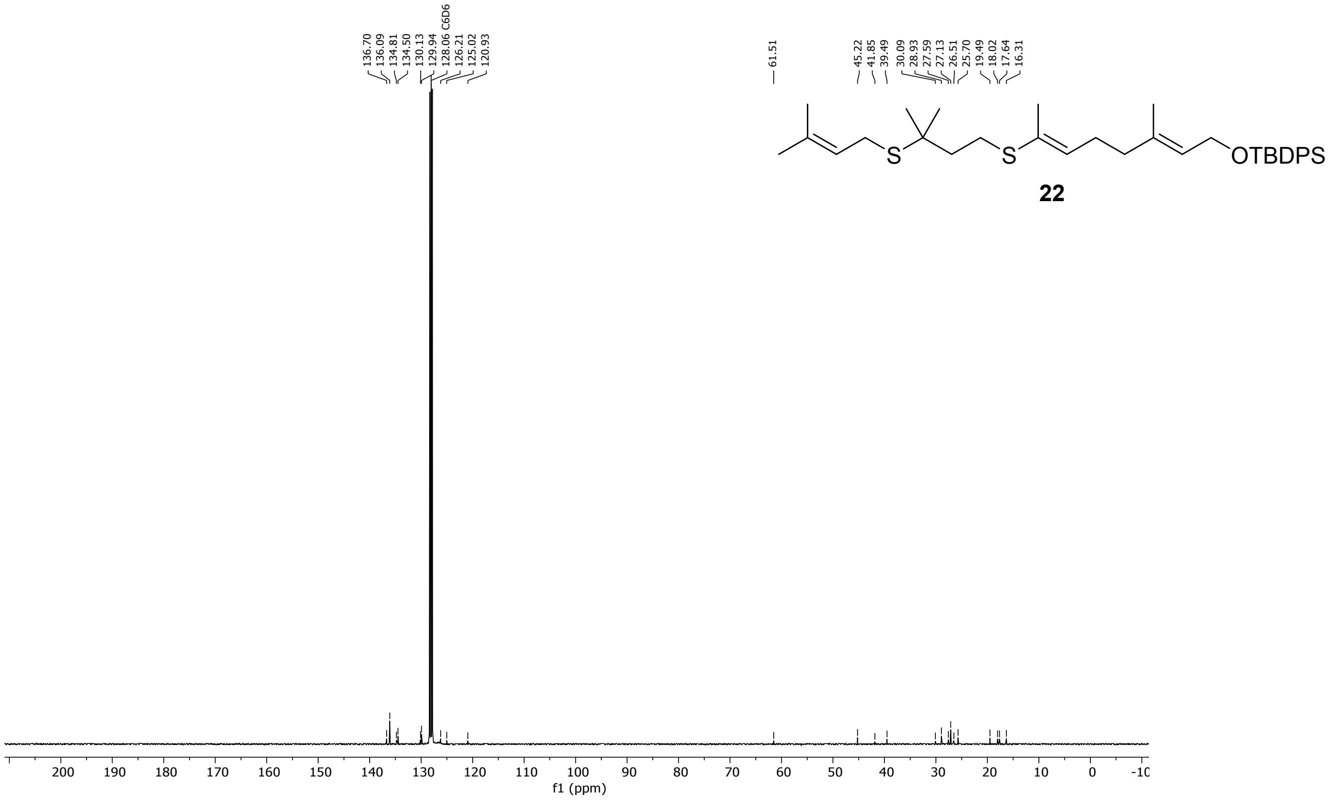

(2*E*,6*E*)-3-Methyl-7-((3-methylbut-2-en-1-yl)thio)octa-2,6-dien-1-ol **18**, ^{1}H-NMR, 400 MHz, $CDCl_3$:

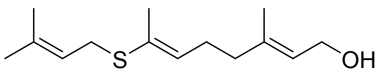

18

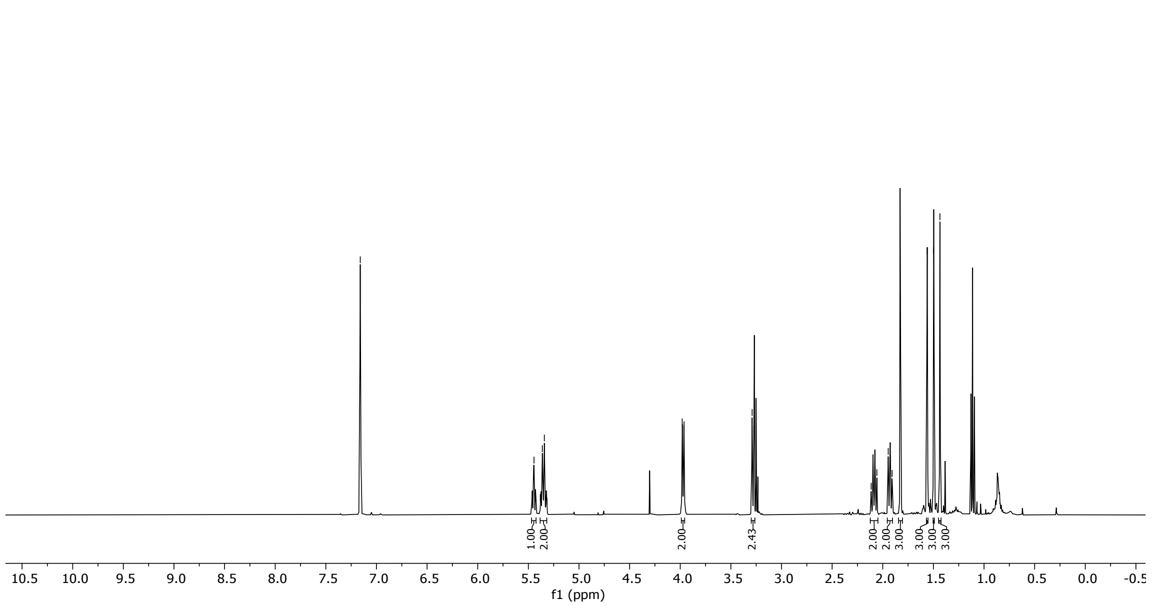

(2*E*,6*E*)-3-Methyl-7-((3-methylbut-2-en-1-yl)thio)octa-2,6-dien-1-ol **18**, ^{13}C-NMR, 101 MHz, $CDCl_3$:

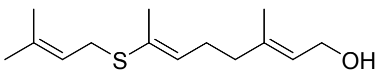

18

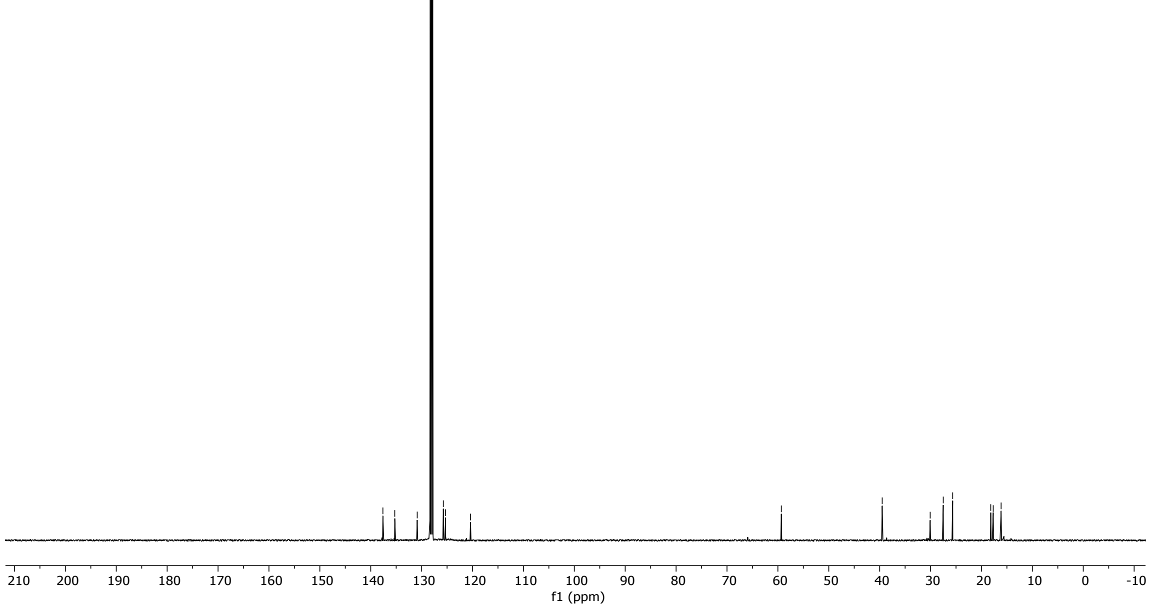

((2*E*,6*E*)-8-Chloro-6-methylocta-2,6-dien-2-yl)(3-methylbut-2-en-1-yl)sulfane **S6**, ^{1}H-NMR, 400 MHz, C_6D_6:

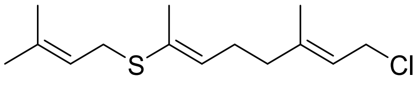

S6

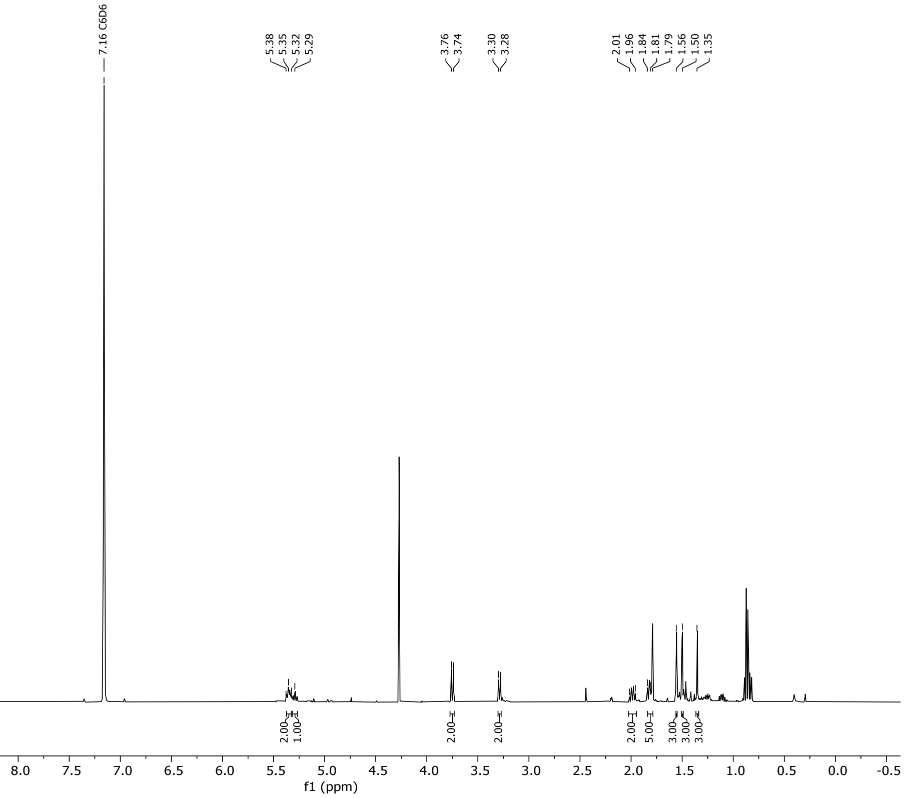

((2*E*,6*E*)-8-Chloro-6-methylocta-2,6-dien-2-yl)(3-methylbut-2-en-1-yl)sulfane **S6**, ^{13}C-NMR, 101 MHz, C_6D_6:

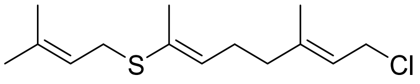

S6

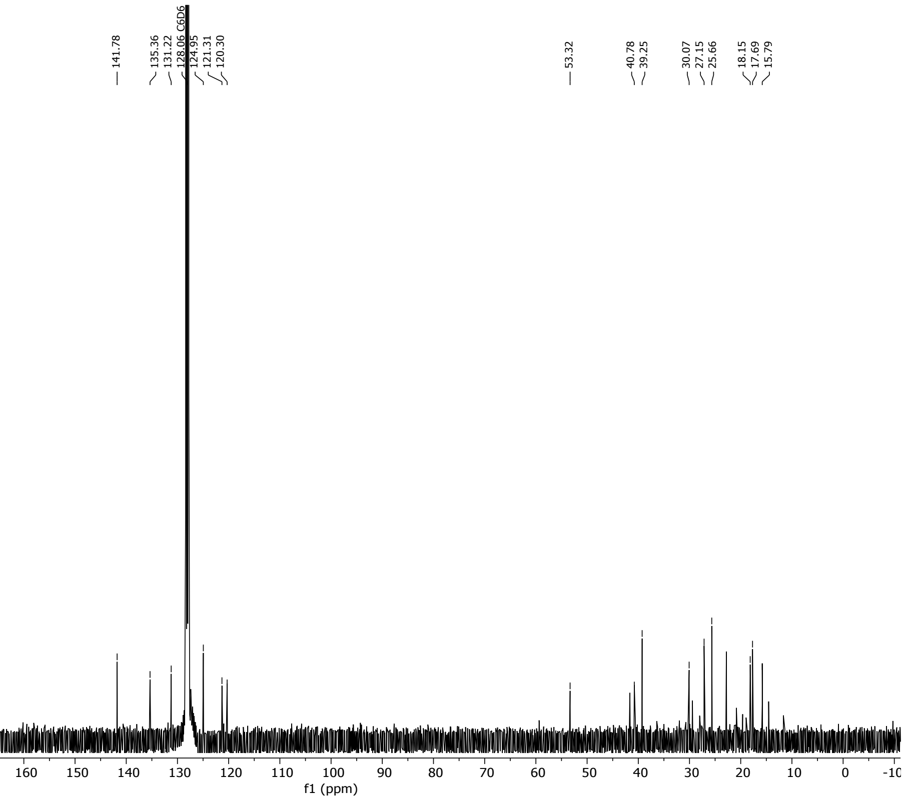

(2*E*,6*E*)-3-Methyl-7-((3-methylbut-2-en-1-yl)thio)octa-2,6-dien-1-yl trihydrogen diphosphate, triammonia salt **12**, ^{1}H-NMR, 400 MHz, D_2O:

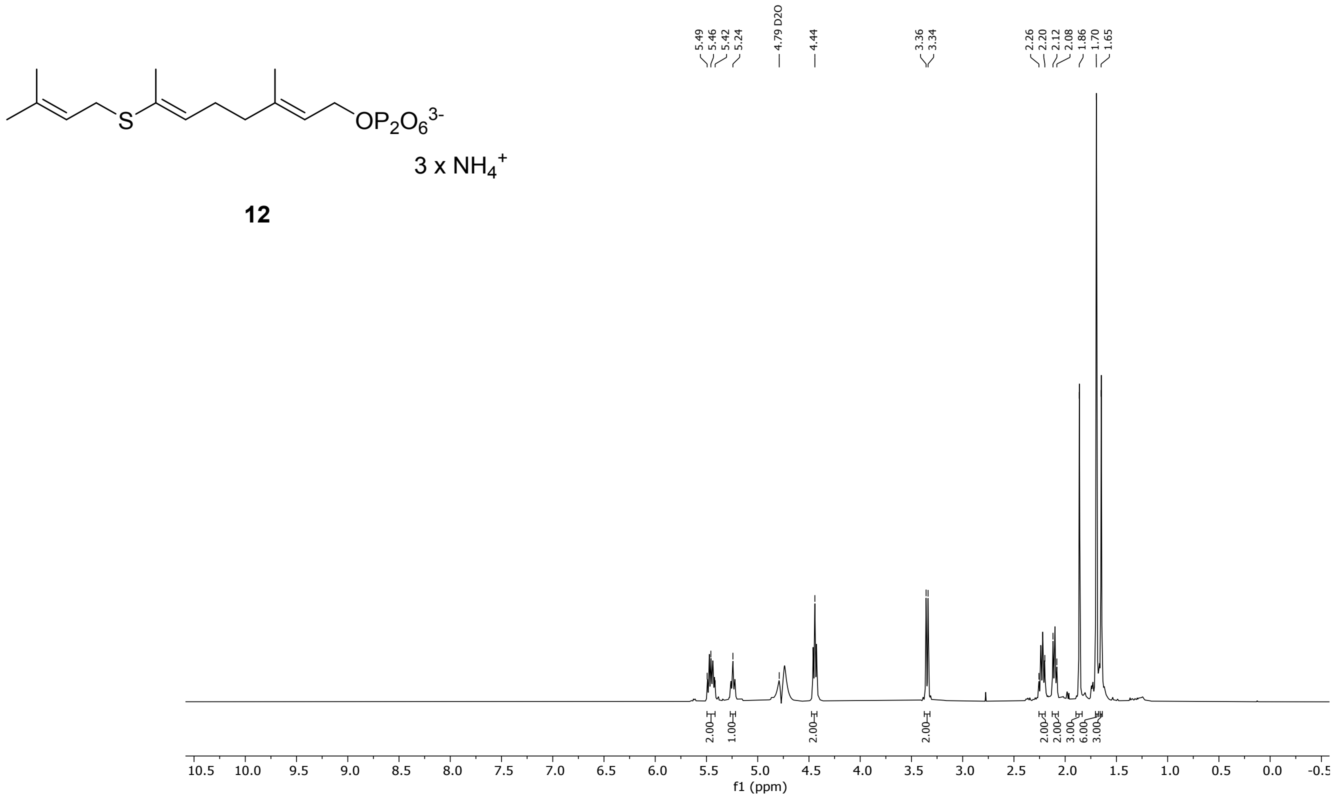

(2*E*,6*E*)-3-Methyl-7-((3-methylbut-2-en-1-yl)thio)octa-2,6-dien-1-yl trihydrogen diphosphate, triammonia salt **12**, ^{13}C-NMR, 101 MHz, D_2O:

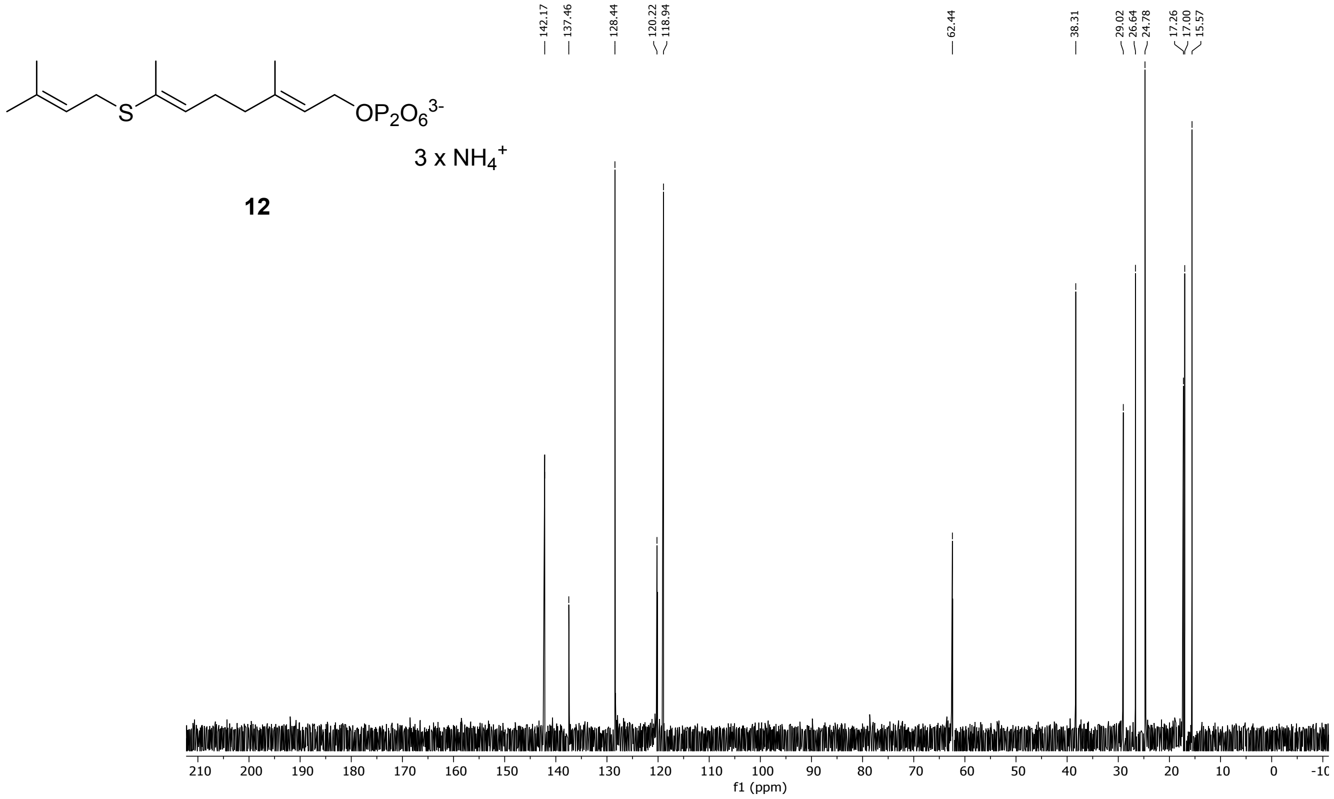

(2*E*,6*E*)-3-Methyl-7-((3-methylbut-2-en-1-yl)thio)octa-2,6-dien-1-yl trihydrogen diphosphate, triammonia salt **12**, ^{31}P-NMR, 162 MHz, D_2O:

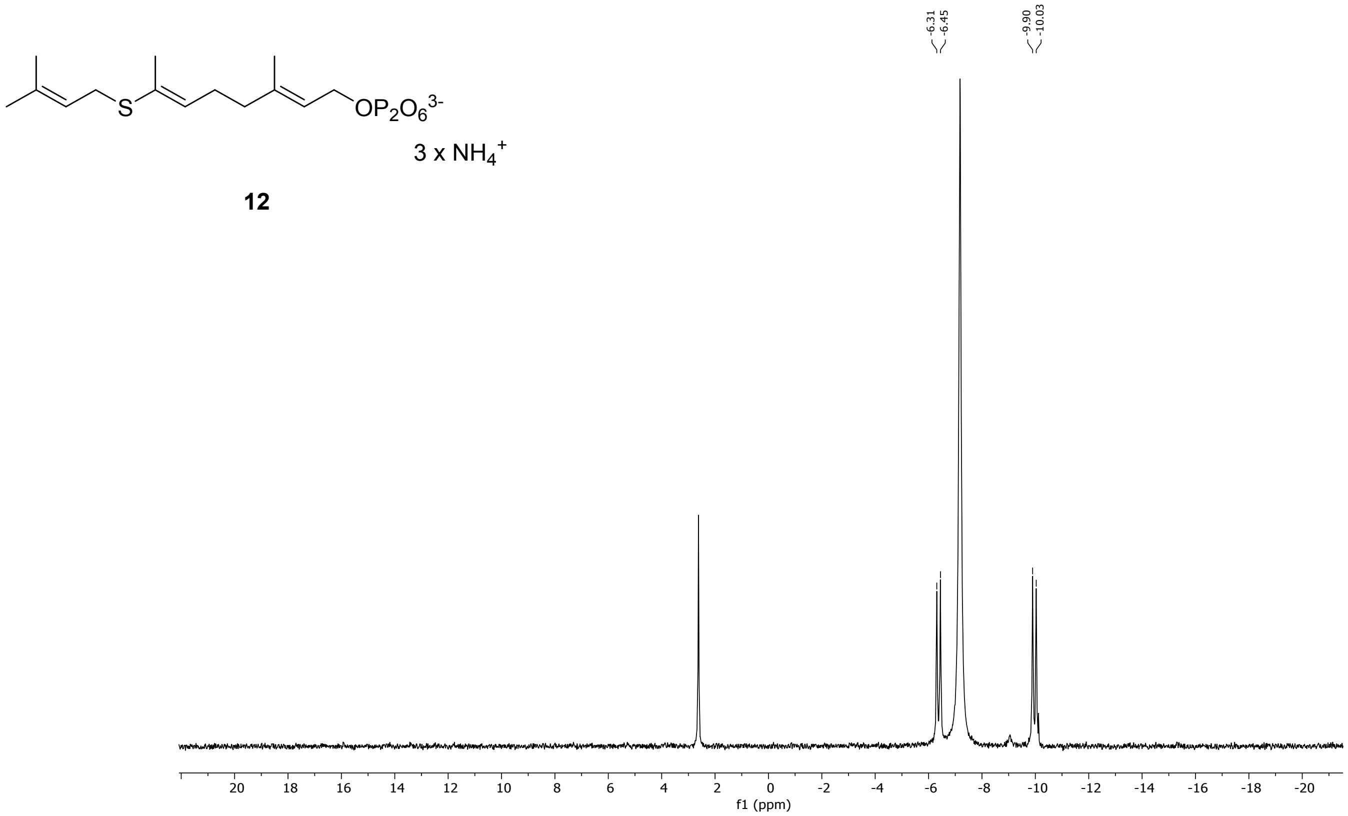

6. GC-MS data of the new terpenoids

Biotransformation of thio-FPP **12** with PenA (pH 7.6):

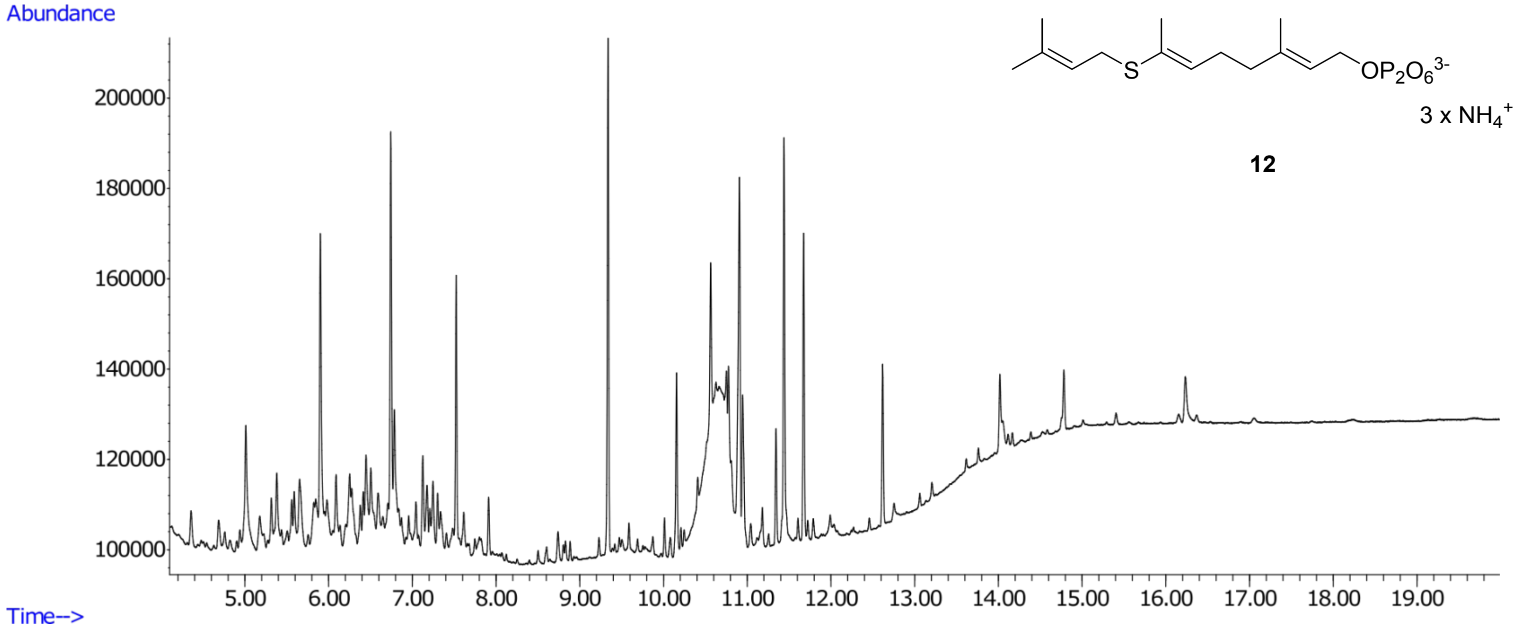

Biotransformation of thio-FPP **12** with GcoA (pH 7.6):

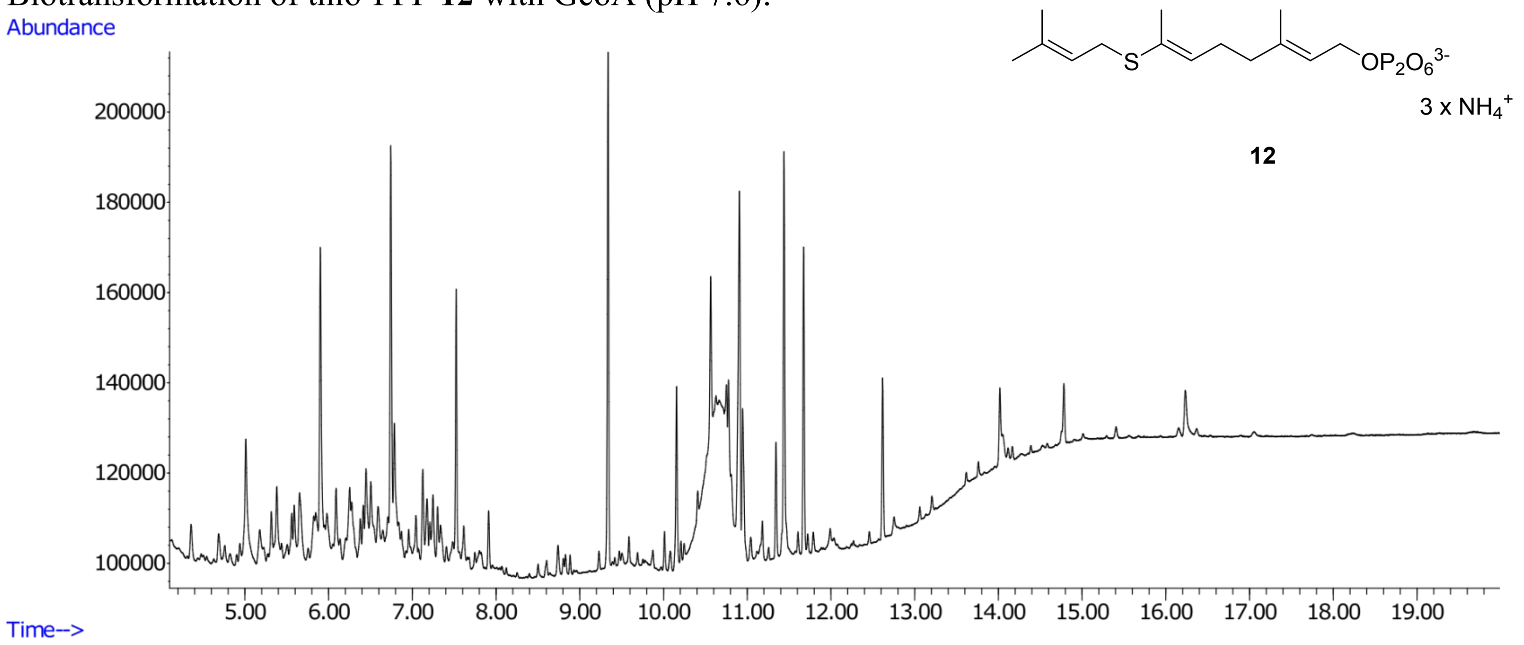

Biotransformation of thio-FPP **12** with Omp7 (pH 7.6):

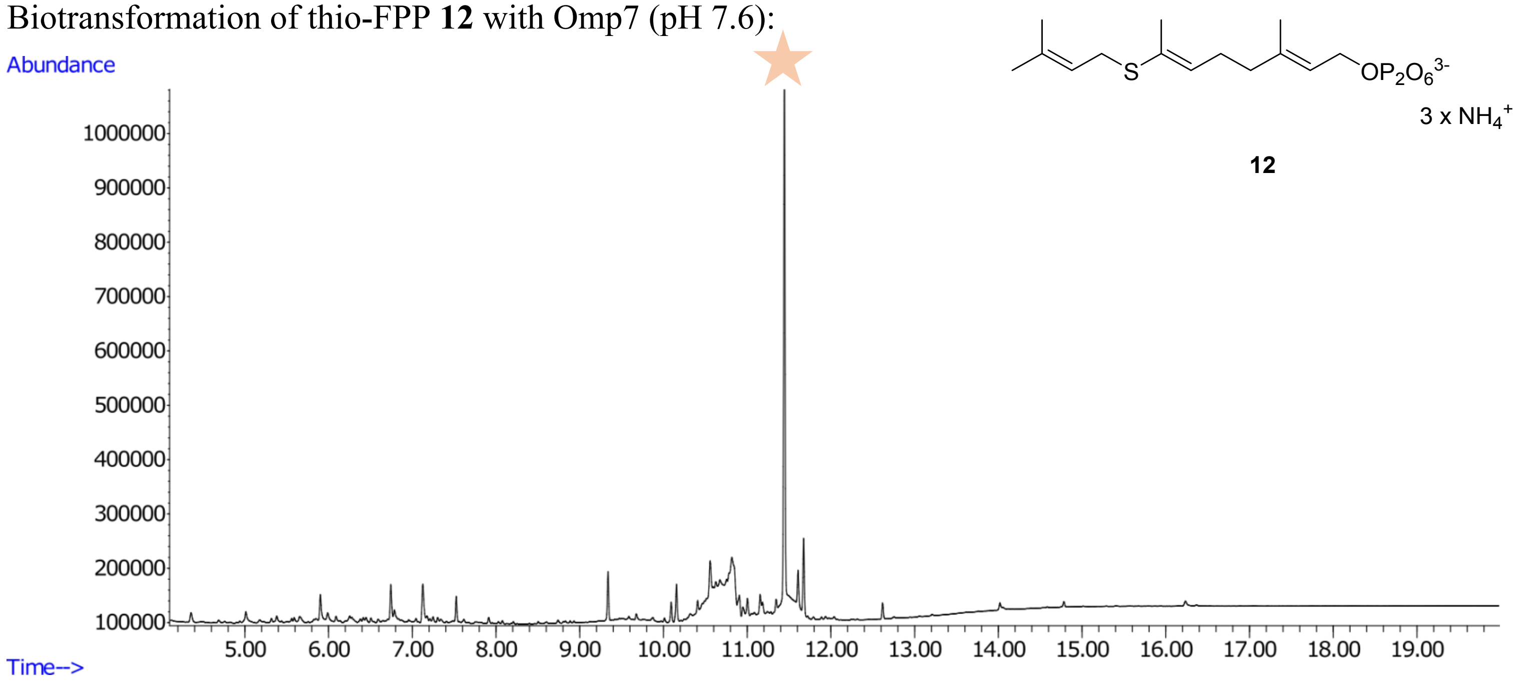

12

Biotransformation of thio-FPP **12** with Cop4 (pH 7.6):

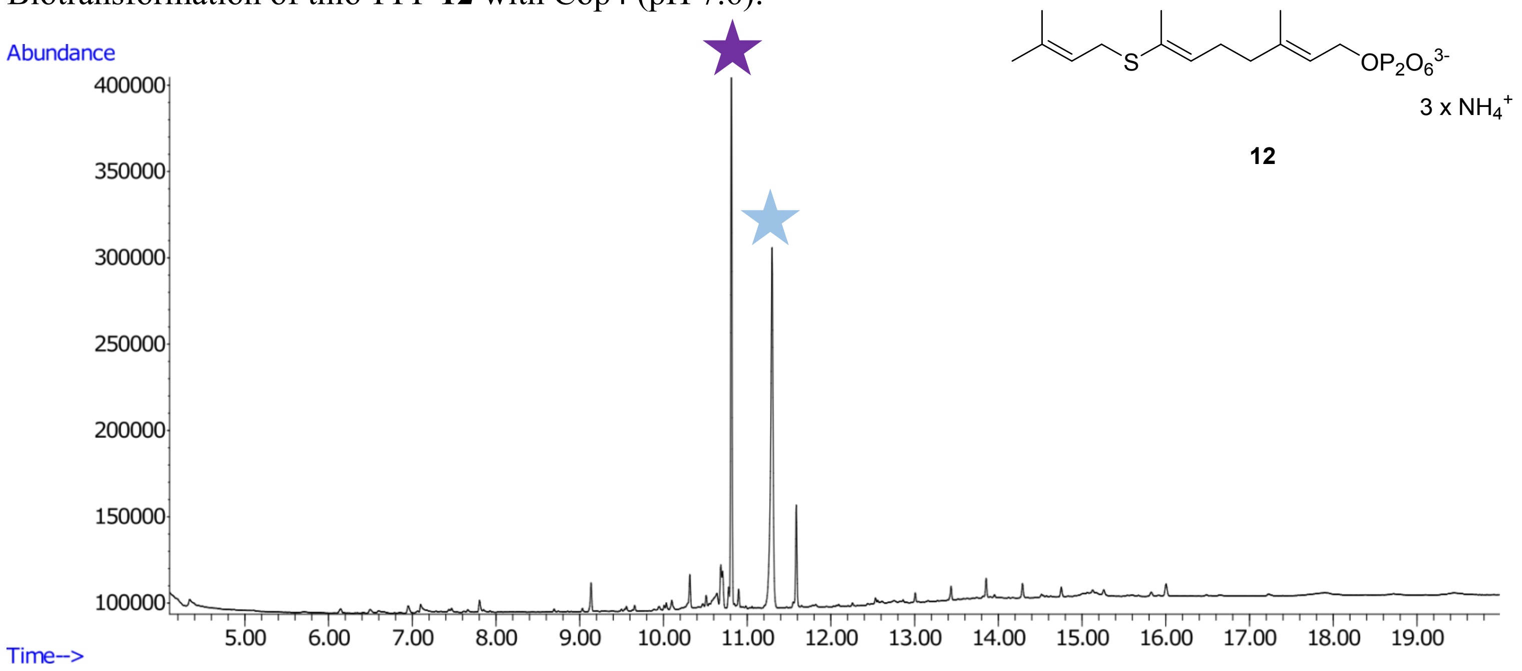

12

Biotransformation of thio-FPP **12** with BcBOT2 (pH 7.6):

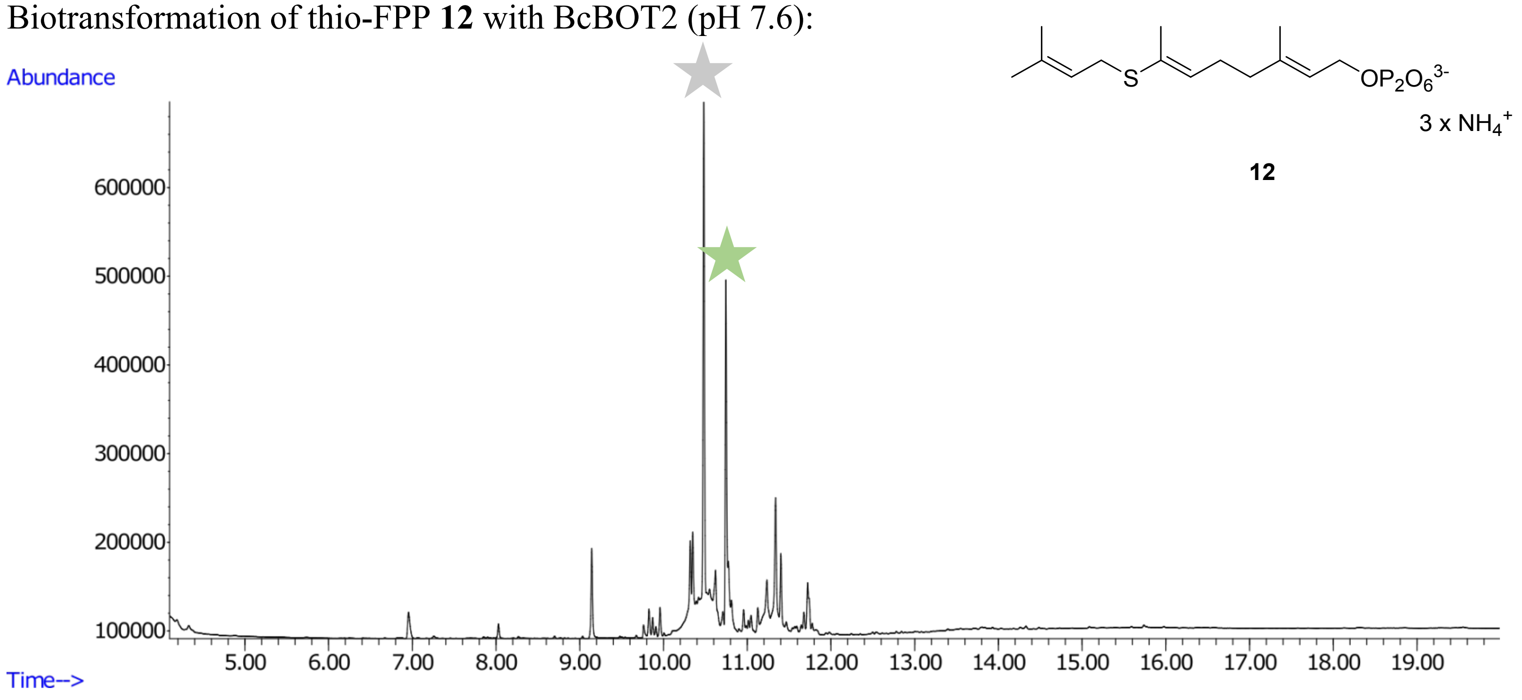

Mass spectra of δ-3-Thiocadinene **23**:

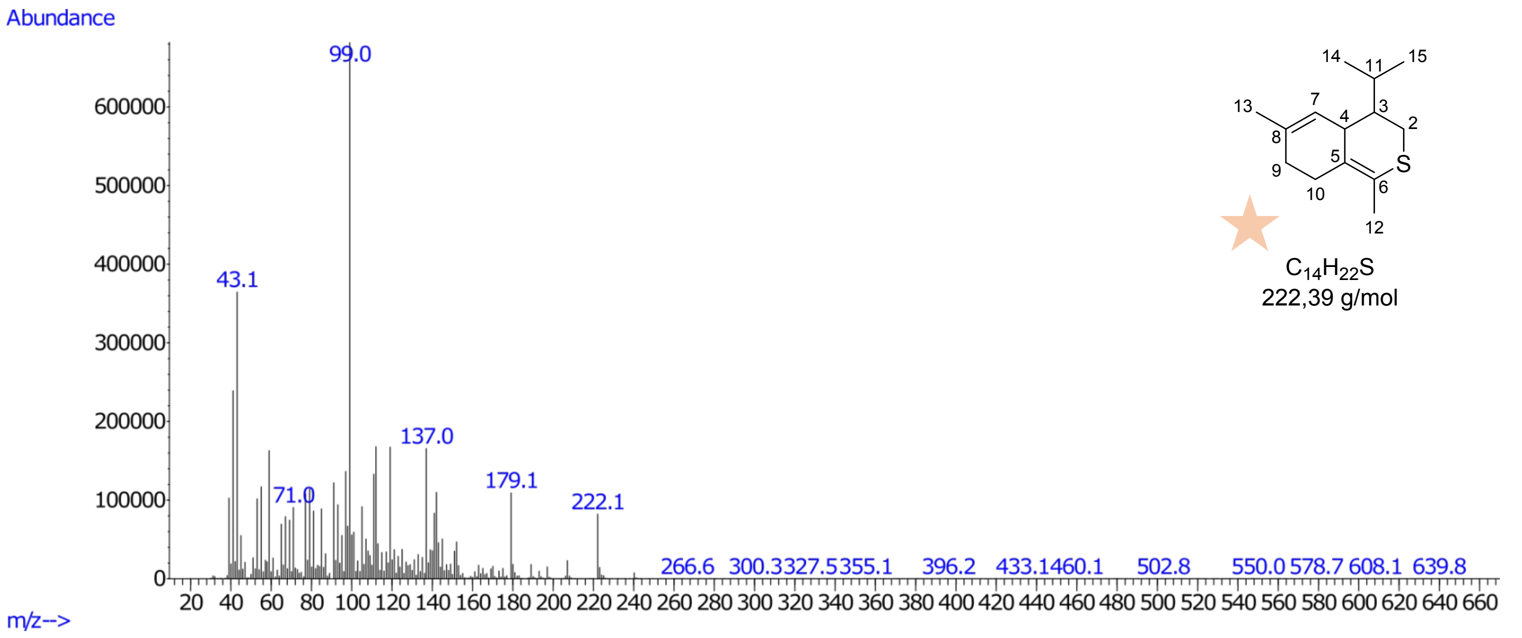

Mass spectra of δ-3-Thiocadinol **24**:

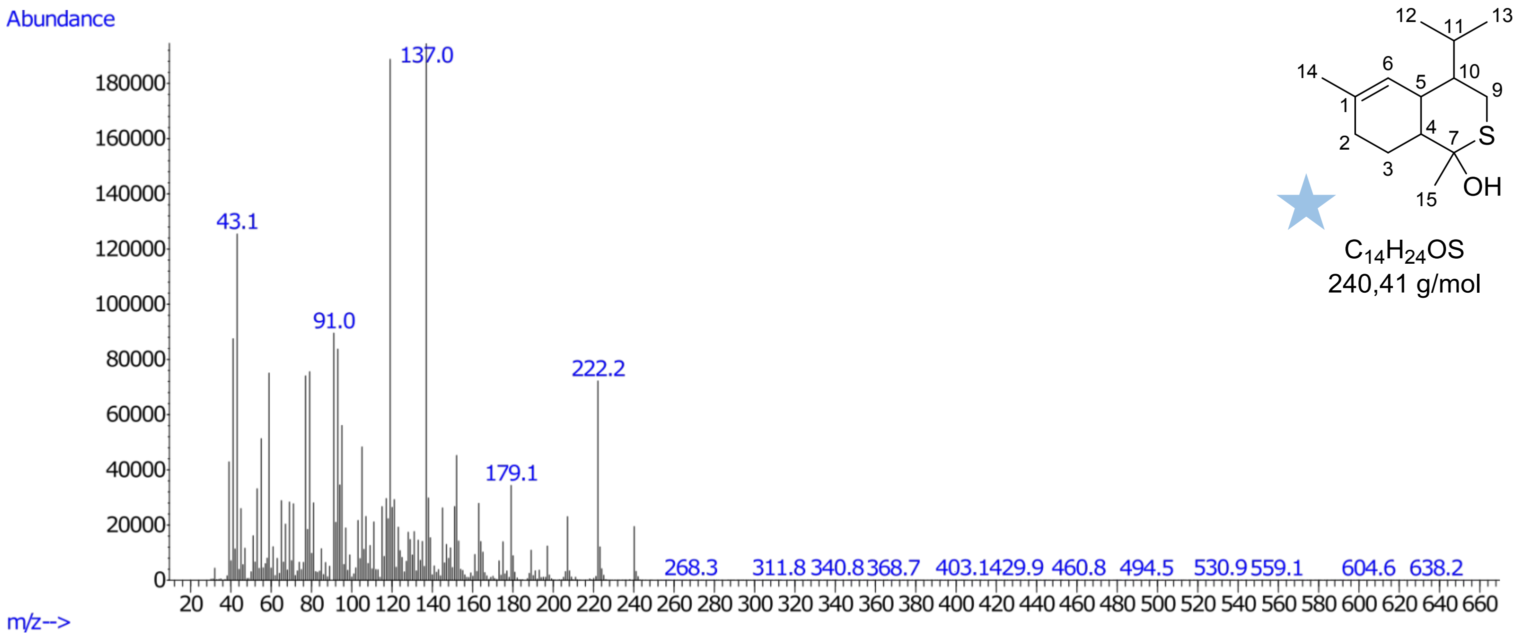

Mass spectra of Ketothiol **27**:

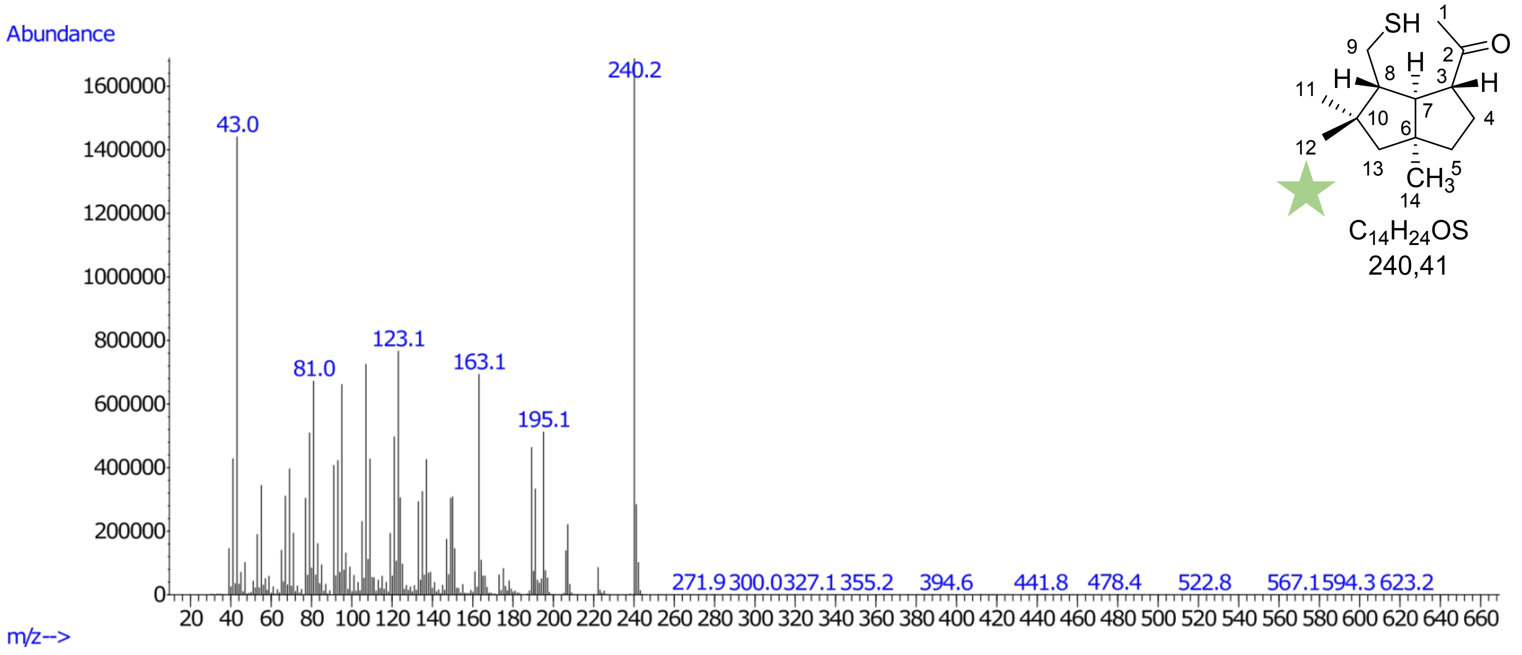

Mass spectra of the main product (could not isolated, RI: 1699) from the biotransformation of derivative **12** with BcBOT2:

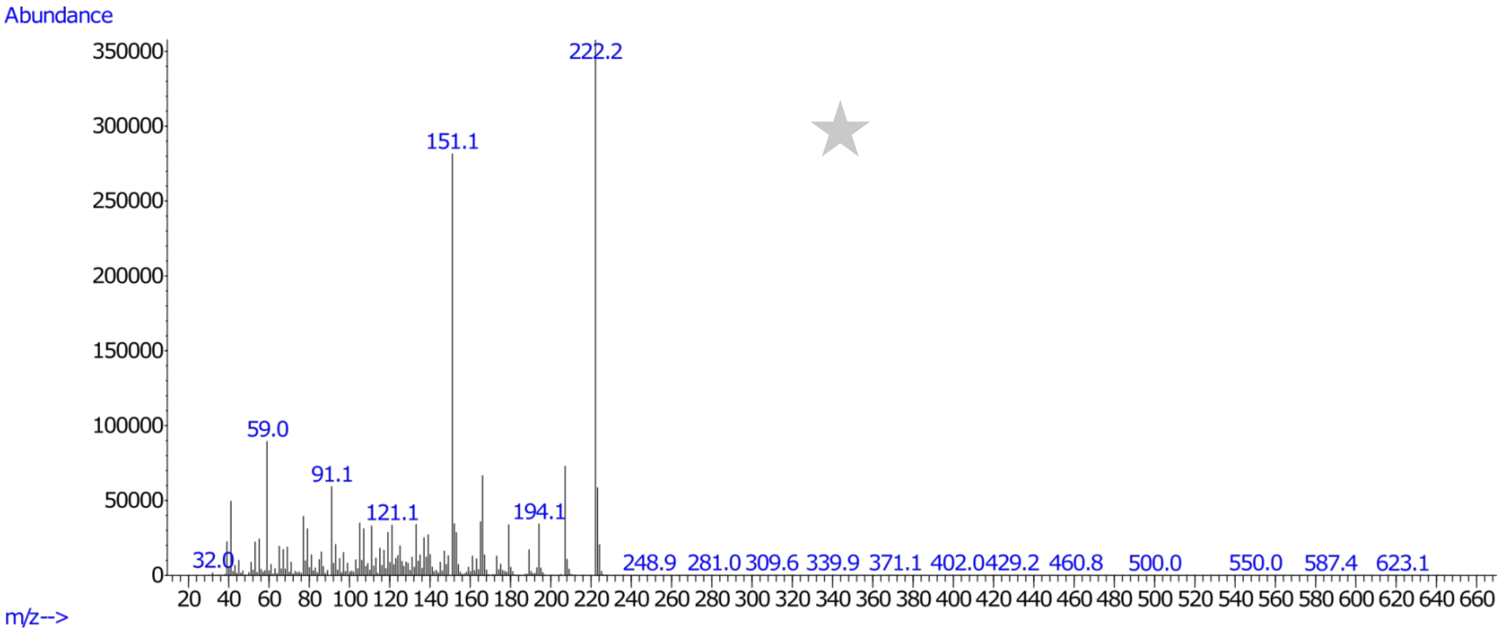

Mass spectra of the main product (could not isolated, RI: 1750) from the biotransformation of derivative **12** with Cop4:

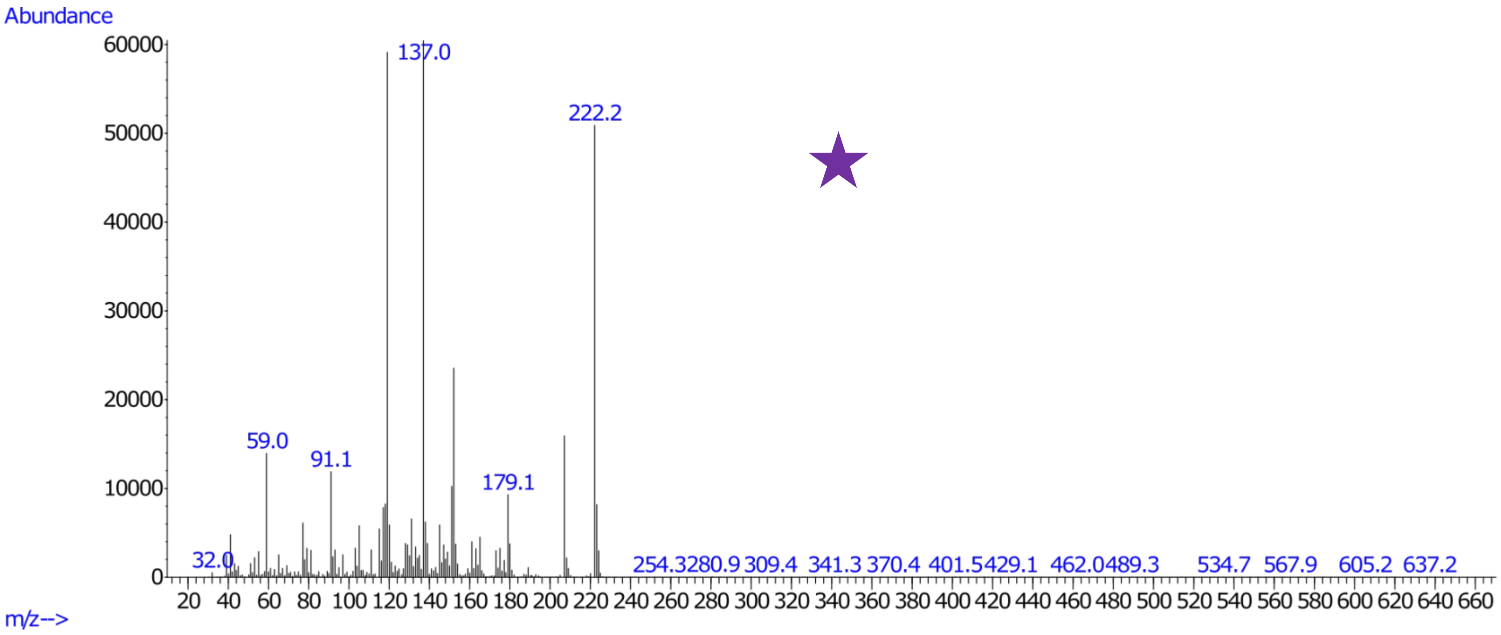

Negative control of every enzyme (biotransformation conditions without any substrate present):

Negative control of **PenA**:

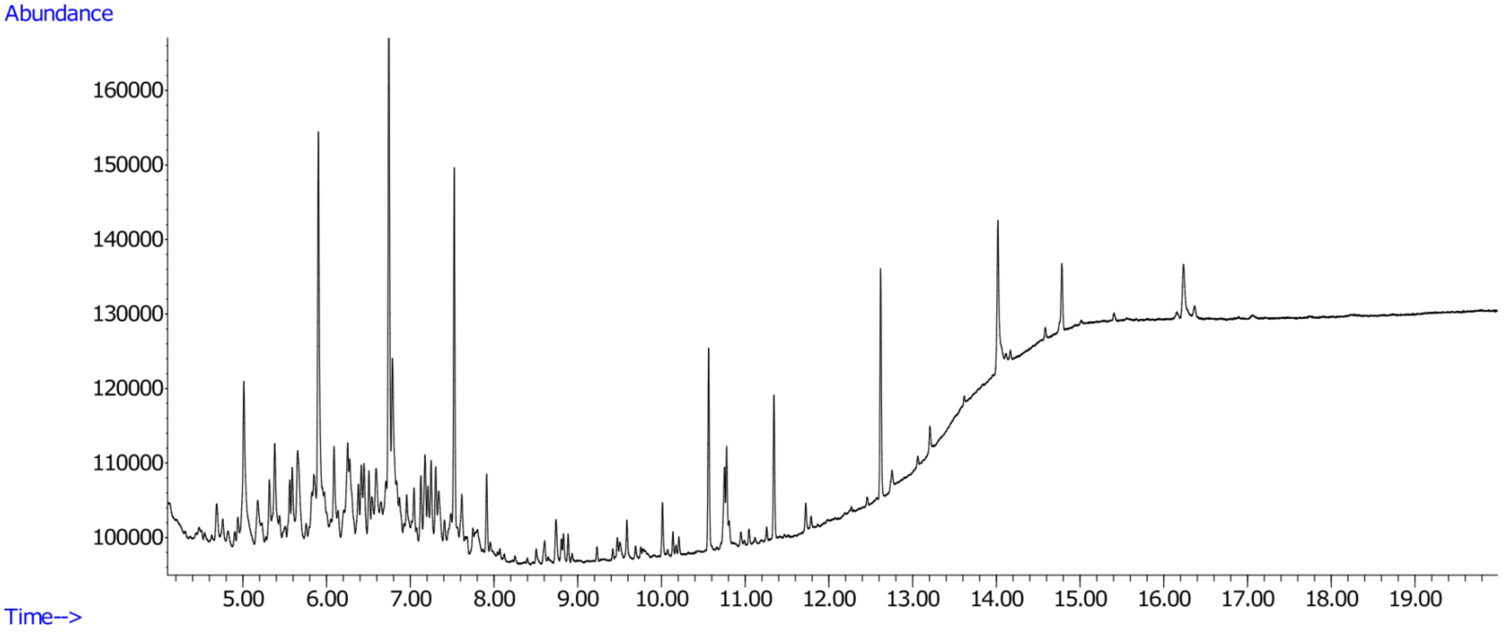

Negative control of **GcoA**:

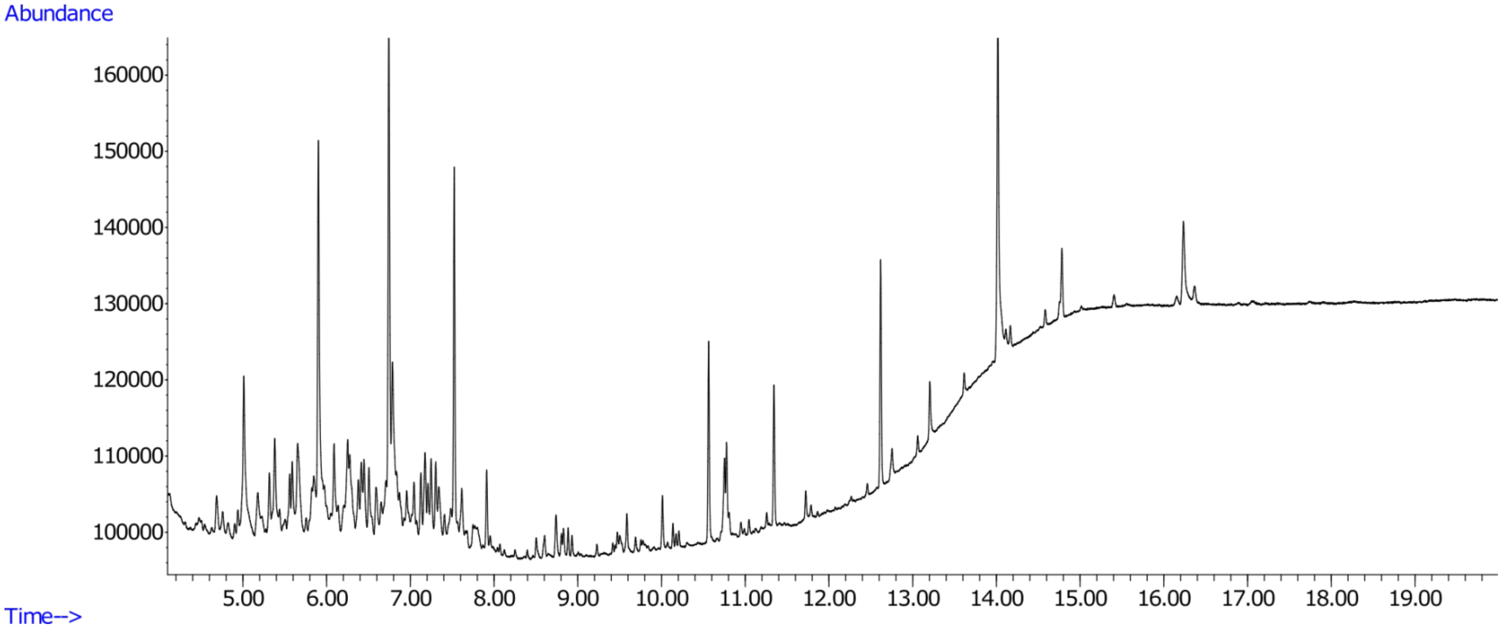

Negative control of **Omp7:**

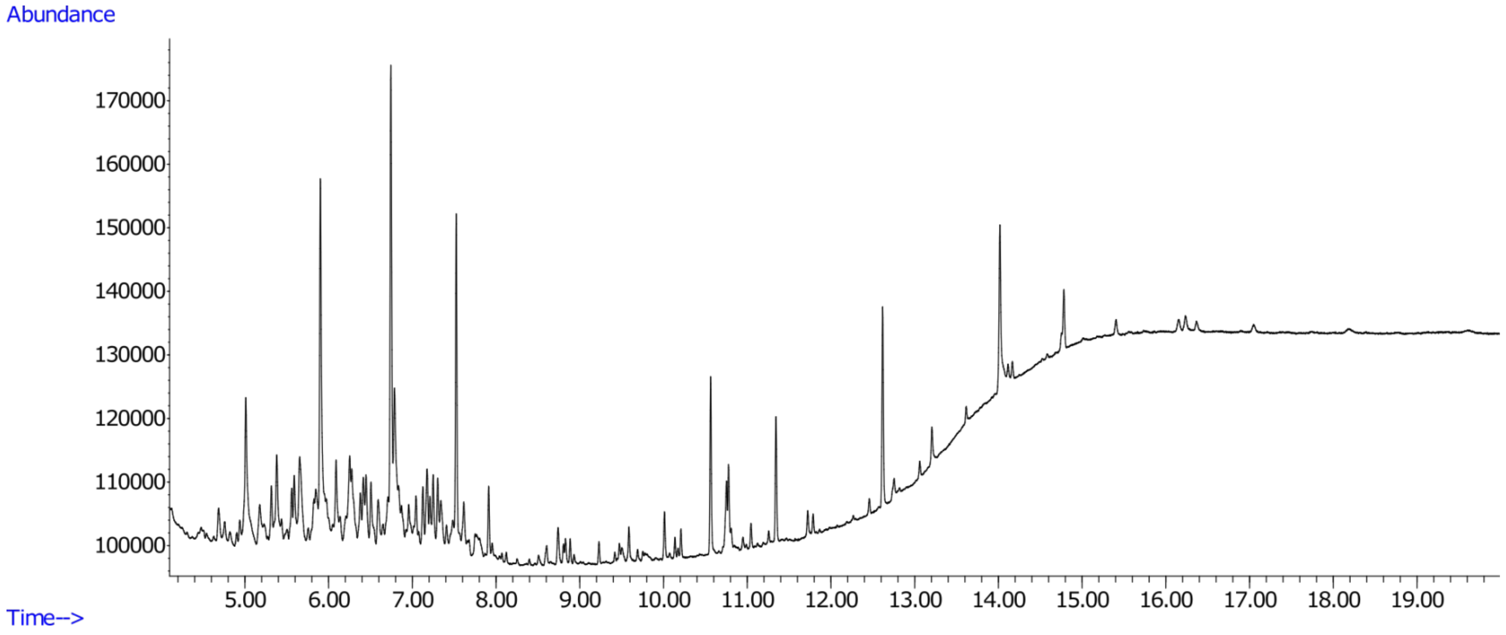

Negative control of **Cop4:**

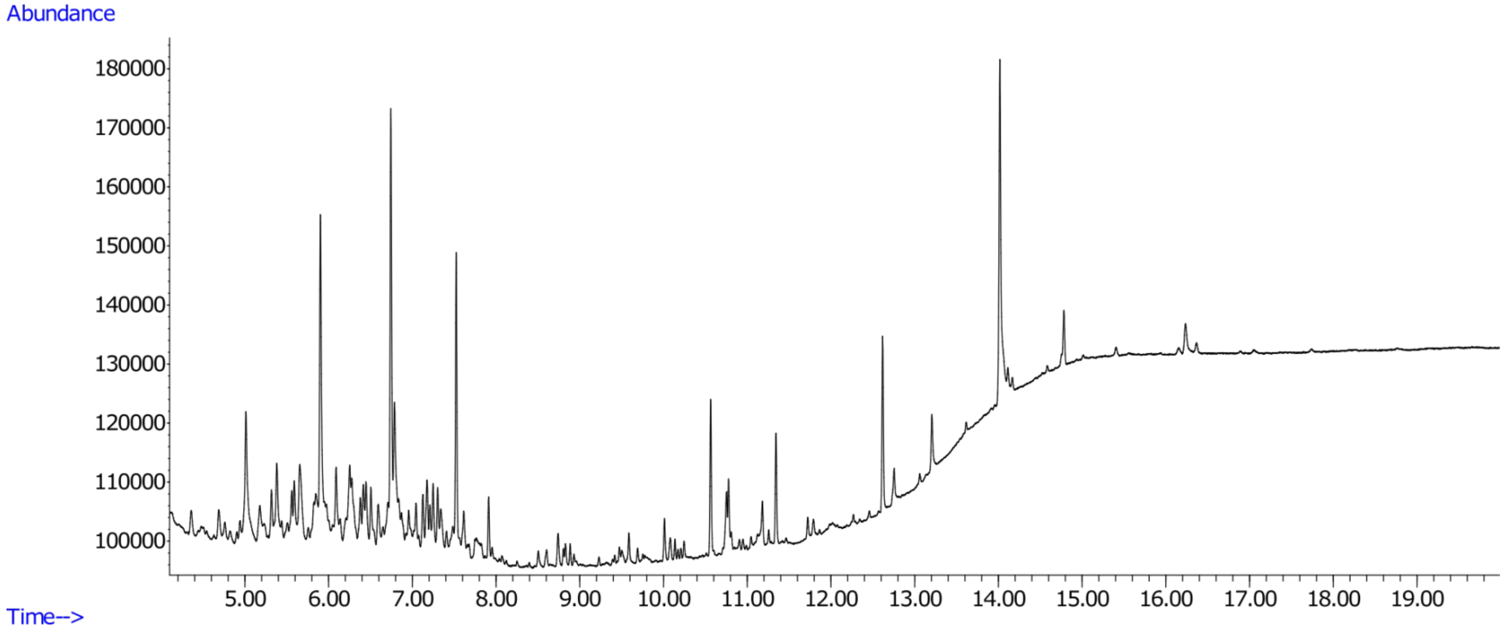

Negative control of **BcBOT2:**

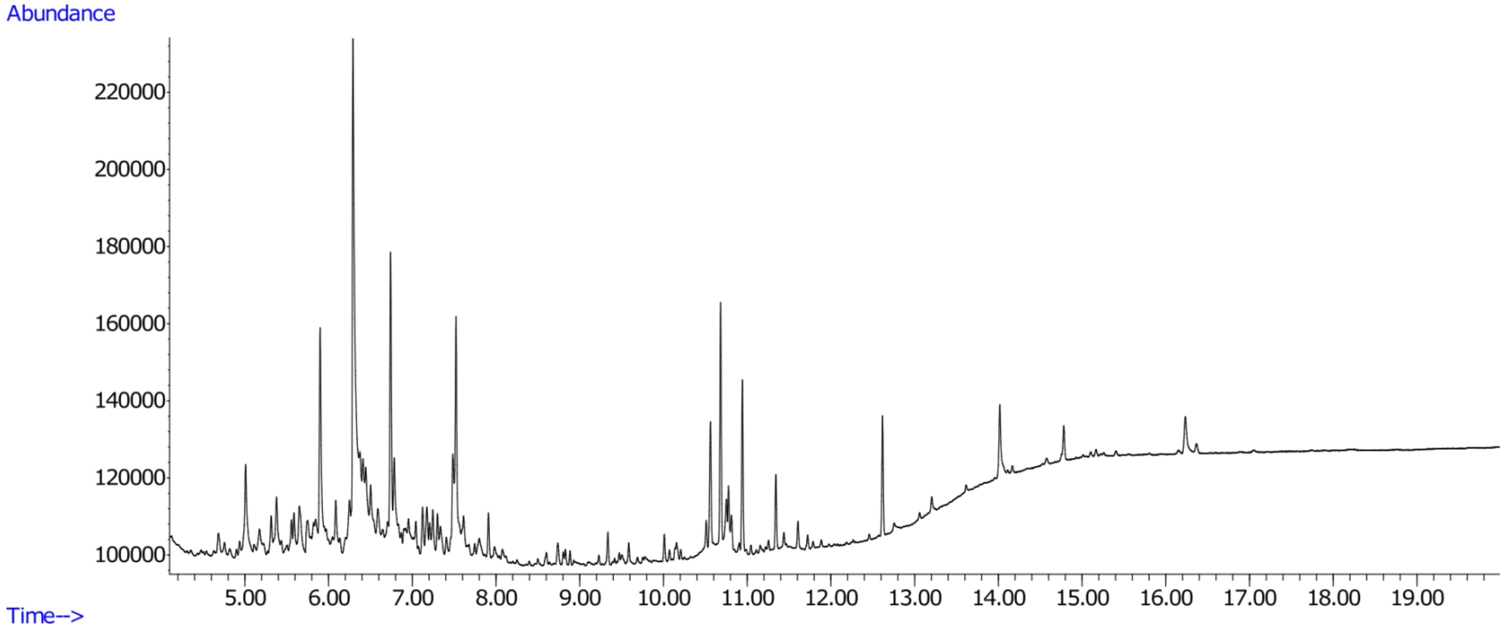

Negative control of substrate 12, biotransformation conditions without any enzyme addition:

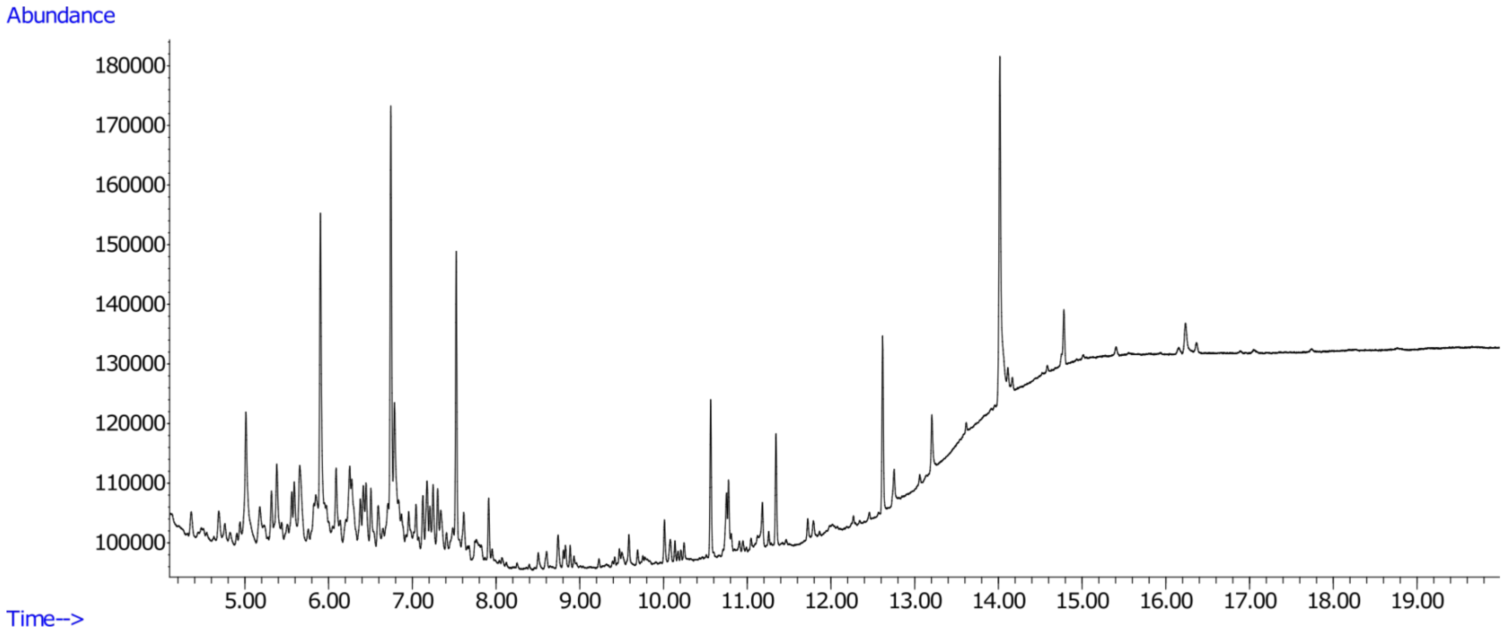

Positive control of every enzyme, using biotransformation conditions and regular FPP as substrate:

Positive control of **PenA:**

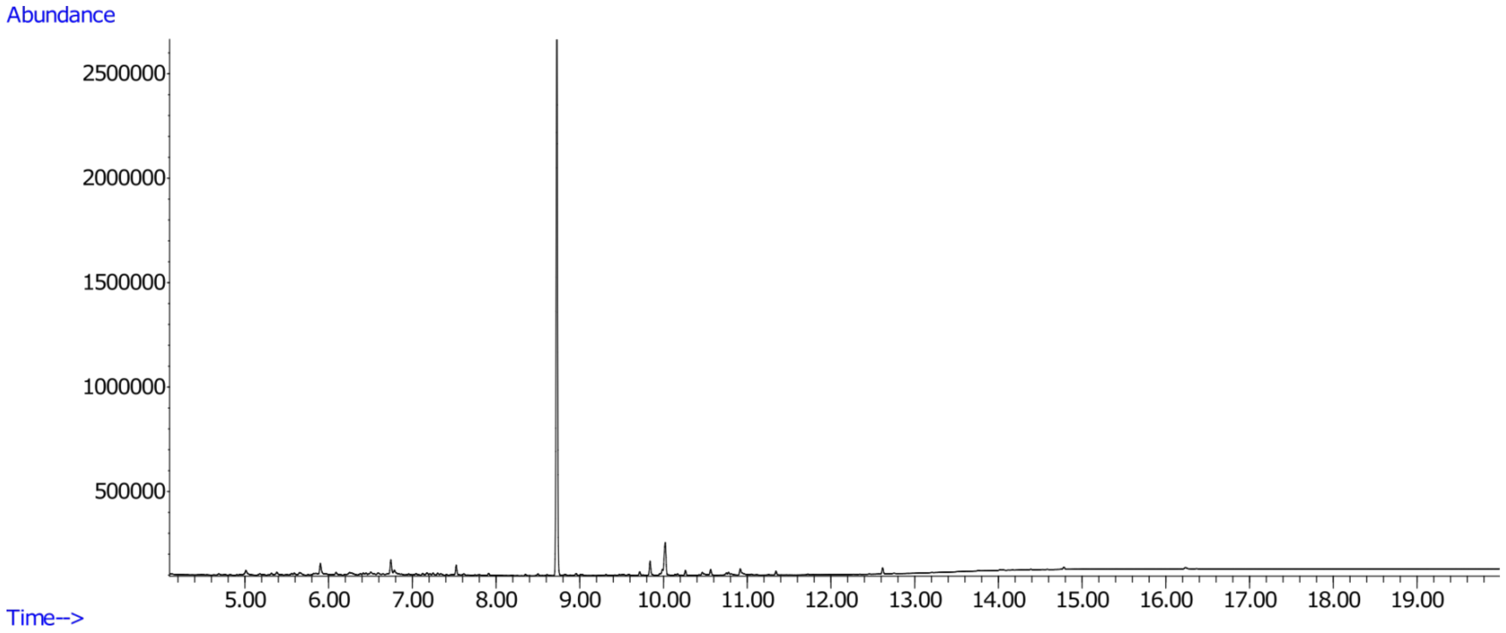

Positive control of **GcoA:**

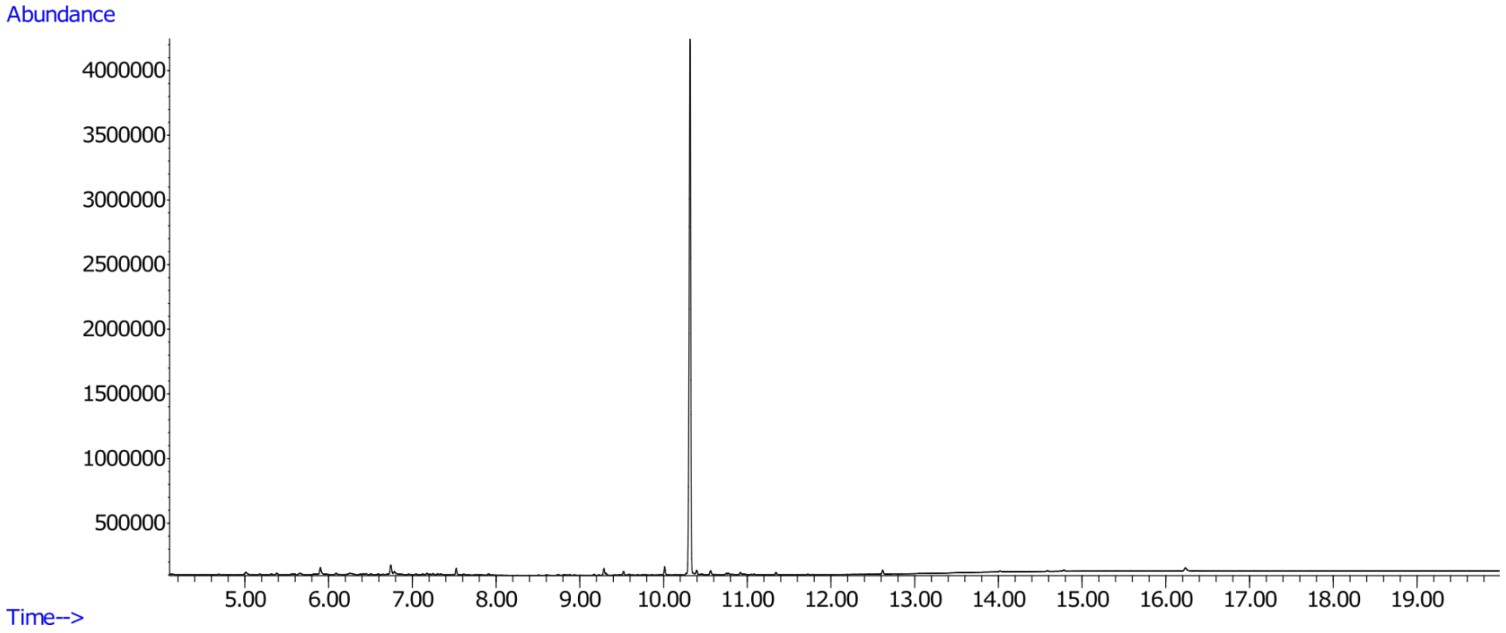

Positive control of **Omp7:**

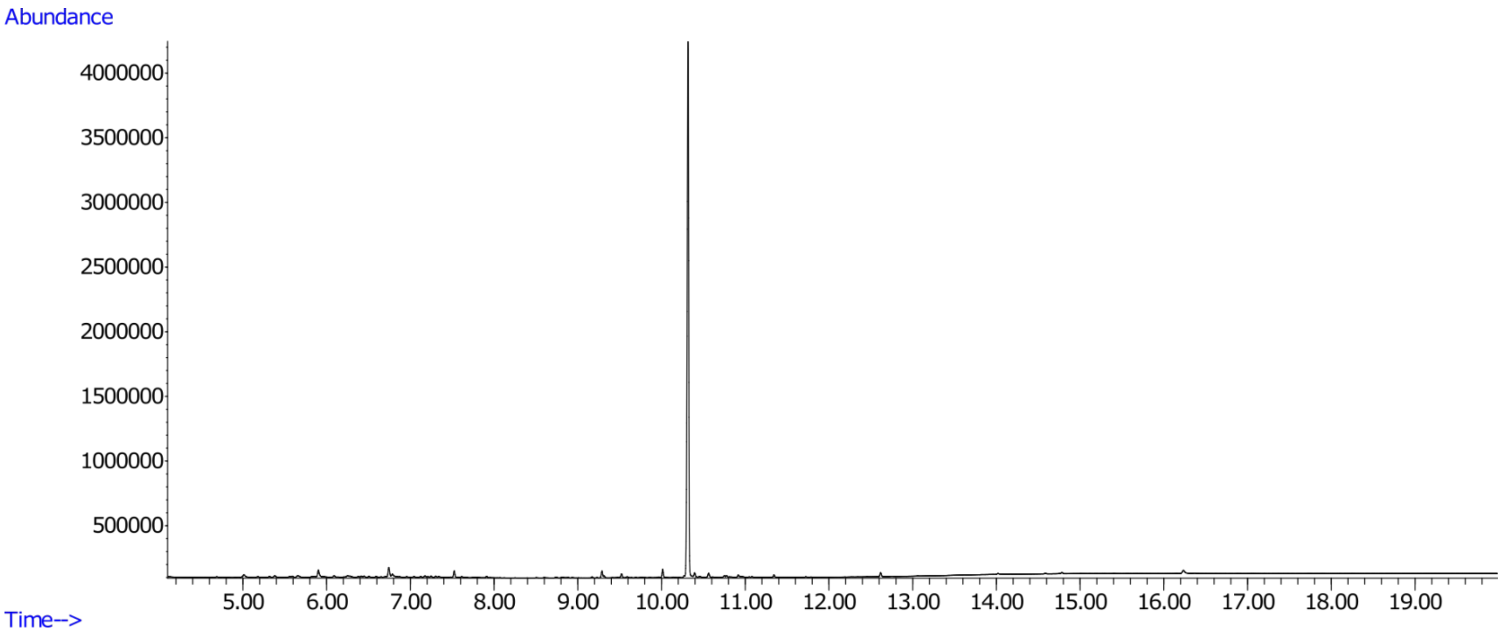

Positive control of **Cop4:**

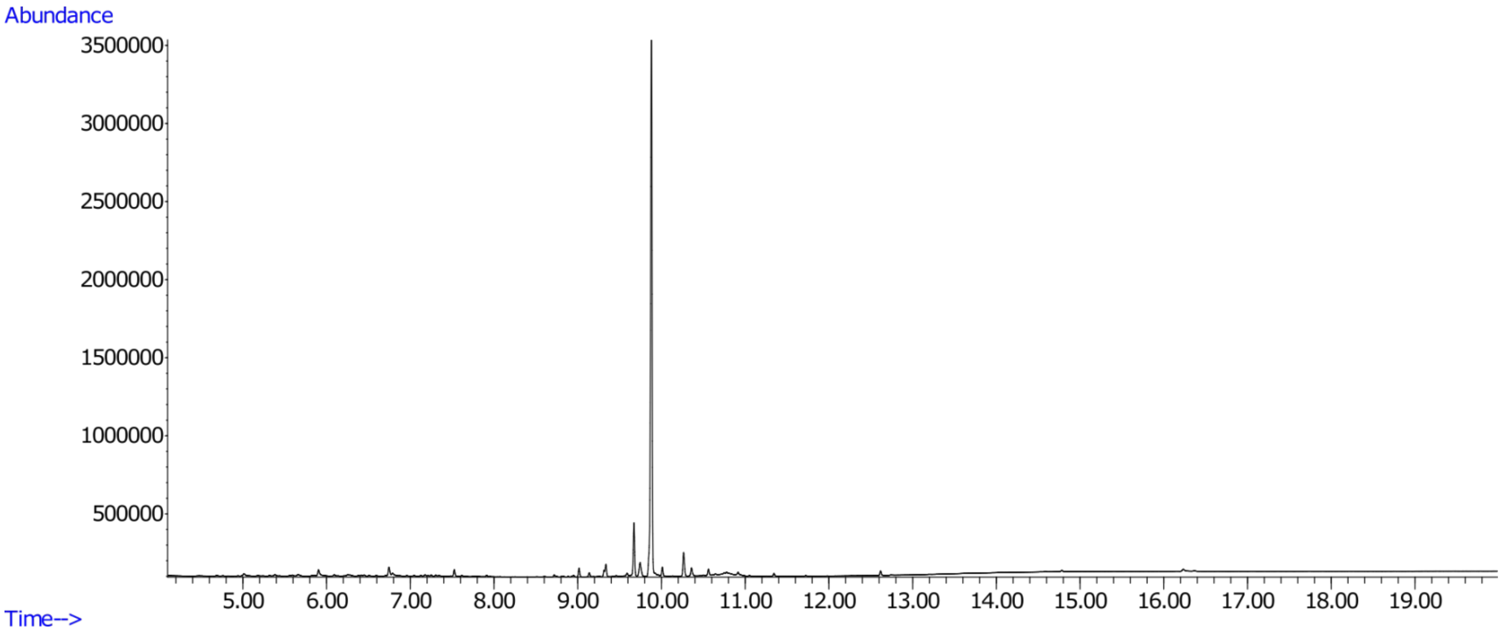

Positive control of **BcBOT2:**

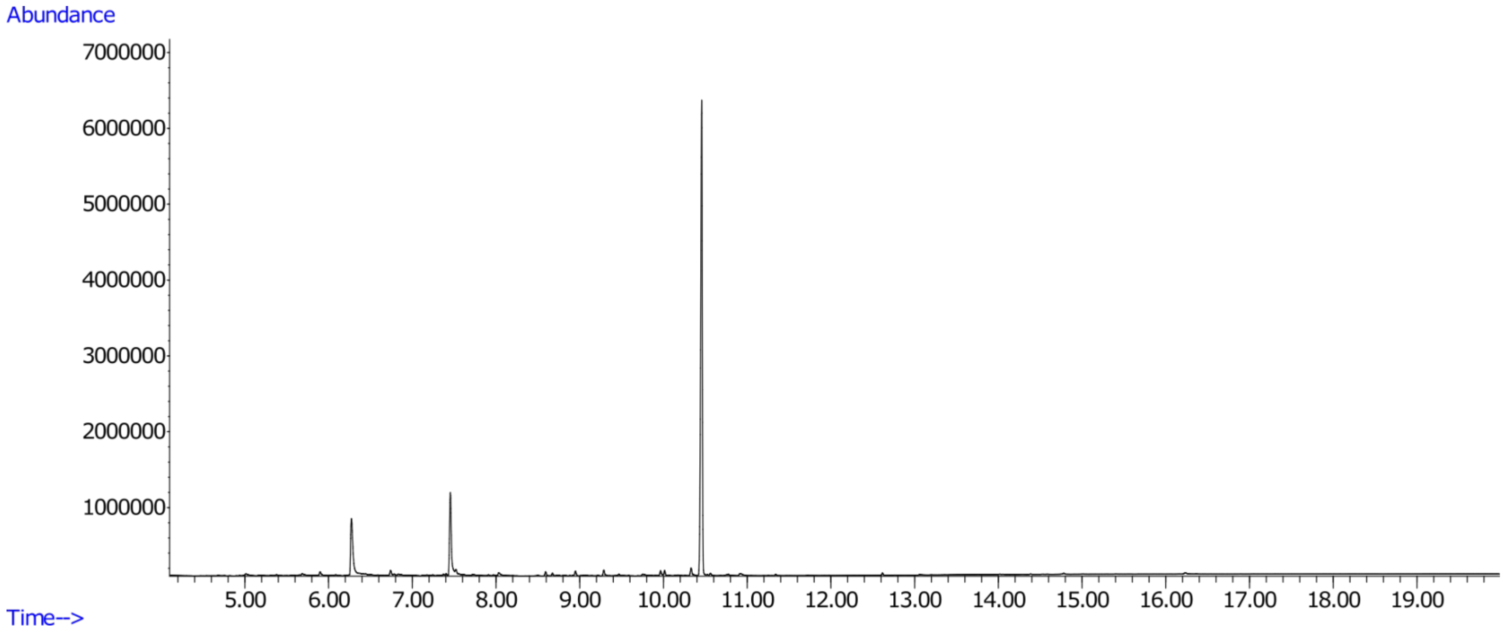

Supplement: Supplementary file 1 [file np5c00409_si_001.pdf]
